# Quality of Evidence in Remote Monitoring of Patients With Liver Cirrhosis: A Systematic Review

**DOI:** 10.1016/j.gastha.2026.100997

**Published:** 2026-05-08

**Authors:** Britt van Ruijven, Marten A. Lantinga, Joost P.H. Drenth, Marieke J. Pierik, Tom J.G. Gevers, Govert Veldhuijzen

**Affiliations:** 1 Department of Gastroenterology and Hepatology, Maastricht University Medical Center, Maastricht, the Netherlands; 2 Division Liver & Digestive Health, Institute of Nutrition and Translational Research in Metabolism (NUTRIM), Maastricht University, Maastricht, the Netherlands; 3 Department of Gastroenterology and Hepatology, Amsterdam UMC, University of Amsterdam, Amsterdam Gastroenterology Endocrinology Metabolism, Amsterdam, the Netherlands; 4 Department of Gastroenterology and Hepatology, Gelre Ziekenhuizen, Apeldoorn, the Netherlands

**Keywords:** Telehealth, Telemedicine, e-Health, End-Stage Liver Disease

## Abstract

**Background and Aims:**

Remote monitoring (RM) enables out-of-hospital surveillance and may benefit patients with liver cirrhosis. However, implementation in routine liver cirrhosis care is limited, most likely due to the lack of conclusive data on clinical effectiveness. This systematic review aims to analyze the quality of evidence regarding the effect of RM tools on disease outcomes for liver cirrhosis.

**Methods:**

This Preferred Reporting Items for Systematic Reviews and Meta-Analyses (PRISMA)-guided systematic review included studies of RM interventions per the World Health Organization’s definition in patients with liver cirrhosis reporting at least 1 liver disease-related outcome. Retrospective or cross-sectional studies were excluded. Risk of bias was assessed using the Cochrane Risk Of Bias Tool for Randomized Trials and the Newcastle-Ottawa Scale for other study types. RM development quality was evaluated against the V3+ framework.

**Results:**

One randomized controlled trial, 4 prospective cohorts with controls, and 7 prospective studies without controls were identified (n = 12; 636 patients; 17,680 studies screened). All studies with controls reported lower hospital readmissions in the RM groups. Interventions included smartphone applications, with or without additional measurement instruments, and automated calls. RM development quality varied substantially, with multiple RM interventions not reporting on verification (n = 6), usability (n = 4), or analytical validation (n = 5). The identified evidence informed the synthesis of a proposal framework to harmonize future RM research.

**Conclusion:**

Although studies show clinical benefits of RM tools in liver cirrhosis, evidence is limited by the (inherent) heterogeneity in interventions, design, and outcomes, and quality of the RM development process. This review emphasizes the need for consensus on RM monitoring frameworks and high-quality data to inform better clinical decision-making.

## Graphical abstract

**Figure:**
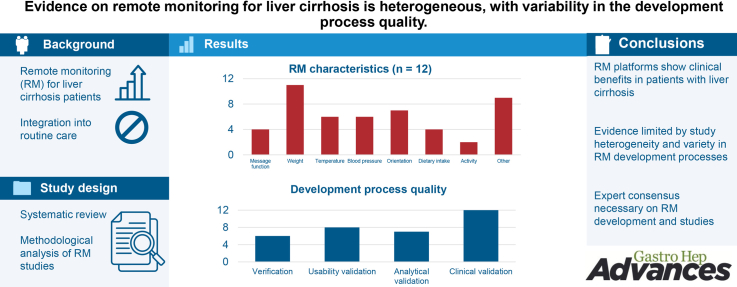

## Introduction

Healthcare delivery, including the management of liver cirrhosis, is increasingly incorporating telehealth, particularly through remote monitoring (RM).^1^, ^2^, ^3^, ^4^, ^5^ RM facilitates the surveillance of a patient’s condition outside the hospital, using technologies such as connected sensors and digital platforms.^6^ In patients with liver cirrhosis, RM platforms have demonstrated promising findings regarding disease-related benefits and cost-effectiveness.^5^

RM can support patient empowerment and could help in the earlier recognition of subtle changes preceding decompensation events in patients with liver cirrhosis. Several prospective studies and reviews have emphasized the potential of RM tools for various liver disease-related events.^5^^,^^7^ Despite encouraging early results, RM remains inconsistently integrated into routine liver cirrhosis care. Substantial heterogeneity in study designs, RM modalities, and reported outcomes constrains the synthesis of conclusive findings and has recurrently been acknowledged as a barrier for real-world translation and successful implementation.^8^, ^9^, ^10^, ^11^, ^12^, ^13^ Available systematic reviews on this topic focused on patients with decompensated liver cirrhosis while excluding studies without a control group or short follow-up, potentially missing out on promising technologies, or included populations with different needs, such as those infected with hepatitis C virus (HCV) without cirrhosis or post-transplant patients.^9^, ^10^, ^11^, ^12^, ^13^ Furthermore, current evidence has not yet been evaluated against structured quality validation frameworks for the development of digital tools.

This systematic review aims to provide a comprehensive methodological analysis of the quality of evidence and existing knowledge gaps regarding the effect of RM tools on disease outcomes in compensated and decompensated liver cirrhosis. In addition, it assesses current evidence against a structured quality framework for validation of digital tools and proposes a framework informed by the identified evidence base to harmonize future research and improve the impact of RM interventions on liver cirrhosis patient care.

## Methods

### Search Strategy

For this systematic review, the Preferred Reporting Items for Systematic Reviews and Meta-Analyses (PRISMA) guideline for reporting systematic reviews was used (Supplementary Table 1).^14^^,^^15^ The search strategy was developed in collaboration with a librarian with expertise in health sciences literature to optimize sensitivity. This review was registered in PROSPERO, an international systematic review registry produced by the Centre for Reviews and Dissemination (CRD), with the number CRD42024620147.

The following databases were searched, with the final search on January 14, 2026: Medline via Ovid, Embase via Ovid, Web of Science, Cochrane Library, the PROSPERO database of systematic reviews, ClinicalTrials.gov, CIHNAHL via EBSCO, and Epistemonikos Health Evidence. Clinicaltrialsregister.eu and the International Clinical Trials Registry Platform Search Portal were screened to identify studies in preparation. Snowballing was performed on titles in the bibliographies of eligible studies. We did not use any date restrictions. Search strategies for each database and deduplication strategies are outlined in Supplementary Tables 2 and 3.

### Study Selection and Eligibility Criteria

B.R. and T.G. independently screened titles and abstracts via Covidence systematic review software; both reviewers were blinded to each other’s decisions.^16^ Full texts were reviewed if the reviewers could not determine a study’s eligibility through title and abstract screening. Any disagreements were resolved through consensus meetings with a third reviewer (G.V.) when necessary.

Studies evaluating patients with liver cirrhosis were selected. Studies assessing patients living with hepatitis B virus and HCV, metabolic dysfunction–associated steatotic liver disease (MASLD), alcohol-related liver disease (ALD), and autoimmune hepatitis patients were included if separate liver cirrhosis data were available. We anticipated that many studies encompassed pilot phases; therefore, studies were also eligible for inclusion even if they did not include a comparative group. The intervention must consist of technologies used for RM, for example, electronic portals, wearables, digital questionnaires, or message functions. We used the World Health Organization's definition of RM: services that enable health-care providers to monitor an individual’s condition remotely, using technologies such as implanted devices and sensors with wireless or wired connections.^6^ Publications required evaluation of at least 1 clinical outcome, reported as either primary or secondary outcomes: liver cirrhosis–related complications (hepatic encephalopathy, [refractory] ascites, gastrointestinal bleeding, acute kidney injury-hepatorenal syndrome), hospital admissions, mortality, transjugular intrahepatic portosystemic shunt–free survival, transplant-free survival, or treatment adherence.

### Study Designs

We included the following study designs: randomized controlled trials (RCTs), non-RCTs, prospective cohort studies, routine health-care databases, uncontrolled and controlled before-and-after studies (including interrupted time series), case-control studies, observational studies, epidemiological studies, experimental or quasi-experimental studies, and feasibility studies. Studies with alternative study designs were evaluated for eligibility in a consensus meeting. We assessed individual studies used in reviews and meta-analyses for eligibility.

We excluded retrospective and cross-sectional study designs, as they are less suitable for assessing the causal relationship between interventions and clinical outcomes, the focus of this review.^17^ Additional exclusion criteria comprised no availability in the English language, assessment of first-line RM without involvement of gastroenterologists/hepatologist, exclusive assessment of telephone or video consultations between patient and health-care provider, between patients, or between health-care providers, and insufficient data.

### Data Extraction and Study Quality Assessment Methods

Data were collected in standardized data extraction forms in Excel. For homogeneity purposes, proof-of-concept studies and (prospective) pilot studies without a control group were recorded as feasibility studies. Follow-up periods were converted to days, with 1 week representing 7 days and 1 month representing 30 days. Nonalcoholic fatty liver disease or nonalcoholic fatty liver disease terms for underlying etiologies were collectively reported as metabolic dysfunction–associated steatohepatitis and MASLD etiologies. We separately defined liver cirrhosis etiologies ALD, MASLD, metabolic and alcohol-related liver disease, as lifestyle-related causes and grouped other etiologies.

Quality of RCTs was assessed with the Cochrane Risk Of Bias Tool for Randomized Trials (RoB 2, Supplementary Figure 1).^18^ All other study types received quality assessment using the Newcastle-Ottawa Scale (NOS) Quality assessment scores of observational cohort studies, a 0–9 scale (Supplementary Material 1, and Supplementary Table 4).^19^ We defined “poor,” “fair,” and “good” quality as total NOS scores of 0–3, 4–6, and 7 or higher, respectively.

### Remote Monitoring Development Quality Assessment Methods

RM interventions in studies were evaluated against the recently updated and widely adopted Digital Medicine Society’s V3+ framework.^20^^,^^21^ The Digital Medicine Society’s V3+ framework provides one of the most widely adopted structures for the development process of sensors, also applicable to many RM systems.^20^^,^^21^ The framework distinguishes 4 phases in the development process of digital health technologies: (1) verification, evaluating the performance or output of data of the RM tool against prespecified criteria; (2) usability validation, reporting whether the technology can be used with ease, efficiency, and user-satisfaction to achieve specified goals; (3) analytical validation, assessing whether the platform accurately measures, detects, or predicts physiological or behavioral metrics; and (4) clinical validation, which determines whether use of the technology leads to meaningful clinical benefits in the target population and situation. We assessed if included RM studies answered 4 questions, each representing a phase of the V3+ framework (Supplementary Table 6).^20^, ^21^, ^22^ If studies referred to previously conducted research in which one or more of the phases of the framework were reported, we reported that they complied with the respective phase of the framework. We reported the verification phase as performed in case a quality certification was available, or the RM tool was federal regulation compliant, also if this data was not open-sourced.

### Proposal for a Framework of Development and Evaluation Trajectories of Remote Monitoring Studies in Liver Cirrhosis

The included studies were categorized and synthesized to provide an overview of prevailing methodologies and to identify gaps in the current evidence base. We especially examined the key study characteristics, including study designs, recruitment sites, outcomes, singlecenter vs multicenter designs, the availability of a validation cohort, and follow-up duration.

Based on these observations, we aimed to move toward a more structured interpretation of the existing evidence by deriving an author-based consensus framework. This framework intends to help structure the development and evaluation of RM interventions in liver cirrhosis.

## Results

### Literature Search

**Figure 1:**
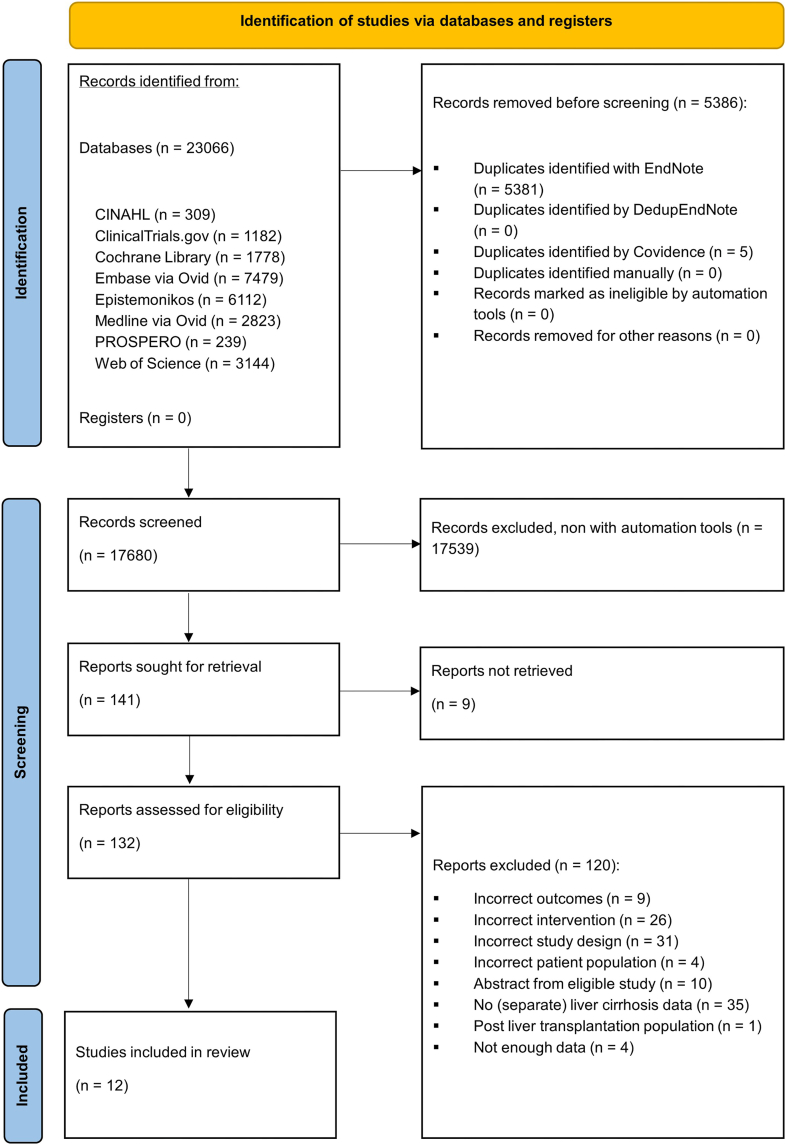
Preferred Reporting Items for Systematic Reviews and Meta-Analyses (PRISMA) 2020 flow diagram. Presenting the identification of 17,680 screened records and 12 included studies.

Our search strategy identified 17,680 individual studies. We included a total of 12 studies (n = 8 full papers, n = 4 abstracts, Figure 1). Snowballing through citation tracking did not yield any additional inclusions.

### Study Designs

The study designs included 1 RCT, 4 prospective cohorts with control groups, and 7 prospective cohorts without controls (Table 1). The majority of patients with liver cirrhosis were hospitalized at the time of inclusion (9/12). The primary study setting was a single academic liver transplant center (10/12). All control groups consisted of internal cohorts, of which 2 were historic populations. Follow-up durations ranged from 28 to 90 days. The RCT was a follow-up study on an open-label feasibility study with the Patient Buddy App and scored a “low risk of bias” in the Cochrane Risk of Bias Assessment Tool of RCTs (Table 1; Supplementary Figure 1).^23^ Cohort studies were rated with “good” (n = 2) and “fair” (n = 9) overall quality, respectively, through the NOS (Supplementary Table 4).

**Table 1:** Study Designs

Design	Year	Author	Inpatient or outpatient recruitment	RM intervention	Primary outcome	Setting	Validation cohort	Follow-up duration
Randomized controlled trial	1. 2025	Shaw et al^23^	Inpatient	Smartphone application (Patient Buddy App and EncephalApp)	Avoidable readmissions	Three transplant centers, of which 2 academic centers	Internal	30 d
Prospective cohort with control group	2. 2023	Kazankov et al^7^	Inpatient	Smartphone application (CirrhoCare) with monitoring devices: (1) Wristwatch (Withings Move) (2) Blood pressure (BP) cuff (3) Weighing scales (Withings Body+) with bioimpedance	Feasibility of daily remote management	Single academic transplant center	Internal, matched controls	84 d
	3. 2024	Penrice et al^24^	Inpatient	Tablet with Bluetooth-enabled vital signs uploads from blood pressure monitor, pulse oximeter, and weighing scale	Qualitative aspects of design,	implementation, and patient satisfaction	Single academic transplant center	Internal, matched retrospective controls 90 d
	4. 2017	Khungar et al^25^ Abstract	Inpatient	Tablet with wireless blood pressure monitor, pulse oximeter, and weighing scale	*Not reported*	Single academic transplant center	Internal	90 d
	5. 2024	Ballesteros et al^26^ Abstract	Inpatient	Smartphone application (DOCCLA’s digital platform)	*Not reported*	Single academic transplant center	Internal, retrospective controls	42 d intervention 90 d follow-up
Prospective cohort without control group	6. 2022	Lin et al^27^	Outpatient	Wrist personal activity trackers (PAT) and smartphone application (EL-FIT)	Incidental hospital admission and mortality	Single academic transplant center.	No	*Predefined follow-up not reported. Median follow-up 223 d.*
	7. 2015	Thomson et al^28^	Inpatient	Proactive automated scheduled calls	Time to first hospital admission	Single academic transplant center	No	90 d
	8. 2025	Ngu et al^29^	Inpatient and outpatient	Smartphone application (LivR well) with Bluetooth weighing scales, and a cloud-based database for referral, screening and data collection	Acceptability and feasibility	Single academic liver transplant center	No	28 d intervention 84 d follow-up
	9. 2020	Bloom et al^30^	Inpatient and outpatient	Smartphone application with Bluetooth weighing scale connected to EMR	% enrolled d during which weight data were successfully transmitted to the EMR and the % weight alerts that prompted a response by the provider	Single academic transplant center	No	*Predefined follow-up: 28 d.* *Actual follow-up: 979 patient-d.*
	10. 2021	Qian et al^31^ Abstract	Inpatient	*Specific devices not reported.*	*Not reported*	Single academic transplant center	No	90 d
	11. 2018	Verma et al^32^ Abstract	Inpatient	Tablet application with web-based provider portal to retrieve the results	*Not reported* *Overall purpose: assess feasibility*	*Not reported*	No	90 d
	12. 2017	Ganapathy et al^33^	Inpatient	Smartphone application (Patient Buddy App)	Total 30-d admissions, HE-related readmissions, dropouts	Single academic transplant center	No	30 d

EL-FIT, Exercise and Liver FITness; EMR, electronical medical record.

### Population Characteristics

Sample size in the RM group ranged from 19 to 116 patients (Table 2). Baseline disease severity was mostly reflected in the mean and median Model for End-stage Liver Disease or Model for End-stage Liver Disease-sodium scores, with a range of 13–19.5. The mean or median age ranged from 51 to 67.2 years. A total of 636 patients participated in RM, of whom the majority were male (62.9%). ALD was the predominant etiology of liver cirrhosis (36%, 207/583 reported), 164 (28%) were known with metabolic dysfunction–associated steatohepatitis or MASLD, and 212 (36%) had other etiologies.

**Table 2:** Population Characteristics

Author	Inpatient or outpatient recruitment	N (control group vs intervention group)	Disease status at baseline (control group vs intervention group)	% liver cirrhosis etiologies (control group vs intervention group)		% males (control group vs intervention group)	Age (y) (control group vs intervention group)
Shaw et al^23^	Inpatient	116 vs 116 dyads^c^	MELD-Na: 18.9 ± 8.4 vs 16.9 ± 7.9^a^	Alcohol MASH/MASLD Other	31.0 vs 31.0 26.7 vs 28.4 42.2 vs 40.5	65.5 vs 68.9	59.1 ± 12.38 vs 58.4 ± 8.88^a^
Kazankov et al^7^	Inpatient	20 vs 20	MELD-Na: 18 ± 4.6 vs 16.1 ± 4.2^a^	Alcohol MASH/MASLD Other	85 vs 80 10 vs 10 5 vs 10	45 vs 70	56 ± 14 vs 59 ± 10^a^
Penrice et al^24^	Inpatient	74 vs 41	MELD-Na: 17.3 (13.0–18.42) vs 15.1 (9.8–18.3)^b^	Alcohol MASH/MASLD Other	52.3 vs 46.3 30.1 vs 26.8 17.6 vs 26.8	54.0 vs 58.5	62.4 ± 12.5 vs 60.9 ± 9.0^a^
Khungar et al^25^ Abstract	Inpatient	143 vs 19	Median MELD-Na: 19 % Child-Pugh B: 57.9 % Child-Pugh C: 31.6 *Not reported for control group*	Alcohol MASH/MASLD Hepatitis C	*Not reported* *Not reported* 53	*Not reported for control group* % males intervention group: 47.4	*Not reported for control group* Median age intervention group: 58
Ballesteros et al^26^ Abstract	Inpatient	39 vs 43	Decompensated patients with ascites. *MELD-Na or Child-Pugh score not reported*	*Not reported.*		41 vs 65	*Not reported.*
Lin et al^27^	Outpatient	116: 71 PAT, 45 PAT + EL-FIT application	MELD: 14.3 ± 5.9^a^ MELD-Na: 15.2 ± 6.9^a^ Child-Pugh score: 8 (5–10)^b^	Alcohol MASH/MASLD Other	33 30 37	55	56 ± 11^a^ *Inclusion criteria: age 40–70 y*
Thomson et al^28^	Inpatient	79	Mean MELD: 13 (6–40) Mean Child-Pugh score: 8 (6–13)	Alcohol MASH/MASLD/cryptogenic Other	11 39 49	51	57 (23–85)^b^
Ngu et al^29^	Inpatient and outpatient	59	Patients with ACLF MELD: 16 (12–21)^b^ % Child-Pugh A: 22 % Child-Pugh B/C: 78 *Inclusion criteria: MELD ≥ 10*	Alcohol MASH/MASLD Other	74 5 21	66	51 (37–59)^b^
Bloom et al^30^	Inpatient and outpatient	25	Patients actively receiving ascites management. MELD: 15.8 ± 5.9^a^	Alcohol MASH/MASLD Other	44 36 20	48	57.6 ± 12.8^a^
Qian et al^31^ Abstract	Inpatient	48 17 dropped out immediately	Mean MELD-Na: 13.5 (6–24) % Child-Pugh A: 33 % Child-Pugh B/C: 67	Alcohol MASH/MASLD Other	34 50 16	100	Mean age: 67.2 (47–78)
Verma et al^32^ Abstract	Inpatient	30	Mean MELD-Na: 19.5 (11–29) Inclusion criteria: ≥1 liver disease related complication. Exclusion criteria: patients discharged to nursing homes.	Alcohol MASH/MASLD Other	40 10 50	60.0	Mean age: 55.7 (28–71)
Ganapathy et al^33^	Inpatient	40	MELD: 19.5 ± 5.2^a^	Alcohol MASH/MASLD Other	17.5 32.5 50	60	58 ± 10^a^

EL-FIT, Exercise and Liver FITness; MASH, metabolic dysfunction–associated steatohepatitis; MELD-Na, Model for End-stage Liver Disease-sodium; PAT, physical activity tracker.

a Mean ± SD.

b Median (IQR).

c Dyad: a cirrhosis inpatient and an adult caregiver.

### Intervention Characteristics of Remote Monitoring

Seven studies employed smartphone applications as RM intervention, of which 4 incorporated additional wireless sensors/wearables, including weighing scales and physical activity trackers. Tablet applications were utilized in 3 studies. One study relied on automated, scheduled calls, while another did not specify the RM tool used, although heart rate, blood pressure, and weight were monitored (Table 3). Continuous monitoring was implemented in 1 study, whereas 5 studies applied daily monitoring protocols. Two reported weekly monitoring, and 1 publication described RM conducted 3 times per week in conjunction with various paramedical consultations. Body weight was the most frequently assessed parameter, being reported in 11 RM interventions. A message function was available in 4 RM tools. Most tools assessed multiple clinical or behavioral parameters, like orientation (7), temperature (6), blood pressure/heart rate (6), dietary intake (4), and physical activity (2).

### Study Aims and Reported Outcomes

Two studies primarily evaluated the effect of RM on clinical outcomes.^23^^,^^26^ Seven studies primarily aimed to pilot or define the feasibility of the RM intervention, for example, evaluate whether patients and health-care providers are willing and able to use the RM tool (Table 3). Two studies assessed both feasibility and RM effectiveness,^24^^,^^33^ and 1 study did not report on the primary research aim.^31^ Primary outcomes ranged from avoidable readmissions to incidental hospital or total admissions, and time to hospital admission (4/12), to feasibility and related subdomains (5/12). In 3 abstracts, primary outcomes were not specifically reported (Table 1). All studies addressed hospital (re)admissions after inclusion (Table 4). Half of the studies recorded patient contacts, medication changes, or other interventions and mortality, while adherence and satisfaction were outlined in 7 and 8 publications, respectively (Table 4). They all documented positive results regarding adherence and satisfaction, but the reporting variety limited a summary synthesis of these outcomes in this review.^7^^,^^23^^,^^24^^,^^28^, ^29^, ^30^, ^31^, ^32^, ^33^ Only Ngu et al presented specific cost outcomes of the intervention and assessed quality of life.^29^

**Table 3:** Intervention Characteristics of Remote Monitoring

Author	Aim	RM frequency	Message function	Weight	Temperature	Blood pressure and heart rate	Orientation	Dietary intake	Activity	Other:
Shaw et al^23^	Reducing 30-d readmissions.	Daily monitoring: Weight and temperature. Weekly monitoring: Other parameters.	✓	✓	✓ Self-reported	x	✓ EncephalApp and judged by caregiver	✓ Sodium intake	x	Self-reported: Bowel movements GI-bleeding Infections Medication adherence
Kazankov et al^7^	Assess feasibility and patient engagement of monitoring advanced cirrhosis.	Daily monitoring	✓ Text and voice messages	✓	x	✓	✓	✓	✓ Wristwatch	x
Penrice et al^24^	1. Design and implement a cirrhosis-specific RPM (CiRPM) program. 2. Show utility through measurement of potential clinical benefit, patient satisfaction, and patient adherence.	Daily monitoring: Parameters. Every 3 d: six alternating liver-complication yes-no questions.	✓	✓	✓	✓	x	x	x	Self-reported: Bowel movements Changes in attention Swollen ankles Abdominal tension Shortness of breath Missed medication
Khungar et al^25^ Abstract	Pilot a wireless mobile device monitoring system to detect early symptoms and signs, thereby preventing readmissions and keeping patients engaged and feeling cared for daily.	*RM frequency not reported*	*Not reported*	✓	x	✓	✓	x	x	Self-reported: Hepatic encephalopathy Fluid overload GI bleeding Infections Medication adherence *All not specifically reported.*
Ballesteros et al^26^ Abstract	Demonstrate the impact of real-world remote monitoring of patients with decompensated cirrhosis.	*RM frequency not reported*	*Not reported*	✓	✓	✓	*Not reported*	*Not reported*	*Not reported*	Liver-related symptoms/complaints, *not specifically reported.*
Lin et al^27^	Assess the utility of daily step count in predicting hospital admission and mortality rates.	Continuous monitoring and promotion of exercise training	x	x	x	✓ Heart rate	x	x	✓	x
Thomson et al^28^	Test the IVR system and determine if gathered information can predict complications.	Weekly IVR calls.	x	✓ Self-reported	x	x	✓ Self-reported	x	x	Self-reported: Swelling Jaundice Weakness Medication changes Overall health
Ngu et al^29^	Investigate the acceptability and feasibility of a multimodal community intervention for ACLF.	Three times per week, home nursing. Weekly medical review. “Regular” dietitian, and pharmacy consultations, with adjunct interventions by physiotherapy, social work, addiction medicine, or neuropsychiatry.	*Not reported*	✓	x	x	x	x	x	Home nursing to assess liver-related symptoms Medical review and urine (ETG) sample to assess alcohol use.
Bloom et al^30^	Evaluate the feasibility of a smartphone app in facilitating outpatient ascites management.	Every weekday: Weight monitoring	x	✓	x	x	x	x	x	x
Qian et al^31^ Abstract	*Not reported*	*RM frequency not reported*	*Not reported*	✓	✓ Self-reported	✓	✓ Self-reported	✓ Self-reported salt intake	x	Self-reported: Bowel movements Swelling Shortness of breath Dizziness Falls Pain Medication Alcohol use
Verma et al^32^ Abstract	Assess the feasibility and utility of TeleMonitoring of Symptoms and Cognitive function/TeMSCog for ESLD inpatients.	Daily questions about symptoms on a 0–10 scale, including pain, depression, and anxiety	*Not reported*	✓	✓	x	✓ EncephalApp	x	x	Liver-related symptoms/complaints, *not specifically reported.* Self-reported: Breathlessness Pain Depression Anxiety Fatigue
Ganapathy et al^33^	Define the feasibility of a smartphone app and its impact on 30-d readmissions.	Daily monitoring: Weight and temperature. Weekly monitoring: Other parameters	✓	✓	✓ Self-reported	x	✓ EncephalApp and judged by caregiver	✓ Sodium intake	x	Self-reported: Bowel movements GI-bleeding Infections Medication adherence Judged by caregiver: Fall risk Time up and go test

ACLF, acute on chronic liver failure; CiRPM, Cirrhosis-specific Remote Monitoring Program; ESLD, end-stage liver disease; ETG, ethyl glucuronide; GI, gastrointestinal; IVR, interactive voice response; TeMSCog, TeleMonitoring of Symptoms and Cognitive function.

**Table 4:** Reported Outcomes

Author	Hospital (re)admissions	Patient contacts, medication changes, or interventions	Mortality	Liver transplantation or workup	RM adherence	RM evaluation by patients	Other reported outcomes
Shaw et al^23^	✓ Including avoidable readmissions^a^	✓	x	✓	✓	✓	LVPs
Kazankov et al^7^	✓	✓	✓	✓	✓	✓	Unplanned LVPs Changes MELD-Na and CLIF-C AD scores
Penrice et al^24^	✓	x	✓	x	✓	✓	LVPs
Khungar et al^25^ Abstract	✓ Including avoidable readmissions^a^	x	x	x	x	x	x
Ballesteros et al^26^ Abstract	✓^a^	✓^a^	✓^a^	x	x	x	Planned LVPs Decompensation rate
Lin et al^27^	✓	x	✓	x	x	x	Daily step count association with hospital admission and mortality, adjusted for MELD-Na and EL-FIT use
Thomson et al^28^	✓	x	✓	✓	✓	x	x
Ngu et al^29^	✓	x	✓	✓	✓	✓	Changes MELD-Na and Child-Pugh scores Sarcopenia incidence CLDQ and EuroQoL VAS scores (self-rated health questionnaires) Costs
Bloom et al^30^	✓	✓	x	x	✓	✓	LVPs
Qian et al^31^ Abstract	✓	x Mentioned, but no specific data reported	x	x	✓	x Mentioned, but no specific data reported	x
Verma et al^32^ Abstract	✓	✓	x	✓	x Mentioned, but no specific data reported	✓	x
Ganapathy et al^33^	✓	✓	x	✓	✓	✓	RM measurements: Changes in EncephalApp scores and up and go times

CLDQ, Chronic Liver Disease Questionnaire; CLIF-AD, Chronic Liver Failure Consortium Acute Decompensation; EL-FIT, Exercise and Liver FITness; LVP, large-volume paracentesis, MELD-Na, Model for End-stage Liver Disease-sodium.

a *P* value <.05 in favor of intervention group.

### Clinical Outcomes

All 5 studies with control groups demonstrated clinical benefits of RM. Although not powered for clinical outcomes, 3 reported statistically significant benefits regarding (preventable) hospital readmissions compared to the control group, ranging from 10 vs 19.8% after 30 days to 12 vs 28% (Table 4, Supplementary Table 5).^23^^,^^25^^,^^26^ Ballesteros et al also presented a significant reduction in large-volume paracentesis and mortality.^26^ Kazankov et al and Penrice et al reported similar positive trends in readmission rates, large-volume paracenteses, and mortality.^7^^,^^24^ Thirty-day hospital readmission rates ranged from 15% to 42.5% in studies without a control group^29^^,^^33^ and from 3.9% to 68% in studies with follow-up until 90 days (Supplementary Table 5).^27^^,^^28^^,^^30^, ^31^, ^32^

### Remote Monitoring Development Quality Assessment

**Figure 2:**
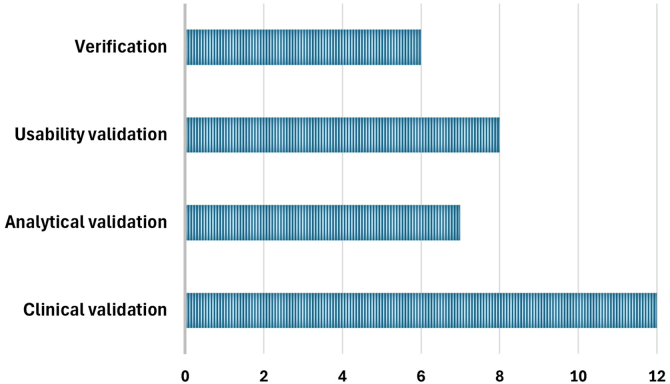
Number of studies reporting on remote monitoring development. Phases: verification (n = 6), usability validation (n = 8), analytical validation (n = 7), and clinical validation (n = 12).

The Patient Buddy App, investigated by Shaw et al and Ganapathy et al, and the CirrhoCare platform of Kazankov et al presented reports on progress through all 4 phases of the V3+ quality framework (Figure 2, Supplementary Table 6, see Methods section for explanation of phases).^7^^,^^23^^,^^33^ Half of the studies used an RM tool, of which verification, quantifying the accuracy of a measurement, was traceable.^7^^,^^23^^,^^26^^,^^27^^,^^30^^,^^33^ Eight studies used an RM tool with reported usability, assessing platform ease of use and efficiency.^7^^,^^23^^,^^24^^,^^28^, ^29^, ^30^^,^^33^ Analytical validation, monitoring whether the platform accurately captures the intended outcomes, was reported in RM applications of 7 studies.^7^^,^^23^^,^^24^^,^^27^^,^^30^^,^^32^^,^^33^ Parts of clinical validation were performed in all studies, as they all reported on clinical outcomes of the RM platform in the target population.

### Proposal for a Framework of Development and EEvaluation Trajectories of Remote Monitoring Studies in Liver Cirrhosis

The identified methodological strategies and gaps in the current evidence base synthesized a framework aimed to structure the development and evaluation of RM interventions in liver cirrhosis (Table 5). The framework comprises 4 sequential phases. First, early-phase feasibility studies should be conducted using harmonized methodologies to assess usability, adherence, and technical performance and to inform the selection of RM platforms for further evaluation. Second, prospective clinical evaluation should be undertaken in parallel with implementation efforts, enabling the generation of clinical evidence while facilitating integration into routine care. Third, confirmatory prospective trials should incorporate predefined core outcome sets, extended follow-up periods, multicenter designs, patient-reported outcome measures, patient-reported experience measures, and economic evaluations. Finally, external validation studies should assess generalizability across patient subgroups stratified by disease severity, comorbidity burden, and sociodemographic characteristics.

**Table 5:** Proposal for a Framework of Development and Evaluation Trajectories of RM Studies in Liver Cirrhosis

	Design	Recruitment site	Outcomes	Setting	Validation cohort	Follow-up duration
Most promising RM tools optimization	Feasibility (pilot) study	Inpatient	Primary: • Dropout rate and time to dropout (usability and clinical validation) • Compliance rate (usability validation) • Patient and provider satisfaction through PROMs and PREMs (usability validation) • Number of alerts (verification and analytical validation) • Number of (alert-based) contacts and interventions (analytical and usability validation) • Costs: healthcare consumption, RM use, QALY (clinical validation) Secondary (clinical validation): • Hospital readmission rate • Mortality rate • Decompensation events according to Baveno VII classification	Single or multicenter	Not applicable	≥6 mo
		Outpatient				
	Prospective cohort with control group, randomized or nonrandomized	Inpatient	Primary (clinical validation): • Hospital readmission rate • Mortality rate • Decompensation events according to Baveno VII classification Secondary: • Dropout rate and time to dropout (usability and clinical validation) • Compliance rate (usability validation) • Patient and provider satisfaction through PROMs and PREMs (usability validation) • Number of alerts (verification and analytical validation) • Number of (alert-based) contacts and interventions (analytical and usability validation) • Costs: healthcare consumption, RM use, QALY (clinical validation)	Multicenter: Secondary center, and academic transplant center	Prospective internal or external cohort matched for: age, sex, etiology, MELD-Na and Child-Pugh scores, treatment center, living situation, education, and digital health literacy	≥3 mo
		Outpatient				≥6 mo for decompensated patients at baseline ≥12 mo for compensated patients at baseline
	Prospective cohort comparing different RM characteristics, for example: RM content RM frequency RM configurations, for example: additional vital parameters					

PREM, patient-reported experience measures; PROM, patient-reported outcome measures; QALY, quality-adjusted life year.

## Discussion

### Main Findings

This systematic review shows that RM in clinical care for patients with liver cirrhosis benefits the outpatient management of patients with liver cirrhosis. All studies with a control group, including 1 RCT, reported outcomes favoring RM, most commonly reflecting reductions in hospital readmissions or related healthcare utilization. The majority of included studies primarily assessed feasibility, consistently demonstrating high patient adherence and positive patient-reported experiences. Nonetheless, this review demonstrates the research gaps in RM interventions evaluated for disease outcomes in patients with liver cirrhosis. Most studies were small, single-center feasibility studies, with substantial variety in study design, intervention characteristics, and reported outcomes, limiting assessment of clinical endpoints. Evaluation using the V3+ framework revealed that reporting on verification, usability, and analytical validation was incomplete for several RM tools.

### Signals of Clinical Potential and Feasibility

The included studies consistently demonstrated clinical effects in reducing hospital (re)admissions, as has been acknowledged in previous reviews, indicating a robust and coherent pattern across different study designs and settings.^5^^,^^11^ High adherence and patient satisfaction emphasize the acceptability of digital tools in this population, including patients with lifestyle-related etiologies, supporting RM applicability in real-world contexts.^7^^,^^24^ Importantly, the inclusion of patients with advanced cirrhosis is highly relevant, given the high readmission rates and potential health gains through RM.^34^

### Heterogeneity as a Limiting Factor for Evidence Synthesis

In this study, we observed heterogeneity across different domains, characteristic of an emerging research field still in an exploratory and developmental phase. This includes variation in study objectives (feasibility vs effectiveness), intervention characteristics, outcome measures, and follow-up duration, complicating comparisons across studies. These observations are supported by statements by Gananandan et al, highlighting the need for standardization of digital tools across healthcare settings with generalizable components.^5^ Evidence generation was further constrained by predominantly single-center designs, limited follow-up, and the frequent use of internal or retrospective control groups, limiting causal inference and increasing the risk of selection bias. Finally, the predominance of academic transplant populations and limited reporting on key feasibility determinants, like sociodemographic factors, raises concerns about the generalizability to broader hepatology settings, in compensated, outpatient groups, and low-resource contexts.^9^ Certainly, these restrictions reflect the reality that developing, evaluating, and implementing innovations necessarily involves an iterative and practice-oriented approach.

### Contextualization Within Existing Digital Health Literature

The clinical benefits of RM have been more extensively studied in patients with cardiac failure, as presented by meta-analyses that suggest a more advanced stage of RM development and methodological rigor. Despite this relatively robust evidence base, the evidence diversity has resulted in only a weak recommendation for non-invasive RM in the most recent European guidelines for acute and chronic cardiac failure.^35^ Similarly, heterogeneity in study outcomes has been observed by Kumar et al in RM for patients with decompensated liver cirrhosis or inflammatory bowel disease (IBD).^9^ The extrapolability constraints identified in this review further align with findings in the IBD, chronic obstructive pulmonary disease (COPD), and acute care population, underscoring the pervasiveness of these limitations in the field of digital health across diseases.^8^^,^^36^^,^^37^ This indicates that limited extrapolability likely reflects differences in RM development and scientific evaluation practices rather than disease-specific effects, necessitating a structured assessment of RM development and research.

### Remote Monitoring Development Quality and Implications of the V3+ Framework Assessment

Accordingly, evaluating available RM projects along a systematic quality framework like the V3+ framework clarifies why cross-study comparability remains limited. By distinguishing between different stages of development, the assessment highlights that clinical validation alone is insufficient without reports on verification, usability testing, and analytical validation, aligning with the findings on intervention heterogeneity. As a result, most studies evaluating RM tools in cirrhosis primarily focus on technical feasibility rather than long-term outcomes or cost-utility, explaining the limited ability to identify the most clinically valuable RM configurations. Assessment through this framework underlines why current effect estimates remain preliminary.

### Implications for Future Research

This review proposes a framework for the development and research trajectory of RM studies in liver cirrhosis, derived from the evidence gaps identified, to facilitate harmonization research and generation of comparable evidence. We recommend future research to build on the existing evidence base while explicitly distinguishing feasibility studies from evaluations of disease-related outcomes. Initial studies need to demonstrate reliable RM performance and consistent benefits, which are essential prerequisites for resource-intensive research and implementation strategies. Feasibility studies should prioritize the performance of monitoring of ascites and hepatic encephalopathy through body weight and orientation assessments, as these domains are currently most frequently evaluated (Table 3) and are likely to yield the largest clinical impact in follow-up studies, given their predominance as causes of hospital (re)admissions.^34^^,^^38^ Following confirmation of feasibility and technical reliability, prospective evaluation studies should incorporate core outcomes, long-term follow-up, and multicenter designs. Notably, Muftah et al showed that telehealth in the HCV management achieves clinical outcomes comparable to standard care.^10^ Accordingly, quality-of-care indicators like patient-reported outcome measures, patient-reported experience measures, and healthcare and societal costs should complement clinical endpoints to capture the total value of the intervention, as has been previously proposed in the IBD population.^8^ Furthermore, increasing the inclusion of compensated patients may be advantageous for RM tools focusing on general well-being and prevention of a first decompensation event, given the adverse prognostic impact of decompensation.^39^^,^^40^ To avoid dilution of observable clinical effects, future studies should consider stratifications or separate evaluations of compensated and decompensated patient populations. Ongoing large RCTs may further clarify the clinical benefits of RM, many of which use RM tools that have been presented in this review.^41^, ^42^, ^43^, ^44^ More importantly, collaboration and consensus among RM developers and hepatology experts are required to address the identified research gaps and optimize the design and impact of future research.

### Strengths and Limitations

This review has several limitations. Studies focusing on self-management, without evaluating disease outcomes, were excluded, which may have led to the omission of promising pilot RM interventions. However, restricting inclusion to RM tools that have been evaluated for clinical outcomes ensured the analysis reflected their impact on clinical care. Study heterogeneity precluded quantitative analysis, including evaluation for publication bias and comparison of RM tools. However, this constitutes a bias inherent to the emerging field, as all starting RM platforms inevitably differ in certain technical or content aspects. Additionally, we highlight that the framework of development and evaluation trajectories of RM studies in liver cirrhosis is a proposal that has not been established within a consensus meeting but could serve as a start for consensus and collaboration.

Strengths include a comprehensive search strategy and a systematic overview of RM interventions across multiple modalities. Importantly, this review provides a pragmatic framework of methodological quality, highlights evidence gaps, and proposes directions to advance the research in the field.

## Conclusion

RM for patients with liver cirrhosis promotes outpatient clinical care and improves hospital readmissions. However, evidence on RM for patients with liver cirrhosis remains fragmented and methodologically limited, with highly variable quality of development processes, precluding firm conclusions on clinical outcomes or comparisons of RM tools. Although feasibility studies show promise, they should naturally be distinguished from and followed by well-powered trials using standardized protocols, including secondary hepatology centers, core outcome sets, established follow-up durations, and matched (external) validation, as proposed in this review. We call for consensus and collaboration among RM and hepatology experts, which are essential to optimize RM development processes and future studies. Meeting these requirements will provide a substantial boost to the research field and enable the successful implementation of RM in patients with liver cirrhosis.

## Declaration of AI and AI-Assisted Technologies in the Writing Process

During the preparation of this work, the authors used ChatGPT in order to assist with language refinement and optimize readability in accordance with American English standards. The authors reviewed and edited the content as needed and take full responsibility for the content of the publication.

## Supplementary Materials

Supplementary Material

Extended PDF
