# Supplementary material for: Quality of Evidence in Remote Monitoring of Patients With Liver Cirrhosis: A Systematic Review
Source: Gastro Hep Adv. 2026 May 8;5(8):100997. doi: 10.1016/j.gastha.2026.100997 (PMC13264051; doi:10.1016/j.gastha.2026.100997)
Supplement: Supplementary Material [file mmc1.pdf]

Supplementary Materials

Title: Quality of evidence and research gaps in remote monitoring of liver cirrhosis patients: a systematic review.

Britt van Ruijven[1,2], Marten A. Lantinga[3], Joost P.H. Drenth[3], Marieke J. Pierik[1,2], Tom J.G. Gevers[1, 2, *], G. Veldhuijzen[4, *]

Table of contents

Pages 2-4	Table S1. PRISMA 2020 Checklist
Pages 5-17:	Table S2. Search strategies
Pages 18:	Table S3. Deduplication strategies
Page 19:	Table S4. Newcastle-Ottawa Scale Quality assessment scores of observational cohort studies
Page 20-21	Table S5. Clinical outcomes
Page 22:	Table S6. RM development quality assessment, according to V3+ framework
Page 23-24:	Material S1. Newcastle-Ottawa Scale Quality assessment scores of observational cohort studies
Page 25:	Figure S1. Cochrane Risk of Bias Assessment Tool of RCT’s (Rob2)
Page 26:	References

Table S1. PRISMA 2020 Checklist

Section and Topic	Item #	Checklist item	Location where item is reported
**TITLE**			
Title	1	Identify the report as a systematic review.	Title
**ABSTRACT**			
Abstract	2	See the PRISMA 2020 for Abstracts checklist.	Abstract
**INTRODUCTION**			
Rationale	3	Describe the rationale for the review in the context of existing knowledge.	Introduction
Objectives	4	Provide an explicit statement of the objective(s) or question(s) the review addresses.	Introduction and Materials and methods
**METHODS**			
Eligibility criteria	5	Specify the inclusion and exclusion criteria for the review and how studies were grouped for the syntheses.	Materials and methods
Information sources	6	Specify all databases, registers, websites, organisations, reference lists and other sources searched or consulted to identify studies. Specify the date when each source was last searched or consulted.	Materials and methods
Search strategy	7	Present the full search strategies for all databases, registers and websites, including any filters and limits used.	Materials and methods
Selection process	8	Specify the methods used to decide whether a study met the inclusion criteria of the review, including how many reviewers screened each record and each report retrieved, whether they worked independently, and if applicable, details of automation tools used in the process.	Materials and methods
Data collection process	9	Specify the methods used to collect data from reports, including how many reviewers collected data from each report, whether they worked independently, any processes for obtaining or confirming data from study investigators, and if applicable, details of automation tools used in the process.	Materials and methods
Data items	10a	List and define all outcomes for which data were sought. Specify whether all results that were compatible with each outcome domain in each study were sought (e.g. for all measures, time points, analyses), and if not, the methods used to decide which results to collect.	Materials and methods
	10b	List and define all other variables for which data were sought (e.g. participant and intervention characteristics, funding sources). Describe any assumptions made about any missing or unclear information.	Materials and

Section and Topic	Item #	Checklist item	Location where item is reported
			methods
Study risk of bias assessment	11	Specify the methods used to assess risk of bias in the included studies, including details of the tool(s) used, how many reviewers assessed each study and whether they worked independently, and if applicable, details of automation tools used in the process.	Materials and methods
Effect measures	12	Specify for each outcome the effect measure(s) (e.g. risk ratio, mean difference) used in the synthesis or presentation of results.	n.a.
Synthesis methods	13a	Describe the processes used to decide which studies were eligible for each synthesis (e.g. tabulating the study intervention characteristics and comparing against the planned groups for each synthesis (item #5)).	n.a.
	13b	Describe any methods required to prepare the data for presentation or synthesis, such as handling of missing summary statistics, or data conversions.	n.a.
	13c	Describe any methods used to tabulate or visually display results of individual studies and syntheses.	Materials and methods
	13d	Describe any methods used to synthesize results and provide a rationale for the choice(s). If meta-analysis was performed, describe the model(s), method(s) to identify the presence and extent of statistical heterogeneity, and software package(s) used.	n.a.
	13e	Describe any methods used to explore possible causes of heterogeneity among study results (e.g. subgroup analysis, meta-regression).	Results
	13f	Describe any sensitivity analyses conducted to assess robustness of the synthesized results.	n.a.
Reporting bias assessment	14	Describe any methods used to assess risk of bias due to missing results in a synthesis (arising from reporting biases).	n.a.
Certainty assessment	15	Describe any methods used to assess certainty (or confidence) in the body of evidence for an outcome.	n.a.
**RESULTS**			
Study selection	16a	Describe the results of the search and selection process, from the number of records identified in the search to the number of studies included in the review, ideally using a flow diagram.	Results
	16b	Cite studies that might appear to meet the inclusion criteria, but which were excluded, and explain why they were excluded.	n.a.
Study characteristics	17	Cite each included study and present its characteristics.	Results
Risk of bias in studies	18	Present assessments of risk of bias for each included study.	Results
Results of individual studies	19	For all outcomes, present, for each study: (a) summary statistics for each group (where appropriate) and (b) an effect estimate and its precision (e.g. confidence/credible interval), ideally using structured tables or plots.	n.a.
Results of syntheses	20a	For each synthesis, briefly summarise the characteristics and risk of bias among contributing studies.	Results
	20b	Present results of all statistical syntheses conducted. If meta-analysis was done, present for each the summary estimate and its precision (e.g.	Results

Section and Topic	Item #	Checklist item	Location where item is reported
		confidence/credible interval) and measures of statistical heterogeneity. If comparing groups, describe the direction of the effect.	
	20c	Present results of all investigations of possible causes of heterogeneity among study results.	Results
	20d	Present results of all sensitivity analyses conducted to assess the robustness of the synthesized results.	Results
Reporting biases	21	Present assessments of risk of bias due to missing results (arising from reporting biases) for each synthesis assessed.	Results
Certainty of evidence	22	Present assessments of certainty (or confidence) in the body of evidence for each outcome assessed.	n.a.
**DISCUSSION**			
Discussion	23a	Provide a general interpretation of the results in the context of other evidence.	Discussion
	23b	Discuss any limitations of the evidence included in the review.	Discussion
	23c	Discuss any limitations of the review processes used.	Discussion
	23d	Discuss implications of the results for practice, policy, and future research.	Discussion
**OTHER INFORMATION**			
Registration and protocol	24a	Provide registration information for the review, including register name and registration number, or state that the review was not registered.	Methods and materials
	24b	Indicate where the review protocol can be accessed, or state that a protocol was not prepared.	Methods and materials
	24c	Describe and explain any amendments to information provided at registration or in the protocol.	n.a.
Support	25	Describe sources of financial or non-financial support for the review, and the role of the funders or sponsors in the review.	Title page
Competing interests	26	Declare any competing interests of review authors.	Title page
Availability of data, code and other materials	27	Report which of the following are publicly available and where they can be found: template data collection forms; data extracted from included studies; data used for all analyses; analytic code; any other materials used in the review.	Title page

From: Page MJ, McKenzie JE, Bossuyt PM, Boutron I, Hoffmann TC, Mulrow CD, et al. The PRISMA 2020 statement: an updated guideline for reporting systematic reviews. BMJ 2021;372:n71. doi: 10.1136/bmj.n71. This work is licensed under CC BY 4.0. To view a copy of this license, visit https://creativecommons.org/licenses/by/4.0/

Table S2. Search strategies

Concept	Search string 29-11-2024 MEDLINE via Ovid	Results
Remote Monitoring	Exp "Computers, Handheld"/ or exp "Digital Health"/ or exp "Mobile applications"/ or exp "Monitoring, ambulatory"/ or exp "Point-of-Care Systems"/ or exp "Rapid Diagnostic Tests"/ or exp "Rapid On-site Evaluation"/ or exp "Telemedicine"/ or "Telemetry"/ or "Videoconferencing"/ or (App or Apps or Desktop or (Digital ADJ3 (care* or consult* or diagnos* or health* or medicine or monitor* or rehab* or therap* or visit*)) or Ehealth* or E-health* or "E-counseling" or "E-Therap*" or (Electronic* ADJ3 (app* or health* or medicine*)) or Emedicine or "E-medicine" or EncephalApp or "Distan* Counsel*" or ((Handheld OR "Hand Held") ADJ3 (computer* or device*)) or "Health app*" or (Home ADJ3 (medicine or monitor*)) or iPad or Laptop or (Medical ADJ3 (app*)) or Mhealth or "M-health*" or (Mobile ADJ3 (app* or computer or device* or health* or medicine* or monitor*)) or "Outpatient Monitor*" or "Patient* Monitor*" or ("Point-of-care" ADJ3 (test* or diagnos*)) or "Rapid Diagnos*" or "Rapid on-site diagnos*" or "Rapid on-site evaluation" or (Remote ADJ3 (care* or consul* or diagnos* or health* or manag* or medicine* or monitor* or rehab*)) or Smartphone* or "smart-phone*" or Smartwatch or Tablet or Telecare* or Teleconsul* or Telediagnos* or Telehealth* or Telemedicine* or Telemonitor* or Telemetr* or "Tele-metr*" or Telerehab* or Telerefer* or Teletherap* or Televisit* or (Tele ADJ3 (care* or consult* or diagnos* or health* or manag* or medicine* or monitor* or rehab* or refer* or therap* or visit*)) or Thealth* or "T-health*" or Videoconsult* or "Video Consult*" or Videoconf* or "Video-conf*" or (Virtual ADJ3 (care* or clinic* or consult* or health* or medicine* or monitor* or rehab* or therap* or visit*)) or Watch).ti,ab,kf.	460495
Cirrhosis	Exp "Hepatic Insufficiency"/ or exp "Hepatopulmonary syndrome"/ or exp "Hepatorenal syndrome"/ or exp "Hypertension, portal"/ or exp "Liver cirrhosis"/ or exp Ascites/ or (Ascites or Cirrh* or ((Chronic*) ADJ3 (liver OR hepatic*)) or CLD or Encephalopath* or (("end stage" or "late stage") ADJ3 (liver* or hepatic*)) or ESLD or (esophag* ADJ3 (varix* or varic*)) or (oesophag* ADJ3 (varix* or varic*)) or (gastric* ADJ3 (varix* or varic*)) or ((liver or hepatic*) ADJ3 (fibrosis or fail* or insufficien* or dysfunction*)) or "hepatopulmonary syndrome*" or "hepato-pulmonary syndrome*" or "hepatorenal syndrome*" or "hepato-renal syndrome*" or "portal hypertension" or (varic* ADJ3 (hemorrhag* or bleed*)) or (varix* ADJ3 (hemorrhag* or bleed*))).ti,ab,kf.	374560
(Remote) Monitoring AND Cirrhosis	1. AND 2.	2440
**Concept**	**Search string 14-07-2025 MEDLINE via Ovid**	**Results**
Remote Monitoring	Identical	490254

Cirrhosis	Identical	386509
(Remote) Monitoring AND Cirrhosis	1. AND 2.	2630
**Concept**	**Search string 12-01-2026 MEDLINE via Ovid**	**Results**
Remote Monitoring	Identical	516107
Cirrhosis	Identical	396961
(Remote) Monitoring AND Cirrhosis	1. AND 2.	2823
**Concept**	**Search string 29-11-2024 Embase via Ovid**	**Results**
Remote Monitoring	exp personal digital assistant/ or exp "Digital Health"/ or exp "Mobile application"/ or exp "ambulatory monitoring"/ or exp "Point-of-Care System"/ or exp "Rapid Test"/ or exp "Rapid On-site Evaluation"/ or exp "Telemedicine"/ or "Telemetry"/ or "Videoconferencing"/ or (App or Apps or Desktop or (Digital ADJ3 (care* or consult* or diagnos* or health* or medicine or monitor* or rehab* or therap* or visit*)) or Ehealth* or E-health* or E-counseling or E-Therap* or (Electronic* ADJ3 (app* or health* or medicine*)) or Emedicine or “E-medicine” or EncephalApp or "Distan* Counsel*" or ((Handheld OR "Hand Held") ADJ3 (computer* or device*)) or “Health app*” or (Home ADJ3 (medicine or monitor*)) or iPad or Laptop or (Medical ADJ3 (app*)) or Mhealth or “M-health*” or (Mobile ADJ3 (app* or computer or device* or health* or medicine* or monitor*)) or "Outpatient Monitor*" or "Patient* Monitor*" or ("Point-of-care" ADJ3 (test* or diagnos*)) or "Rapid Diagnos*" or "Rapid on-site diagnos*" or "Rapid on-site evaluation" or (Remote ADJ3 (care* or consul* or diagnos* or health* or manag* or medicine* or monitor* or rehab*)) or Smartphone* or “smart-phone*” or Smartwatch or Tablet or Telecare* or Teleconsul* or Telediagnos* or Telehealth* or Telemedicine* or Telemonitor* or Telemetr* or “Tele-metr*” or Telerehab* or Telerefer* or Teletherap* or Televisit* or (Tele ADJ3 (care* or consult* or diagnos* or health* or manag* or medicine* or monitor* or rehab* or refer* or therap* or visit*)) or Thealth* or “T-health*” or Videoconsult* or “Video Consult*” or Videoconf* or “Video-conf*” or (Virtual ADJ3 (care* or clinic* or consult* or health* or medicine* or monitor* or rehab* or therap* or visit*)) or Watch).ti,ab,kf.	615390
Cirrhosis	exp "Liver failure"/ or exp "Hepatopulmonary syndrome"/ or exp "Hepatorenal syndrome"/ or exp "Portal hypertension"/ or exp "Liver cirrhosis"/ or exp Ascites/ or (Ascites or Cirrh* or (Chronic* adj3 (liver or hepatic*)) or CLD or Encephalopath* or (("end stage" or "late stage") adj3 (liver* or hepatic*)) or ESLD or (esophag* adj3 (varix* or varic*)) or (oesophag* adj3 (varix* or varic*)) or (gastric* adj3 (varix* or varic*)) or ((liver or hepatic*) adj3 (fibrosis or fail* or insufficien* or dysfunction*)) or "hepatopulmonary	588901

	syndrome*" or "hepato-pulmonary syndrome*" or "hepatorenal syndrome*" or "hepato-renal syndrome*" or "portal hypertension" or (varic* adj3 (hemorrhag* or bleed*)) or (varix* adj3 (hemorrhag* or bleed*))).ti,ab,kf.	
(Remote) Monitoring AND Cirrhosis	1. AND 2.	5944
**Concept**	**Search string 14-07-2025 Embase via Ovid**	**Results**
Remote Monitoring	Identical	689398
Cirrhosis	Identical	618577
(Remote) Monitoring AND Cirrhosis	1. AND 2.	6901
**Concept**	**Search string 13-01-2026 Embase via Ovid**	**Results**
Remote Monitoring	Identical	734899
Cirrhosis	Identical	645992
(Remote) Monitoring AND Cirrhosis	1. AND 2.	7479
**Concept**	**Search string 29-11-2024 Web of Science**	**Results**
Remote Monitoring	TS=(“personal digital assistant” or "Digital Health" or "Mobile application" or "ambulatory monitoring" or "Point-of-Care System" or "Rapid Test" or "Rapid On-site Evaluation" or "Telemedicine" or "Telemetry" or "Videoconferencing" or (App or Apps or Desktop or (Digital NEAR/2 (care* or consult* or diagnos* or health* or medicine or monitor* or rehab* or therap* or visit*)) or Ehealth* or “E-health*” or “E-counseling” or “E-Therap*” or (Electronic* NEAR/2 (app* or health* or medicine*)) or Emedicine or “E-medicine” or EncephalApp or "Distan* Counsel*" or ((Handheld OR "Hand Held") NEAR/2 (computer* or device*)) or “Health app*” or (Home NEAR/2 (medicine or monitor*)) or iPad or Laptop or (Medical NEAR/2 (app*)) or Mhealth or “M-health*” or (Mobile NEAR/2 (app* or computer or device* or health* or medicine* or monitor*)) or "Outpatient Monitor*" or "Patient* Monitor*" or ("Point-of-care" NEAR/2 (test* or diagnos*)) or "Rapid Diagnos*" or "Rapid on-site diagnos*" or "Rapid on-site evaluation" or (Remote NEAR/2 (care* or consul* or diagnos* or health* or manag* or medicine* or monitor* or rehab*)) or Smartphone* or “smart-phone*” or Smartwatch or Tablet or Telecare* or Teleconsul* or Telediagnos* or Telehealth* or Telemedicine* or Telemonitor* or Telemetr* or “Tele-metr*” or Telerehab* or Telerefer* or Teletherap* or Televisit* or (Tele NEAR/2 (care* or consult* or diagnos* or health* or manag* or medicine* or monitor* or rehab* or refer* or therap* or visit*)) or Thealth* or “T-health*” or Videoconsult* or “Video Consult*” or Videoconf* or “Video-conf*”	851415

	or (Virtual NEAR/2 (care* or clinic* or consult* or health* or medicine* or monitor* or rehab* or therap* or visit*)) or Watch))	
Cirrhosis	TS=("Liver failure" or "Hepatopulmonary syndrome" or "Hepatorenal syndrome" or "Portal hypertension" or "Liver cirrhosis" or Ascites or (Ascites or Cirrh* or (Chronic* NEAR/2 (liver or hepatic*)) or CLD or Encephalopath* or (("end stage" or "late stage") NEAR/2 (liver* or hepatic*)) or ESLD or (esophag* NEAR/2 (varix* or varic*)) or (oesophag* NEAR/2 (varix* or varic*)) or (gastric* NEAR/2 (varix* or varic*)) or ((liver or hepatic*) NEAR/2 (fibrosis or fail* or insufficien* or dysfunction*)) or "hepatopulmonary syndrome*" or "hepato-pulmonary syndrome*" or "hepatorenal syndrome*" or "hepato-renal syndrome*" or "portal hypertension" or (varic* NEAR/2 (hemorrhag* or bleed*)) or (varix* NEAR/2 (hemorrhag* or bleed*))))	348725
(Remote) Monitoring AND Cirrhosis	1. AND 2.	2761
**Concept**	**Search string 14-07-2025 Web of Science**	**Results**
Remote Monitoring	Identical	902493
Cirrhosis	Identical	362681
(Remote) Monitoring AND Cirrhosis	1. AND 2.	2966
**Concept**	**Search string 13-01-2026 Web of Science**	**Results**
Remote Monitoring	Identical	943636
Cirrhosis	Identical	373250
(Remote) Monitoring AND Cirrhosis	1. AND 2.	3144
**Concept**	**Search string 29-11-2024 Cochrane Library**	**Results**

Remote Monitoring	(personal NEXT digital NEXT assistant or Digital NEXT Health or Mobile NEXT application or ambulatory NEXT monitoring or Point NEXT of NEXT Care NEXT System or Rapid NEXT Test or Rapid NEXT On NEXT site NEXT Evaluation or Telemedicine or Telemetry or Videoconferencing or (App or Apps or Desktop or (Digital NEAR/3 (care* or consult* or diagnos* or health* or medicine or monitor* or rehab* or therap* or visit*)) or Ehealth* or E NEXT health* or E NEXT counseling or E NEXT Therap* or (Electronic* NEAR/3 (app* or health* or medicine*)) or Emedicine or E NEXT medicine or EncephalApp or Distan* NEXT Counsel* or ((Handheld OR Hand NEXT Held) NEAR/3 (computer* or device*)) or Health NEXT app* or (Home NEAR/3 (medicine or monitor*)) or iPad or Laptop or (Medical NEAR/3 (app*)) or Mhealth or M NEXT health* or (Mobile NEAR/3 (app* or computer or device* or health* or medicine* or monitor*)) or Outpatient NEXT Monitor* or Patient* NEXT Monitor* or (Point NEXT of NEXT care NEAR/3 (test* or diagnos*)) or Rapid NEXT Diagnos* or Rapid NEXT on NEXT site NEXT diagnos* or Rapid NEXT on NEXT site NEXT evaluation or (Remote NEAR/3 (care* or consul* or diagnos* or health* or manag* or medicine* or monitor* or rehab*)) or Smartphone* or smart NEXT phone* or Smartwatch or Tablet or Telecare* or Teleconsul* or Telediagnos* or Telehealth* or Telemedicine* or Telemonitor* or Telemetr* or Tele NEXT metr* or Telerehab* or Telerefer* or Teletherap* or Televisit* or (Tele NEAR/3 (care* or consult* or diagnos* or health* or manag* or medicine* or monitor* or rehab* or refer* or therap* or visit*)) or Thealth* or T NEXT health* or Videoconsult* or Video NEXT Consult* or Videoconf* or Vide NEXT conf* or (Virtual NEAR/3 (care* or clinic* or consult* or health* or medicine* or monitor* or rehab* or therap* or visit*)) or Watch)):ti,ab,kw	102397
Cirrhosis	(Liver NEXT failure or Hepatopulmonary NEXT syndrome or Hepatorenal NEXT syndrome or Portal NEXT hypertension or Liver NEXT cirrhosis or Ascites or (Ascites or Cirrh* or (Chronic* NEAR/3 (liver or hepatic*)) or CLD or Encephalopath* or ((end NEXT stage or late NEXT stage) NEAR/3 (liver* or hepatic*)) or ESLD or (esophag* NEAR/3 (varix* or varic*)) or (oesophag* NEAR/3 (varix* or varic*)) or (gastric* NEAR/3 (varix* or varic*)) or ((liver or hepatic*) NEAR/3 (fibrosis or fail* or insufficien* or dysfunction*)) or hepatopulmonary NEXT syndrome* or hepato-pulmonary NEXT syndrome* or hepatorenal NEXT syndrome* or hepato-renal NEXT syndrome* or portal NEXT hypertension or (varic* NEAR/3 (hemorrhag* or bleed*)) or (varix* NEAR/3 (hemorrhag* or bleed*)))):ti,ab,kw	27729
(Remote) Monitoring AND Cirrhosis	1. AND 2.	1262
**Concept**	**Search string 14-07-2025 Cochrane Library**	**Results**
Remote Monitoring	Identical	137492

Cirrhosis	Identical	28218
(Remote) Monitoring AND Cirrhosis	1. AND 2.	1686
**Concept**	**Search string 14-01-2025 Cochrane Library**	**Results**
Remote Monitoring	Identical	144869
Cirrhosis	Identical	29478
(Remote) Monitoring AND Cirrhosis	1. AND 2.	1778
**Concept**	**Search string 29-11-2024 CINAHL via EBSCO**	**Results**
Remote Monitoring	MH("Computers, Hand-held+" or "Digital Health+" or "Mobile applications+" or "Monitoring, Physiologic+" " or "Clinical Information Systems+" or "Rapid Diagnostic Tests+" or "Rapid On-site Evaluation+" or "Telemedicine+" or "Telemetry" or "Videoconferencing") or TI(App or Apps or Desktop or (Digital N2 (care* or consult* or diagnos* or health* or medicine or monitor* or rehab* or therap* or visit*)) or Ehealth* or "E-health*" or "E-counseling" or "E-Therap*" or (Electronic* N2 (app* or health* or medicine*)) or Emedicine or "E-medicine" or EncephalApp or "Distan* Counsel*" or ((Handheld OR "Hand Held") N2 (computer* or device*)) or "Health app*" or (Home N2 (medicine or monitor*)) or iPad or Laptop or (Medical N2 (app*)) or Mhealth or "M-health*" or (Mobile N2 (app* or computer or device* or health* or medicine* or monitor*)) or "Outpatient Monitor*" or "Patient* Monitor*" or ("Point-of-care" N2 (test* or diagnos*)) or "Rapid Diagnos*" or "Rapid on-site diagnos*" or "Rapid on-site evaluation" or (Remote N2 (care* or consul* or diagnos* or health* or manag* or medicine* or monitor* or rehab*)) or Smartphone* or "smart-phone*" or Smartwatch or Tablet or Telecare* or Teleconsul* or Telediagnos* or Telehealth* or Telemedicine* or Telemonitor* or Telemetr* or "Tele-metr*" or Telerehab* or Telerefer* or Teletherap* or Televisit* or (Tele N2 (care* or consult* or diagnos* or health* or manag* or medicine* or monitor* or rehab* or refer* or therap* or visit*)) or Thealth* or "T-health*" or Videoconsult* or "Video Consult*" or Videoconf* or "Video-conf*" or (Virtual N2 (care* or clinic* or consult* or health* or medicine* or monitor* or rehab* or therap* or visit*)) or Watch) or AB(App or Apps or Desktop or (Digital N2 (care* or consult* or diagnos* or health* or medicine or monitor* or rehab* or therap* or visit*)) or Ehealth* or "E-health*" or "E-counseling" or "E-Therap*" or (Electronic* N2 (app* or health* or medicine*)) or Emedicine or "E-medicine" or EncephalApp or "Distan* Counsel*" or ((Handheld OR "Hand Held") N2 (computer* or device*)) or "Health app*" or (Home N2 (medicine or monitor*)) or iPad or Laptop or (Medical N2 (app*)) or	50652

	Mhealth or "M-health*" or (Mobile N2 (app* or computer or device* or health* or medicine* or monitor*)) or "Outpatient Monitor*" or "Patient* Monitor*" or ("Point-of-care" N2 (test* or diagnos*)) or "Rapid Diagnos*" or "Rapid on-site diagnos*" or "Rapid on-site evaluation" or (Remote N2 (care* or consul* or diagnos* or health* or manag* or medicine* or monitor* or rehab*)) or Smartphone* or "smart-phone*" or Smartwatch or Tablet or Telecare* or Teleconsul* or Telediagnos* or Telehealth* or Telemedicine* or Telemonitor* or Telemetr* or "Tele-metr*" or Telerehab* or Telerefer* or Teletherap* or Televisit* or (Tele N2 (care* or consult* or diagnos* or health* or manag* or medicine* or monitor* or rehab* or refer* or therap* or visit*)) or Thealth* or "T-health*" or Videoconsult* or "Video Consult*" or Videoconf* or "Video-conf*" or (Virtual N2 (care* or clinic* or consult* or health* or medicine* or monitor* or rehab* or therap* or visit*)) or Watch)	
Cirrhosis	(MH ("Liver Diseases+" OR "Hepatopulmonary syndrome+" or "Hepatorenal syndrome+" or "Hypertension, portal+" or "Liver cirrhosis+" or Ascites+) or TI(Ascites or Cirrh* or (Chronic* N2 (liver or hepatic*)) or CLD or Encephalopath* or (("end stage" or "late stage") N2 (liver* or hepatic*)) or ESLD or (esophag* N2 (varix* or varic*)) or (oesophag* N2 (varix* or varic*)) or (gastric* N2 (varix* or varic*)) or ((liver or hepatic*) N2 (fibrosis or fail* or insufficien* or dysfunction*)) or "hepatopulmonary syndrome*" or "hepato-pulmonary syndrome*" or "hepatorenal syndrome*" or "hepato-renal syndrome*" or "portal hypertension" or (varic* N2 (hemorrhag* or bleed*)) or (varix* N2 (hemorrhag* or bleed*))) OR AB (Ascites or Cirrh* or (Chronic* N2 (liver or hepatic*)) or CLD or Encephalopath* or (("end stage" or "late stage") N2 (liver* or hepatic*)) or ESLD or (esophag* N2 (varix* or varic*)) or (oesophag* N2 (varix* or varic*)) or (gastric* N2 (varix* or varic*)) or ((liver or hepatic*) N2 (fibrosis or fail* or insufficien* or dysfunction*)) or "hepatopulmonary syndrome*" or "hepato-pulmonary syndrome*" or "hepatorenal syndrome*" or "hepato-renal syndrome*" or "portal hypertension" or (varic* N2 (hemorrhag* or bleed*)) or (varix* N2 (hemorrhag* or bleed*))))	108026
(Remote) Monitoring AND Cirrhosis	1. AND 2.	239
**Concept**	**Search string 14-07-2025 CINAHL via EBSCO**	**Results**
Remote Monitoring	Identical	55277
Cirrhosis	Identical	109447
(Remote) Monitoring AND Cirrhosis	1. AND 2.	274
**Concept**	**Search string 13-01-2026 CINAHL via EBSCO**	**Results**
Remote Monitoring	Identical	61181

Cirrhosis	Identical	111871
(Remote) Monitoring AND Cirrhosis	1. AND 2.	309
**Concept**	**Search string 29-11-2024 Epistemonikos**	**Results**
Remote Monitoring	title:(App or Apps or Desktop or (Digital AND (care* or consult* or diagnos* or health* or medicine or monitor* or rehab* or therap* or visit*)) or Ehealth* or “E-health*” or “E-counseling” or “E-Therap*” or (Electronic* AND (app* or health* or medicine*)) or Emedicine or “E-medicine” or EncephalApp or "Distan* Counsel*" or ((Handheld OR "Hand Held") AND (computer* or device*)) or “Health app*” or (Home AND (medicine or monitor*)) or iPad or Laptop or (Medical AND (app*)) or Mhealth or “M-health*” or (Mobile AND (app* or computer or device* or health* or medicine* or monitor*)) or "Outpatient Monitor*" or "Patient* Monitor*" or ("Point-of-care" AND (test* or diagnos*)) or "Rapid Diagnos*" or "Rapid on-site diagnos*" or "Rapid on-site evaluation" or (Remote AND (care* or consul* or diagnos* or health* or manag* or medicine* or monitor* or rehab*)) or Smartphone* or “smart-phone*” or Smartwatch or Tablet or Telecare* or Teleconsul* or Telediagnos* or Telehealth* or Telemedicine* or Telemonitor* or Telemetr* or “Tele-metr*” or Telerehab* or Telerefer* or Teletherap* or Televisit* or (Tele AND (care* or consult* or diagnos* or health* or manag* or medicine* or monitor* or rehab* or refer* or therap* or visit*)) or Thealth* or “T-health*” or Videoconsult* or “Video Consult*” or Videoconf* or “Video-conf*” or (Virtual AND (care* or clinic* or consult* or health* or medicine* or monitor* or rehab* or therap* or visit*)) or Watch) or abstract:(App or Apps or Desktop or (Digital AND (care* or consult* or diagnos* or health* or medicine or monitor* or rehab* or therap* or visit*)) or Ehealth* or “E-health*” or “E-counseling” or “E-Therap*” or (Electronic* AND (app* or health* or medicine*)) or Emedicine or “E-medicine” or EncephalApp or "Distan* Counsel*" or ((Handheld OR "Hand Held") AND (computer* or device*)) or “Health app*” or (Home AND (medicine or monitor*)) or iPad or Laptop or (Medical AND (app*)) or Mhealth or “M-health*” or (Mobile AND (app* or computer or device* or health* or medicine* or monitor*)) or "Outpatient Monitor*" or "Patient* Monitor*" or ("Point-of-care" AND (test* or diagnos*)) or "Rapid Diagnos*" or "Rapid on-site diagnos*" or "Rapid on-site evaluation" or (Remote AND (care* or consul* or diagnos* or health* or manag* or medicine* or monitor* or rehab*)) or Smartphone* or “smart-phone*” or Smartwatch or Tablet or Telecare* or Teleconsul* or Telediagnos* or Telehealth* or Telemedicine* or Telemonitor* or Telemetr* or “Tele-metr*” or Telerehab* or Telerefer* or Teletherap* or Televisit* or (Tele AND (care* or consult* or diagnos* or health* or manag* or medicine* or monitor* or rehab* or refer* or therap* or visit*)) or Thealth* or “T-health*” or Videoconsult* or “Video Consult*” or Videoconf* or	318819

	“Video-conf*” or (Virtual AND (care* or clinic* or consult* or health* or medicine* or monitor* or rehab* or therap* or visit*)) or Watch)	
Cirrhosis	title:(Ascites or Cirrh* or (Chronic* AND (liver or hepatic*)) or CLD or Encephalopath* or (("end stage" or "late stage") AND (liver* or hepatic*)) or ESLD or (esophag* AND (varix* or varic*)) or (oesophag* AND (varix* or varic*)) or (gastric* AND (varix* or varic*)) or ((liver or hepatic*) AND (fibrosis or fail* or insufficien* or dysfunction*)) or "hepatopulmonary syndrome*" or "hepato-pulmonary syndrome*" or "hepatorenal syndrome*" or "hepato-renal syndrome*" or "portal hypertension" or (varic* AND (hemorrhag* or bleed*)) or (varix* AND (hemorrhag* or bleed*))) OR abstract:(Ascites or Cirrh* or (Chronic* AND (liver or hepatic*)) or CLD or Encephalopath* or (("end stage" or "late stage") AND (liver* or hepatic*)) or ESLD or (esophag* AND (varix* or varic*)) or (oesophag* AND (varix* or varic*)) or (gastric* AND (varix* or varic*)) or ((liver or hepatic*) AND (fibrosis or fail* or insufficien* or dysfunction*)) or "hepatopulmonary syndrome*" or "hepato-pulmonary syndrome*" or "hepatorenal syndrome*" or "hepato-renal syndrome*" or "portal hypertension" or (varic* AND (hemorrhag* or bleed*)) or (varix* AND (hemorrhag* or bleed*)))	111096
(Remote) Monitoring AND Cirrhosis	1. AND 2.	4217
**Concept**	**Search string 16-07-2025 Epistemonikos**	**Results**
Remote Monitoring	Identical	399366
Cirrhosis	Identical	132451
(Remote) Monitoring AND Cirrhosis	1. AND 2.	5839
**Concept**	**Search string 14-01-2026 Epistemonikos**	**Results**
Remote Monitoring	Identical	425826
Cirrhosis	Identical	137242
(Remote) Monitoring AND Cirrhosis	1. AND 2.	6112
**Concept**	**Search string 29-11-2024 PROSPERO**	**Results**

Remote Monitoring	(personal digital assistant OR Digital Health OR Mobile application OR ambulatory monitoring OR Point of Care System OR Rapid Test OR Rapid On site Evaluation OR Telemedicine OR Telemetry OR Videoconferencing OR App OR Apps OR Desktop OR (Digital NEAR2 (care* OR consult* OR diagnos* OR health* OR medicine OR monitOR* OR rehab* OR therap* OR visit*)) OR Ehealth* OR E health* OR E counseling OR E Therap* OR (Electronic* NEAR2 (app* OR health* OR medicine*)) OR Emedicine OR E medicine OR EncephalApp OR Distan* Counsel* OR ((Handheld OR Hand Held) NEAR2 (computer* OR device*)) OR Health app* OR (Home NEAR2 (medicine OR monitOR*)) OR iPad OR Laptop OR (Medical NEAR2 (app*)) OR Mhealth OR M health* OR (Mobile NEAR2 (app* OR computer OR device* OR health* OR medicine* OR monitOR*)) OR Outpatient MonitOR* OR Patient* MonitOR* OR (Point of care NEAR2 (test* OR diagnos*)) OR Rapid Diagnos* OR Rapid on site diagnos* OR Rapid on site evaluation OR (Remote NEAR2 (care* OR consul* OR diagnos* OR health* OR manag* OR medicine* OR monitOR* OR rehab*))) OR<br>(Smartphone* OR smart phone* OR Smartwatch OR Tablet OR Telecare* OR Teleconsul* OR Telediagnos* OR Telehealth* OR Telemedicine* OR TelemonitOR* OR Telemetr* OR Tele metr* OR Telerehab* OR Telerefer* OR Teletherap* OR Televisit* OR (Tele NEAR2 (care* OR consult* OR diagnos* OR health* OR manag* OR medicine* OR monitOR* OR rehab* OR refer* OR therap* OR visit*)) OR Thealth* OR T health* OR Videoconsult* OR Video Consult* OR Videoconf* OR Video conf* OR (Virtual NEAR2 (care* OR clinic* OR consult* OR health* OR medicine* OR monitOR* OR rehab* OR therap* OR visit*)) OR Watch)<br><br>1 OR 2	(15850)<br>(9445)<br><br>1 OR 2 →<br><br>20694
Cirrhosis	(Liver failure OR Hepatopulmonary syndrome OR Hepatorenal syndrome OR Portal hypertension OR Liver cirrhosis OR Ascites OR (Ascites OR Cirrh* OR (Chronic* NEAR2 (liver OR hepatic*)) OR CLD OR Encephalopath* OR ((end stage OR late stage) NEAR2 (liver* OR hepatic*)) OR ESLD OR (esophag* NEAR2 (varix* OR varic*)) OR (oesophag* NEAR2 (varix* OR varic*)) OR (gastric* NEAR2 (varix* OR varic*)) OR ((liver OR hepatic*) NEAR2 (fibrosis OR fail* OR insufficien* OR dysfunction*)) OR hepatopulmonary syndrome* OR hepato pulmonary syndrome* OR hepatorenal syndrome* OR hepato renal syndrome* OR portal hypertension OR (varic* NEAR2 (hemorrhag* OR bleed*)) OR (varix* NEAR2 (hemorrhag* OR bleed*))))	5858
(Remote) Monitoring AND Cirrhosis	((1 OR 2) AND 3)	329
**Concept**	**Search string 14-07-2025 PROSPERO**	**Results**
Remote Monitoring	(personal digital assistant OR Digital Health OR Mobile application OR ambulatory monitoring OR Point of Care System OR Rapid	(14962)<br>(11180)

	Test OR Rapid On site Evaluation OR Telemedicine OR Telemetry OR Videoconferencing OR App OR Apps OR Desktop OR (Digital NEAR2 (care* OR consult* OR diagnos* OR health* OR medicine OR monitOR* OR rehab* OR therap* OR visit*)) OR Ehealth* OR E health* OR E counseling OR E Therap* OR (Electronic* NEAR2 (app* OR health* OR medicine*)) OR Emedicine OR E medicine OR EncephalApp OR Distan* Counsel* OR **((Handheld OR Held) NEAR2 (computer* OR device*))** OR Health app* OR (Home NEAR2 (medicine OR monitOR*)) OR iPad OR Laptop OR (Medical NEAR2 (app*)) OR Mhealth OR M health* OR (Mobile NEAR2 (app* OR computer OR device* OR health* OR medicine* OR monitOR*)) OR Outpatient MonitOR* OR Patient* MonitOR* OR (Point of care NEAR2 (test* OR diagnos*)) OR Rapid Diagnos* OR Rapid on site diagnos* OR Rapid on site evaluation OR (Remote NEAR2 (care* OR consul* OR diagnos* OR health* OR manag* OR medicine* OR monitOR* OR rehab*))) OR<br>(Smartphone* OR smart phone* OR Smartwatch OR Tablet OR Telecare* OR Teleconsul* OR Telediagnos* OR Telehealth* OR Telemedicine* OR TelemonitOR* OR Telemetr* OR Tele metr* OR Telerehab* OR Telerefer* OR Teletherap* OR Televisit* OR (Tele NEAR2 (care* OR consult* OR diagnos* OR health* OR manag* OR medicine* OR monitOR* OR rehab* OR refer* OR therap* OR visit*)) OR Thealth* OR T health* OR Videoconsult* OR Video Consult* OR Videoconf* OR Video conf* OR (Virtual NEAR2 (care* OR clinic* OR consult* OR health* OR medicine* OR monitOR* OR rehab* OR therap* OR visit*)) OR Watch)<br><br>1 OR 2	1 OR 2 →<br><br>20095
Cirrhosis	(Liver failure OR Hepatopulmonary syndrome OR Hepatorenal syndrome OR Portal hypertension OR Liver cirrhosis OR Ascites OR (Ascites OR Cirrh* OR (Chronic* NEAR2 (liver OR hepatic*)) OR CLD OR Encephalopath* OR **((end OR late) NEAR3** (liver* OR hepatic*)) OR ESLD OR (esophag* NEAR2 (varix* OR varic*)) OR (oesophag* NEAR2 (varix* OR varic*)) OR (gastric* NEAR2 (varix* OR varic*)) OR ((liver OR hepatic*) NEAR2 (fibrosis OR fail* OR insufficien* OR dysfunction*)) OR hepatopulmonary syndrome* OR hepato pulmonary syndrome* OR hepatorenal syndrome* OR hepato renal syndrome* OR portal hypertension OR (varic* NEAR2 (hemorrhag* OR bleed*)) OR (varix* NEAR2 (hemorrhag* OR bleed*))))	6399
(Remote) Monitoring AND Cirrhosis	((1 OR 2) AND 3)	210
**Concept**	**Search string 13-01-2026 PROSPERO**	**Results**
Remote Monitoring	Identical to 14-07-2025<br><br>1 OR 2	(18128)<br>(13300)<br><br>1 OR 2 →

		24021
Cirrhosis	Identical to 14-07-2025	7473
(Remote) Monitoring AND Cirrhosis	((1 OR 2) AND 3)	239
**Concept**	**Search string 29-11-2024 ClinicalTrials.gov**	**Results**
Remote Monitoring<br><br>Intervention	(“personal digital assistant” OR "Digital Health" OR "Mobile application" OR "ambulatory monitoring" OR "Point-of-Care System" OR "Rapid Test" OR "Rapid On-site Evaluation" OR "Telemedicine" OR "Telemetry" OR "Videoconferencing" OR (App OR Apps OR Desktop OR (Digital AND (care* OR consult* OR diagnos* OR health* OR medicine OR monitor* OR rehab* OR therap* OR visit*)) OR Ehealth* OR E-health* OR E-counseling OR E-Therap* OR (Electronic* AND (app* OR health* OR medicine*)) OR Emedicine OR E-medicine OR EncephalApp OR "Distan* Counsel*" OR ((Handheld OR "Hand Held") AND (computer* OR device*)) OR “Health app*” OR (Home AND (medicine OR monitor*)) OR iPad OR Laptop OR (Medical AND (app*)) OR Mhealth OR M-health* OR (Mobile AND (app* OR computer OR device* OR health* OR medicine* OR monitor*)) OR "Outpatient Monitor*" OR "Patient* Monitor*" OR ("Point-of-care" AND (test* OR diagnos*)) OR "Rapid Diagnos*" OR "Rapid on-site diagnos*" OR "Rapid on-site evaluation" OR (Remote AND (care* OR consul* OR diagnos* OR health* OR manag* OR medicine* OR monitor* OR rehab*)) OR Smartphone* OR smart-phone* OR Smartwatch OR Tablet OR Telecare* OR Teleconsul* OR Telediagnos* OR Telehealth* OR Telemedicine* OR Telemonitor* OR Telemetr* OR Tele-metr* OR Telerehab* OR Telerefer* OR Teletherap* OR Televisit* OR (Tele AND (care* OR consult* OR diagnos* OR health* OR manag* OR medicine* OR monitor* OR rehab* OR refer* OR therap* OR visit*)) OR Thealth* OR T-health* OR Videoconsult* OR “Video Consult*” OR Videoconf* OR Video-conf* OR (Virtual AND (care* OR clinic* OR consult* OR health* OR medicine* OR monitor* OR rehab* OR therap* OR visit*)) OR Watch))	64035
Cirrhosis<br><br>Condition	("Liver failure" OR "Hepatopulmonary syndrome" OR "Hepatorenal syndrome" OR "Portal hypertension" OR "Liver cirrhosis" OR Ascites OR (Ascites OR Cirrh* OR (Chronic* AND (liver OR hepatic*)) OR CLD OR Encephalopath* OR (("end stage" OR "late stage") AND (liver* OR hepatic*)) OR ESLD OR (esophag* AND (varix* OR varic*)) OR (oesophag* AND (varix* OR varic*)) OR (gastric* AND (varix* OR varic*)) OR ((liver OR hepatic*) AND (fibrosis OR fail* OR insufficien* OR dysfunction*)) OR "hepatopulmonary syndrome*" OR "hepato-pulmonary syndrome*" OR "hepatorenal syndrome*" OR "hepato-renal syndrome*" OR "portal hypertension" OR (varic* AND (hemorrhag* OR bleed*)) OR (varix* AND (hemorrhag* OR bleed*))))	6463

(Remote) Monitoring AND Cirrhosis	1. AND 2.	1173
**Concept**	**Search string 14-07-2025 ClinicalTrials.gov**	**Results**
Remote Monitoring<br><br>Intervention	Identical	67875
Cirrhosis<br><br>Condition	Identical	6753
(Remote) Monitoring AND Cirrhosis	1. AND 2.	1219
**Concept**	**Search string 13-01-2026 ClinicalTrials.gov**	**Results**
Remote Monitoring<br><br>Intervention	Identical	70473
Cirrhosis<br><br>Condition	Identical	6867
(Remote) Monitoring AND Cirrhosis	1. AND 2.	1182

Table S3. Deduplication strategies

1. Deduplication via Endnote with manual revision of marked duplicates.	Step 1 duplicates removed, selection on:<br>Author, year, title, secondary title (journal), DOI, reference type
	Step 2 duplicates removed, selection on:<br>Year, title, reference type
	Step 3 duplicates removed, selection on:<br>Title
	Step 4 duplicates removed, selection on:<br>Year, author
2. Deduplication via DedupEndnote with manual revision of marked duplicates.	
3. Deduplication via Covidence, manual throughout the screening process.	

Table S4. Newcastle-Ottawa Scale Quality assessment scores of observational cohort studies

Design	Year	Author	Selection Q1	Selection Q2	Selection Q3	Selection Q4	Comparability Q5	Outcome Q6	Outcome Q7	Outcome Q8	Total score (0 – 9)	Overall quality
**Prospective cohort with control group**	2. 2023	Kazankov *et al.* (1)	1	1	1	1	1	1	0	1	7	Good
	3. 2024	Penrice *et al.* (2)	1	1	1	1	2	1	0	0	7	Good
	4. 2017	Khungar *et al.* (3) Abstract	1	1	1	1	2	1	0	0	7	Fair
	5. 2024	Ballesteros *et al.* (4) Abstract	1	1	1	1	1	1	0	0	6	Good
**Prospective cohort without control group**	6. 2022	Lin *et al.* (5)	1	0	1	1	0	1	1	1	6	Fair
	7. 2015	Thomson *et al.* (6)	1	0	1	1	0	1	1	1	6	Fair
	8. 2025	Ngu *et al.* (7)	1	0	1	1	0	1	0	1	5	Fair
	9. 2020	Bloom *et al.* (8)	1	0	1	1	0	1	1	0	5	Fair
	10. 2021	Qian *et al.* (9) Abstract	1	0	1	1	0	1	0	0	4	Fair
	11. 2018	Verma *et al.* (10) Abstract	1	0	1	1	0	1	0	0	4	Fair
	12. 2017	Ganapathy *et al.* (11)	1	0	1	1	0	1	0	1	5	Fair

Table S5. Clinical outcomes

Author	Clinical outcomes	Results (controls vs. intervention group)	Significance
1. Shaw, et al. (12)	N avoidable readmissions SOC vs. HIT (%)	19.8% vs. 10%	**p = 0.042**
	OR avoidable readmissions SOC vs HIT	2.14 (95% CI 1.01–4.54)	
	N all cause admissions SOC vs. HIT (%)	48% vs. 30%	**p = 0.005**
	Time to readmission SOC vs. HIT (days)	7.9 vs. 4.7	**p = 0.003**
	N urgent clinic visits arranged after initial engagement with the app	35 patients	
2. Kazankov, et al. (1)	N readmissions (%)	8 (40) vs. 5 (25)	
	N patients with readmissions ≥14 days (%)	4 (20) vs. 0	
	Total number of readmissions	13 vs. 8	
	Time to first readmission (days)	4 (20) vs. 0	
	ITU length of stay (days)	16 vs. 5	
	N unplanned LVP (%)	6 (30) vs. 1 (5)	
	N liver transplantation (%)	0 vs. 1 (5)	
	N deceased (%)	2 (10) vs. 1 (5)	
3. Penrice, et al. (2)	% 30-day readmissions	28 vs. 20 (OR: 0.46 [0.18, 1.97])	p = 0.29
	% 90-day readmissions	47 vs. 34 (OR: 0.59 [0.18, 1.93])	p = 0.38
	% liver-related 90-day readmissions	86 vs. 79	p = 0.63
	% HE and fluid imbalance readmissions	57 vs. 36	p = 0.27
	Median time to first readmission	18 vs. 35 days	p = 0.30
	Mean total number of hospitalized days in 90 days following index admission	4.9 vs. 3.4	p = 0.067
	% ≥2 or more readmissions	36.5 vs. 12	
	90-day mortality	7.7 vs. 2.4	
	1-year mortality	28 vs. 22 (HR 0.62 [0.20, 1.90])	
4. Khungar, et al. (3) Abstract	% 30-day readmissions	21 vs. 15.8	
	30-day potentially preventable readmissions	Not reported, not statistically significant	
	90-day potentially preventable readmissions due to HE and volume overload	33.8 vs 0	**p = 0.02**
5. Ballesteros, et al. (4) Abstract	% Emergency admissions	28 vs. 12	**p<0.001**
	% 30-day decompensation rate	87 vs. 58	**p<0.05**
	% 90-day decompensation rate	72 vs. 47	**p<0.05**
	% 90-day recompensation rate	6 vs. 26	**p<0.05**
	% LVP	15 vs. 65	**p<0.001**
	% 90-day liver-related deaths	10 vs. 5	**p<0.05**
6. Lin, et al. (5)	N readmissions (%)	64 (55.2)	
	N deceased (%)	17 (14.7)	
7. Thomson, et al. (6)	N readmissions (%)	49 (62)	
	Time to first hospital admission (days), censoring death or transplantation	534	

	Hospitalization rate (admissions/follow-up)	1/5.8 months	
	N deceased (%)	20 (25)	
8. Ngu, *et al.* (7)	N 30-day readmissions (%)	9 (15)	
	N 90-day readmissions (%)	16 (27)	
	N 30-day deceased (%)	2 (3)	
	N 90-day deceased (%)	2 (3)	
9. Bloom, et al. (8)	N readmissions (%)	17 (68)	
	N LVP	13	
10. Qian, et al. (9) Abstract	N readmissions (%)	3 (6.25)	
	Clinic visits/patient pre vs. post enrollment	2.4 vs. 1.9	
	Hospitalizations/patient pre vs. post enrollment	0.5 vs. 0.3	
11. Verma, et al. (10) Abstract	N scheduled visits due to system flags (%)	13 (2.5)	
	N emergency visits due to system flags (%)	2 (0.4)	
	N planned hospital admissions due to system flags (%)	20 (3.9)	
12. Ganapathy, et al. (11)	N 30-day readmissions (%)	17 (42.5)	

HIT: Health Information Technology, ITU: Intensive Therapy Unit, LVP: Large Volume Paracentesis, SOC: Standard Of Care

Table S6. RM development quality assessment, according to V3+ framework

Year		Author	Verification	Usability validation	Analytical validation	Clinical validation
			Answers the verification question: Are the sensor data accurate, precise, uniform, and consistent?	Answers the usability validation question: Is the tool easy, efficient, and satisfying to use?	Answers the analytical validation question: Does the algorithm capture the outcome it claims to measure?	Answers the clinical validation question: Are the data informative to answer the specific clinical question in the intended context of use?*
1.	2025	Shaw, et al. (12)	1	1	1	1
2.	2023	Kazankov, et al. (1)	1	1	1	1
3.	2024	Penrice, et al. (2)	NR	1	1	1
4.	2017	Khungar, et al. (3) Abstract	NR	NR	NR	1
5.	2024	Ballesteros, et al. (4) Abstract	1	NR	NR	1
6.	2022	Lin, et al. (5)	1	NR	1	1
7.	2015	Thomson, et al. (6)	NR	1	NR	1
8.	2025	Ngu, *et al.* (7)	NR	1	NR	1
9.	2020	Bloom, et al. (8)	1	1	1	1
10.	2021	Qian, et al. (9) Abstract	NR	NR	NR	1
11.	2018	Verma, et al. (10) Abstract	NR	1	1	1
12.	2017	Ganapathy, et al. (11)	1	1	1	1
**Total**			6	8	7	12

*Measurement of at least one clinical outcome was an inclusion criterium of this systematic review.

Material S1. Newcastle-Ottawa Scale Quality assessment scores of observational cohort studies (13)

NEWCASTLE - OTTAWA QUALITY ASSESSMENT SCALE
COHORT STUDIES

Note: A study can be awarded a maximum of one star for each numbered item within the Selection and Outcome categories. A maximum of two stars can be given for Comparability

Selection

1) Representativeness of the exposed cohort
   a) truly representative of the average **liver cirrhosis patient (compensated or decompensated)** (describe) in the community ❋
   b) somewhat representative of the average **liver cirrhosis patient (compensated or decompensated)** in the community ❋
   c) selected group of users eg nurses, volunteers
   d) no description of the derivation of the cohort

2) Selection of the non exposed cohort
   a) drawn from the same community as the exposed cohort ❋
   b) drawn from a different source
   c) no description of the derivation of the non exposed cohort

3) Ascertainment of exposure
   a) secure record (eg surgical records) ❋
   b) structured interview ❋
   c) written self report
   d) no description

4) Demonstration that outcome of interest was not present at start of study
   a) yes ❋
   b) no

Comparability

1) Comparability of cohorts on the basis of the design or analysis
   a) study controls for (select the most important factor): ❋
   Clinically compensated or decompensated state, according to Baveno VII classification.
   b) study controls for any additional factor ❋ (This criteria could be modified to indicate specific control for a second important factor.)
   MELD-score, Child-Pugh score, age, sex, liver cirrhosis etiology, socioeconomic status surrogates such as education, income, zip code

Outcome

1) Assessment of outcome
   a) independent blind assessment ❋
   b) record linkage ❋
   c) self report
   d) no description

2) Was follow-up long enough for outcomes to occur
   a) yes (select an adequate follow up period for outcome of interest) ❋
   Adequate follow-up period per outcome of interest for the specific study:
   - **Hospital readmission: 3 months**

- **Feasibility: 6 months**
- **Clinical outcome decompensated outpatients: 6 months**
- **Clinical outcomes compensated outpatients: 12 months**

b) no

3) Adequacy of follow up of cohorts

a) complete follow up - all subjects accounted for ❋

b) subjects lost to follow up unlikely to introduce bias - small number lost - > **≥75%** (select an adequate %) follow up, or description provided of those lost) ❋

c) follow up rate **<75%** (select an adequate %) and no description of those lost

d) no statement

Figure S1. Cochrane Risk of Bias Assessment Tool of RCT's (Rob2) (14)

Year	Author	D1	D2	D3	D4	D5	Overall
1. 2025	Shaw *et al. (12)*	+	+	+	+	+	+

+	Low risk
!	Some concerns
-	High risk
D1	Randomisation process
D2	Deviations from the intended interventions
D3	Missing outcome data
D4	Measurement of the outcome
D5	Selection of the reported result

References

1. Kazankov K, Novelli S, Chatterjee DA, Phillips A, Balaji A, Raja M, et al. Evaluation of CirrhoCare R - a digital health solution for home management of individuals with cirrhosis. Journal of hepatology. 2023;78(1):123-32.
2. Penrice DD, Hara KS, Sordi-Chara B, Kezer C, Schmidt K, Kassmeyer B, et al. Design, implementation, and impact of a cirrhosis-specific remote patient monitoring program. Hepatology communications. 2024;8(8).
3. Khungar V, Serper M, Peyton D, Mehta S, Norris A, Huffenberger A, et al. Use of an Innovative Telehealth Platform to Reduce Readmissions and Enable Patient-Centered Care in Cirrhotic Patients. HEPATOLOGY. 2017;66:94A-5A.
4. Ballesteros K, Kumaravel Kanagavelu AS, Crone J, Rahman R, Schlitzer A, Sharma V. P126 Improving patient outcomes in decompensated liver disease through remote monitoring: a real-world experience. Gut. 2024;73(Suppl 3):A90.
5. Lin F-P, Bloomer PM, Grubbs RK, Rockette-Wagner B, Tevar AD, Dunn MA, et al. Low Daily Step Count Is Associated With a High Risk of Hospital Admission and Death in Community-Dwelling Patients With Cirrhosis. Clinical gastroenterology and hepatology : the official clinical practice journal of the American Gastroenterological Association. 2022;20(8):1813-20.e2.
6. Thomson M, Volk M, Kim HM, Piette JD. An Automated Telephone Monitoring System to Identify Patients with Cirrhosis at Risk of Re-hospitalization. Digestive diseases and sciences. 2015;60(12):3563-9.
7. Ngu NLY, Saxby E, Worland T, Anderson P, Stothers L, Hunter J, et al. A Nonrandomized Pilot Study to Investigate the Acceptability and Feasibility of LivR Well: A Multifaceted 28-Day Home-Based Liver Optimization Program for Acute-on-Chronic Liver Failure. Gastro Hep Advances. 2025;4(2):100567.
8. Bloom P, Wang T, Marx M, Tagerman M, Green B, Arvind A, et al. A Smartphone App to Manage Cirrhotic Ascites Among Outpatients: Feasibility Study. JMIR Med Inform. 2020;8(9):e17770.
9. Qian LA, Higgins A, Zapatka S, Taddei TH, Garcia-Tsao G, Jakab SS. Development and feasibility of a disease management program for patients with cirrhosis at the veterans health administration (vha). Hepatology. 2021;74(SUPPL 1):375A.
10. Verma M, Kalman R, Walter JW, Gallagher M, Navarro V. FEASIBILITY OF TELEMONITORING OF SYMPTOMS AND COGNITIVE FUNCTION IN END STAGE LIVER DISEASE. GASTROENTEROLOGY. 2018;154(6):S1221-S2.
11. Ganapathy D, Acharya C, Lachar J, Patidar K, Sterling RK, White MB, et al. The patient buddy app can potentially prevent hepatic encephalopathy-related readmissions. Liver international : official journal of the International Association for the Study of the Liver. 2017;37(12):1843-51.
12. Shaw J, Acharya C, Fagan A, Olofson A, Irwin K, Kolhekar S, et al. Health information technology interventions reduce avoidable readmissions in cirrhosis: The HEROIC randomized controlled trial. Hepatology. 2025.
13. Wells G, Shea B, O'Connell D, Peterson J, Welch V, Losos M, et al. The Newcastle-Ottawa Scale (NOS) for assessing the quality of nonrandomised studies in meta-analyses [Available from: https://www.ohri.ca/programs/clinical_epidemiology/oxford.asp.
14. Sterne JAC, Savovic J, Page MJ, Elbers RG, Blencowe NS, Boutron I, et al. RoB 2: a revised tool for assessing risk of bias in randomised trials. BMJ. 2019;366:l4898.
