# Supplementary material for: Quality of Evidence in Remote Monitoring of Patients With Liver Cirrhosis: A Systematic Review
Source: Gastro Hep Adv. 2026 May 8;5(8):100997. doi: 10.1016/j.gastha.2026.100997 (PMC13264051; doi:10.1016/j.gastha.2026.100997)
Supplement: Extended PDF [file mmc2.pdf]

Gastro Hep Advances 2026;5:100997

SYSTEMATIC REVIEWS AND META-ANALYSIS

Quality of Evidence in Remote Monitoring of Patients With Liver Cirrhosis: A Systematic Review

Britt van Ruijven,[1,2] Marten A. Lantinga,[3] Joost P. H. Drenth,[3] Marieke J. Pierik,[1,2] Tom J. G. Gevers,[1,2,*] and Govert Veldhuijzen[4,*]

aff_print: [1]Department of Gastroenterology and Hepatology, Maastricht University Medical Center, Maastricht, the Netherlands; [2]Division Liver & Digestive Health, Institute of Nutrition and Translational Research in Metabolism (NUTRIM), Maastricht University, Maastricht, the Netherlands; [3]Department of Gastroenterology and Hepatology, Amsterdam UMC, University of Amsterdam, Amsterdam Gastroenterology Endocrinology Metabolism, Amsterdam, the Netherlands; and [4]Department of Gastroenterology and Hepatology, Gelre Ziekenhuizen, Apeldoorn, the Netherlands

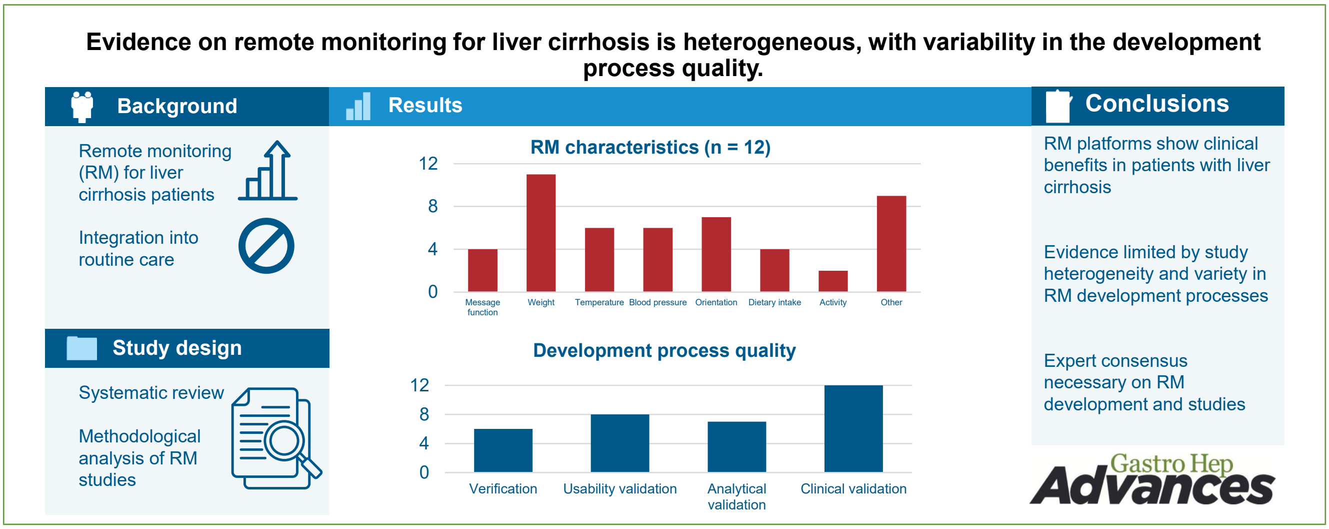

BACKGROUND AND AIMS: Remote monitoring (RM) enables out-of-hospital surveillance and may benefit patients with liver cirrhosis. However, implementation in routine liver cirrhosis care is limited, most likely due to the lack of conclusive data on clinical effectiveness. This systematic review aims to analyze the quality of evidence regarding the effect of RM tools on disease outcomes for liver cirrhosis. **METHODS:** This Preferred Reporting Items for Systematic Reviews and Meta-Analyses (PRISMA)-guided systematic review included studies of RM interventions per the World Health Organization's definition in patients with liver cirrhosis reporting at least 1 liver disease-related outcome. Retrospective or cross-sectional studies were excluded. Risk of bias was assessed using the Cochrane Risk Of Bias Tool for Randomized Trials and the Newcastle-Ottawa Scale for other study types. RM development quality was evaluated against the V3+ framework. **RESULTS:** One randomized controlled trial, 4 prospective cohorts with controls, and 7 prospective studies without controls were identified (n = 12; 636 patients; 17,680 studies screened). All studies with controls reported lower hospital readmissions in the RM groups. Interventions included smartphone applications, with or without additional measurement instruments, and automated calls. RM development quality varied substantially, with multiple RM interventions not reporting on verification (n = 6), usability (n = 4), or analytical validation (n = 5). The identified evidence informed the synthesis of a proposal framework to harmonize future RM research. **CONCLUSION:** Although studies show clinical benefits of RM tools in liver cirrhosis, evidence is limited by the (inherent) heterogeneity in interventions, design, and outcomes, and quality of the RM development process. This review emphasizes the need for consensus on RM monitoring frameworks and high-quality data to inform better clinical decision-making.

Keywords: Telehealth; Telemedicine; e-Health; End-Stage Liver Disease

*Denotes co-last authorship.

Abbreviations used in this paper: **ALD, alcohol-related liver disease; HCV, hepatitis C virus; IBD, inflammatory bowel disease; MASLD, metabolic dysfunction–associated steatotic liver disease; NOS, Newcastle-Ottawa Scale; RCT, randomized controlled trial; RM, remote monitoring; SLD, steatotic liver diseases.**

Most current article

© 2026 The Author(s). Published by Elsevier Inc. on behalf of American Gastroenterological Association Institute. This is an open access article under the CC BY license (http://creativecommons.org/licenses/by/4.0/).
2772-5723
https://doi.org/10.1016/j.gastha.2026.100997

Introduction

Healthcare delivery, including the management of liver cirrhosis, is increasingly incorporating telehealth, particularly through remote monitoring (RM).[1–5] RM facilitates the surveillance of a patient's condition outside the hospital, using technologies such as connected sensors and digital platforms.[6] In patients with liver cirrhosis, RM platforms have demonstrated promising findings regarding disease-related benefits and cost-effectiveness.[5]

RM can support patient empowerment and could help in the earlier recognition of subtle changes preceding decompensation events in patients with liver cirrhosis. Several prospective studies and reviews have emphasized the potential of RM tools for various liver disease-related events.[5,7] Despite encouraging early results, RM remains inconsistently integrated into routine liver cirrhosis care. Substantial heterogeneity in study designs, RM modalities, and reported outcomes constrains the synthesis of conclusive findings and has recurrently been acknowledged as a barrier for real-world translation and successful implementation.[8–13] Available systematic reviews on this topic focused on patients with decompensated liver cirrhosis while excluding studies without a control group or short follow-up, potentially missing out on promising technologies, or included populations with different needs, such as those infected with hepatitis C virus (HCV) without cirrhosis or post-transplant patients.[9–13] Furthermore, current evidence has not yet been evaluated against structured quality validation frameworks for the development of digital tools.

This systematic review aims to provide a comprehensive methodological analysis of the quality of evidence and existing knowledge gaps regarding the effect of RM tools on disease outcomes in compensated and decompensated liver cirrhosis. In addition, it assesses current evidence against a structured quality framework for validation of digital tools and proposes a framework informed by the identified evidence base to harmonize future research and improve the impact of RM interventions on liver cirrhosis patient care.

Methods

Search Strategy

For this systematic review, the Preferred Reporting Items for Systematic Reviews and Meta-Analyses (PRISMA) guideline for reporting systematic reviews was used (Supplementary Table 1).[14,15] The search strategy was developed in collaboration with a librarian with expertise in health sciences literature to optimize sensitivity. This review was registered in PROSPERO, an international systematic review registry produced by the Centre for Reviews and Dissemination (CRD), with the number CRD42024620147.

The following databases were searched, with the final search on January 14, 2026: Medline via Ovid, Embase via Ovid, Web of Science, Cochrane Library, the PROSPERO database of systematic reviews, ClinicalTrials.gov, CIHNAHL via EBSCO, and Epistemonikos Health Evidence. Clinicaltrialsregister.eu and the International Clinical Trials Registry Platform Search Portal were screened to identify studies in preparation. Snowballing was performed on titles in the bibliographies of eligible studies. We did not use any date restrictions. Search strategies for each database and deduplication strategies are outlined in Supplementary Tables 2 and 3.

Study Selection and Eligibility Criteria

B.R. and T.G. independently screened titles and abstracts via Covidence systematic review software; both reviewers were blinded to each other's decisions.[16] Full texts were reviewed if the reviewers could not determine a study's eligibility through title and abstract screening. Any disagreements were resolved through consensus meetings with a third reviewer (G.V.) when necessary.

Studies evaluating patients with liver cirrhosis were selected. Studies assessing patients living with hepatitis B virus and HCV, metabolic dysfunction–associated steatotic liver disease (MASLD), alcohol-related liver disease (ALD), and autoimmune hepatitis patients were included if separate liver cirrhosis data were available. We anticipated that many studies encompassed pilot phases; therefore, studies were also eligible for inclusion even if they did not include a comparative group. The intervention must consist of technologies used for RM, for example, electronic portals, wearables, digital questionnaires, or message functions. We used the World Health Organization's definition of RM: services that enable health-care providers to monitor an individual's condition remotely, using technologies such as implanted devices and sensors with wireless or wired connections.[6] Publications required evaluation of at least 1 clinical outcome, reported as either primary or secondary outcomes: liver cirrhosis–related complications (hepatic encephalopathy, [refractory] ascites, gastrointestinal bleeding, acute kidney injury-hepatorenal syndrome), hospital admissions, mortality, transjugular intrahepatic portosystemic shunt–free survival, transplant-free survival, or treatment adherence.

Study Designs

We included the following study designs: randomized controlled trials (RCTs), non-RCTs, prospective cohort studies, routine health-care databases, uncontrolled and controlled before-and-after studies (including interrupted time series), case-control studies, observational studies, epidemiological studies, experimental or quasi-experimental studies, and feasibility studies. Studies with alternative study designs were evaluated for eligibility in a consensus meeting. We assessed individual studies used in reviews and meta-analyses for eligibility.

We excluded retrospective and cross-sectional study designs, as they are less suitable for assessing the causal relationship between interventions and clinical outcomes, the focus of this review.[17] Additional exclusion criteria comprised no availability in the English language, assessment of first-line RM without involvement of gastroenterologists/hepatologist, exclusive assessment of telephone or video consultations between patient and health-care provider, between patients, or between health-care providers, and insufficient data.

Data Extraction and Study Quality Assessment Methods

Data were collected in standardized data extraction forms in Excel. For homogeneity purposes, proof-of-concept studies and (prospective) pilot studies without a control group were

recorded as feasibility studies. Follow-up periods were converted to days, with 1 week representing 7 days and 1 month representing 30 days. Nonalcoholic fatty liver disease or nonalcoholic fatty liver disease terms for underlying etiologies were collectively reported as metabolic dysfunction–associated steatohepatitis and MASLD etiologies. We separately defined liver cirrhosis etiologies ALD, MASLD, metabolic and alcohol-related liver disease, as lifestyle-related causes and grouped other etiologies.

Quality of RCTs was assessed with the Cochrane Risk Of Bias Tool for Randomized Trials (RoB 2, Supplementary Figure 1).[18] All other study types received quality assessment using the Newcastle-Ottawa Scale (NOS) Quality assessment scores of observational cohort studies, a 0–9 scale (Supplementary Material 1, and Supplementary Table 4).[19] We defined "poor," "fair," and "good" quality as total NOS scores of 0–3, 4–6, and 7 or higher, respectively.

Remote Monitoring Development Quality Assessment Methods

RM interventions in studies were evaluated against the recently updated and widely adopted Digital Medicine Society's V3+ framework.[20,21] The Digital Medicine Society's V3+ framework provides one of the most widely adopted structures for the development process of sensors, also applicable to many RM systems.[20,21] The framework distinguishes 4 phases in the development process of digital health technologies: (1) verification, evaluating the performance or output of data of the RM tool against prespecified criteria; (2) usability validation, reporting whether the technology can be used with ease, efficiency, and user-satisfaction to achieve specified goals; (3) analytical validation, assessing whether the platform accurately measures, detects, or predicts physiological or behavioral metrics; and (4) clinical validation, which determines whether use of the technology leads to meaningful clinical benefits in the target population and situation. We assessed if included RM studies answered 4 questions, each representing a phase of the V3+ framework (Supplementary Table 6).[20–22] If studies referred to previously conducted research in which one or more of the phases of the framework were reported, we reported that they complied with the respective phase of the framework. We reported the verification phase as performed in case a quality certification was available, or the RM tool was federal regulation compliant, also if this data was not open-sourced.

Proposal for a Framework of Development and Evaluation Trajectories of Remote Monitoring Studies in Liver Cirrhosis

The included studies were categorized and synthesized to provide an overview of prevailing methodologies and to identify gaps in the current evidence base. We especially examined the key study characteristics, including study designs, recruitment sites, outcomes, singlecenter vs multicenter designs, the availability of a validation cohort, and follow-up duration.

Based on these observations, we aimed to move toward a more structured interpretation of the existing evidence by deriving an author-based consensus framework. This framework intends to help structure the development and evaluation of RM interventions in liver cirrhosis.

Results

Literature Search

Our search strategy identified 17,680 individual studies. We included a total of 12 studies (n = 8 full papers, n = 4 abstracts, Figure 1). Snowballing through citation tracking did not yield any additional inclusions.

Study Designs

The study designs included 1 RCT, 4 prospective cohorts with control groups, and 7 prospective cohorts without controls (Table 1). The majority of patients with liver cirrhosis were hospitalized at the time of inclusion (9/12). The primary study setting was a single academic liver transplant center (10/12). All control groups consisted of internal cohorts, of which 2 were historic populations. Follow-up durations ranged from 28 to 90 days. The RCT was a follow-up study on an open-label feasibility study with the Patient Buddy App and scored a "low risk of bias" in the Cochrane Risk of Bias Assessment Tool of RCTs (Table 1; Supplementary Figure 1).[23] Cohort studies were rated with "good" (n = 2) and "fair" (n = 9) overall quality, respectively, through the NOS (Supplementary Table 4).

Population Characteristics

Sample size in the RM group ranged from 19 to 116 patients (Table 2). Baseline disease severity was mostly reflected in the mean and median Model for End-stage Liver Disease or Model for End-stage Liver Disease-sodium scores, with a range of 13–19.5. The mean or median age ranged from 51 to 67.2 years. A total of 636 patients participated in RM, of whom the majority were male (62.9%). ALD was the predominant etiology of liver cirrhosis (36%, 207/583 reported), 164 (28%) were known with metabolic dysfunction–associated steatohepatitis or MASLD, and 212 (36%) had other etiologies.

Intervention Characteristics of Remote Monitoring

Seven studies employed smartphone applications as RM intervention, of which 4 incorporated additional wireless sensors/wearables, including weighing scales and physical activity trackers. Tablet applications were utilized in 3 studies. One study relied on automated, scheduled calls, while another did not specify the RM tool used, although heart rate, blood pressure, and weight were monitored (Table 3). Continuous monitoring was implemented in 1 study, whereas 5 studies applied daily monitoring protocols. Two reported weekly monitoring, and 1 publication described RM conducted 3 times per week in conjunction with various paramedical consultations. Body weight was the most frequently assessed parameter, being reported in 11 RM interventions. A message

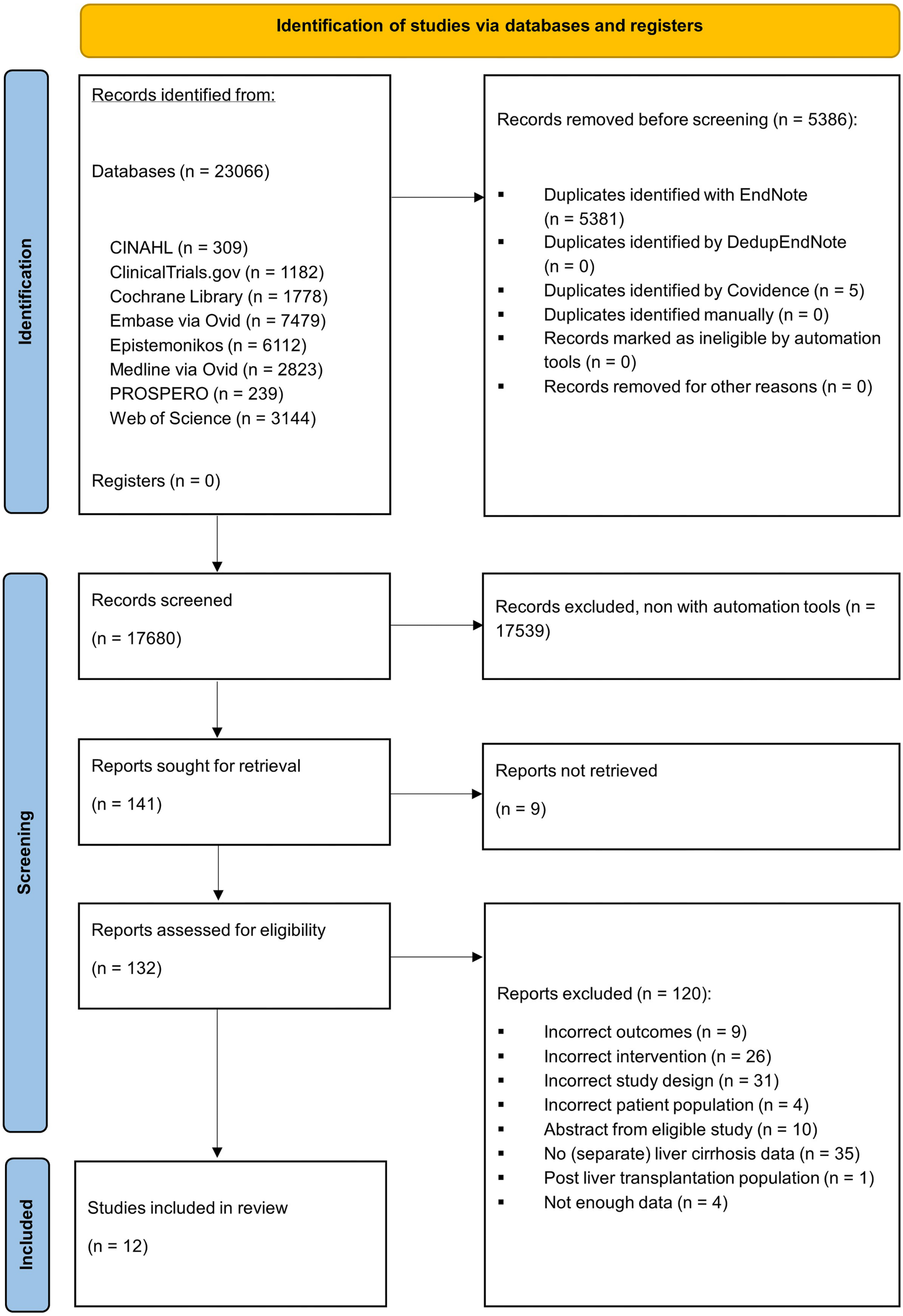

Figure 1. Preferred Reporting Items for Systematic Reviews and Meta-Analyses (PRISMA) 2020 flow diagram. Presenting the identification of 17,680 screened records and 12 included studies.

function was available in 4 RM tools. Most tools assessed multiple clinical or behavioral parameters, like orientation (7), temperature (6), blood pressure/heart rate (6), dietary intake (4), and physical activity (2).

Study Aims and Reported Outcomes

Two studies primarily evaluated the effect of RM on clinical outcomes.[23,26] Seven studies primarily aimed to pilot or define the feasibility of the RM intervention, for

Table 1. Study Designs

Design	Year	Author	Inpatient or outpatient recruitment	RM intervention	Primary outcome	Setting	Validation cohort	Follow-up duration
Randomized controlled trial	1. 2025	Shaw et al[23]	Inpatient	Smartphone application (Patient Buddy App and EncephalApp)	Avoidable readmissions	Three transplant centers, of which 2 academic centers	Internal	30 d
Prospective cohort with control group	2. 2023	Kazankov et al[7]	Inpatient	Smartphone application (CirrhoCare) with monitoring devices: (1) Wristwatch (Withings Move) (2) Blood pressure (BP) cuff (3) Weighing scales (Withings Body+) with bioimpedance	Feasibility of daily remote management	Single academic transplant center	Internal, matched controls	84 d
	3. 2024	Penrice et al[24]	Inpatient	Tablet with Bluetooth-enabled vital signs uploads from blood pressure monitor, pulse oximeter, and weighing scale	Qualitative aspects of design,	implementation, and patient satisfaction	Single academic transplant center	Internal, matched retrospective controls 90 d
	4. 2017	Khungar et al[25] Abstract	Inpatient	Tablet with wireless blood pressure monitor, pulse oximeter, and weighing scale	*Not reported*	Single academic transplant center	Internal	90 d
	5. 2024	Ballesteros et al[26] Abstract	Inpatient	Smartphone application (DOCCLA's digital platform)	*Not reported*	Single academic transplant center	Internal, retrospective controls	42 d intervention 90 d follow-up
Prospective cohort without control group	6. 2022	Lin et al[27]	Outpatient	Wrist personal activity trackers (PAT) and smartphone application (EL-FIT)	Incidental hospital admission and mortality	Single academic transplant center.	No	*Predefined follow-up not reported. Median follow-up 223 d.*
	7. 2015	Thomson et al[28]	Inpatient	Proactive automated scheduled calls	Time to first hospital admission	Single academic transplant center	No	90 d
	8. 2025	Ngu et al[29]	Inpatient and outpatient	Smartphone application (LivR well) with Bluetooth weighing scales, and a cloud-based database for referral, screening and data collection	Acceptability and feasibility	Single academic liver transplant center	No	28 d intervention 84 d follow-up

Table 1. Continued

Design	Year	Author	Inpatient or outpatient recruitment	RM intervention	Primary outcome	Setting	Validation cohort	Follow-up duration
	9. 2020	Bloom et al[30]	Inpatient and outpatient	Smartphone application with Bluetooth weighing scale connected to EMR	% enrolled d during which weight data were successfully transmitted to the EMR and the % weight alerts that prompted a response by the provider	Single academic transplant center	No	*Predefined follow-up: 28 d.* *Actual follow-up: 979 patient-d.*
	10. 2021	Qian et al[31] Abstract	Inpatient	*Specific devices not reported.*	*Not reported*	Single academic transplant center	No	90 d
	11. 2018	Verma et al[32] Abstract	Inpatient	Tablet application with web-based provider portal to retrieve the results	*Not reported* *Overall purpose: assess feasibility*	*Not reported*	No	90 d
	12. 2017	Ganapathy et al[33]	Inpatient	Smartphone application (Patient Buddy App)	Total 30-d admissions, HE-related readmissions, dropouts	Single academic transplant center	No	30 d

EL-FIT, Exercise and Liver FITness; EMR, electronical medical record.

Table 2. Population Characteristics

Author	Inpatient or outpatient recruitment	N (control group vs intervention group)	Disease status at baseline (control group vs intervention group)	% liver cirrhosis etiologies (control group vs intervention group)		% males (control group vs intervention group)	Age (y) (control group vs intervention group)
Shaw et al[23]	Inpatient	116 vs 116 dyads[c]	MELD-Na: 18.9 ± 8.4 vs 16.9 ± 7.9[a]	Alcohol<br>MASH/MASLD<br>Other	31.0 vs 31.0<br>26.7 vs 28.4<br>42.2 vs 40.5	65.5 vs 68.9	59.1 ± 12.38 vs 58.4 ± 8.88[a]
Kazankov et al[7]	Inpatient	20 vs 20	MELD-Na: 18 ± 4.6 vs 16.1 ± 4.2[a]	Alcohol<br>MASH/MASLD<br>Other	85 vs 80<br>10 vs 10<br>5 vs 10	45 vs 70	56 ± 14 vs 59 ± 10[a]
Penrice et al[24]	Inpatient	74 vs 41	MELD-Na: 17.3 (13.0–18.42) vs 15.1 (9.8–18.3)[b]	Alcohol<br>MASH/MASLD<br>Other	52.3 vs 46.3<br>30.1 vs 26.8<br>17.6 vs 26.8	54.0 vs 58.5	62.4 ± 12.5 vs 60.9 ± 9.0[a]
Khungar et al[25] Abstract	Inpatient	143 vs 19	Median MELD-Na: 19<br>% Child-Pugh B: 57.9<br>% Child-Pugh C: 31.6<br>*Not reported for control group*	Alcohol<br>MASH/MASLD<br>Hepatitis C	*Not reported*<br>*Not reported*<br>53	*Not reported for control group*<br>% males intervention group: 47.4	*Not reported for control group*<br>Median age intervention group: 58
Ballesteros et al[26] Abstract	Inpatient	39 vs 43	Decompensated patients with ascites.<br>*MELD-Na or Child-Pugh score not reported*	*Not reported.*		41 vs 65	*Not reported.*
Lin et al[27]	Outpatient	116: 71 PAT, 45 PAT + EL-FIT application	MELD: 14.3 ± 5.9[a]<br>MELD-Na: 15.2 ± 6.9[a]<br>Child-Pugh score: 8 (5–10)[b]	Alcohol<br>MASH/MASLD<br>Other	33<br>30<br>37	55	56 ± 11[a]<br>*Inclusion criteria: age 40–70 y*
Thomson et al[28]	Inpatient	79	Mean MELD: 13 (6–40)<br>Mean Child-Pugh score: 8 (6–13)	Alcohol<br>MASH/MASLD/ cryptogenic<br>Other	11<br>39<br>49	51	57 (23–85)[b]
Ngu et al[29]	Inpatient and outpatient	59	Patients with ACLF<br>MELD: 16 (12–21)[b]<br>% Child-Pugh A: 22<br>% Child-Pugh B/C: 78<br>*Inclusion criteria: MELD ≥ 10*	Alcohol<br>MASH/MASLD<br>Other	74<br>5<br>21	66	51 (37–59)[b]

Table 2. Continued

Author	Inpatient or outpatient recruitment	N (control group vs intervention group)	Disease status at baseline (control group vs intervention group)	% liver cirrhosis etiologies (control group vs intervention group)		% males (control group vs intervention group)	Age (y) (control group vs intervention group)
Bloom et al[30]	Inpatient and outpatient	25	Patients actively receiving ascites management.<br>MELD: 15.8 ± 5.9[a]	Alcohol<br>MASH/MASLD<br>Other	44<br>36<br>20	48	57.6 ± 12.8[a]
Qian et al[31] Abstract	Inpatient	48<br>17 dropped out immediately	Mean MELD-Na: 13.5 (6–24)<br>% Child-Pugh A: 33<br>% Child-Pugh B/C: 67	Alcohol<br>MASH/MASLD<br>Other	34<br>50<br>16	100	Mean age: 67.2 (47–78)
Verma et al[32] Abstract	Inpatient	30	Mean MELD-Na: 19.5 (11–29)<br>Inclusion criteria: ≥1 liver disease related complication.<br>Exclusion criteria: patients discharged to nursing homes.	Alcohol<br>MASH/MASLD<br>Other	40<br>10<br>50	60.0	Mean age: 55.7 (28–71)
Ganapathy et al[33]	Inpatient	40	MELD: 19.5 ± 5.2[a]	Alcohol<br>MASH/MASLD<br>Other	17.5<br>32.5<br>50	60	58 ± 10[a]

EL-FIT, Exercise and Liver FITness; MASH, metabolic dysfunction–associated steatohepatitis; MELD-Na, Model for End-stage Liver Disease-sodium; PAT, physical activity tracker.
[a]Mean ± SD.
[b]Median (IQR).
[c]Dyad: a cirrhosis inpatient and an adult caregiver.

Table 3. Intervention Characteristics of Remote Monitoring

Author	Aim	RM frequency	Message function	Weight	Temperature	Blood pressure and heart rate	Orientation	Dietary intake	Activity	Other:
Shaw et al[23]	Reducing 30-d readmissions.	Daily monitoring: Weight and temperature.<br>Weekly monitoring: Other parameters.	✓	✓	✓<br>Self-reported	x	✓<br>EncephalApp and judged by caregiver	✓<br>Sodium intake	x	Self-reported:<br>Bowel movements<br>GI-bleeding<br>Infections<br>Medication adherence
Kazankov et al[7]	Assess feasibility and patient engagement of monitoring advanced cirrhosis.	Daily monitoring	✓<br>Text and voice messages	✓	x	✓	✓	✓	✓<br>Wristwatch	x
Penrice et al[24]	1. Design and implement a cirrhosis-specific RPM (CiRPM) program.<br>2. Show utility through measurement of potential clinical benefit, patient satisfaction, and patient adherence.	Daily monitoring: Parameters.<br>Every 3 d: six alternating liver-complication yes-no questions.	✓	✓	✓	✓	x	x	x	Self-reported:<br>Bowel movements<br>Changes in attention<br>Swollen ankles<br>Abdominal tension<br>Shortness of breath<br>Missed medication
Khungar et al[25] Abstract	Pilot a wireless mobile device monitoring system to detect early symptoms and signs, thereby preventing readmissions and keeping patients engaged and feeling cared for daily.	*RM frequency not reported*	*Not reported*	✓	x	✓	✓	x	x	Self-reported:<br>Hepatic encephalopathy<br>Fluid overload<br>GI bleeding<br>Infections<br>Medication adherence<br>*All not specifically reported.*
Ballesteros et al[26] Abstract	Demonstrate the impact of real-world remote monitoring of patients with decompensated cirrhosis.	*RM frequency not reported*	*Not reported*	✓	✓	✓	*Not reported*	*Not reported*	*Not reported*	Liver-related symptoms/ complaints, *not specifically reported.*

Table 3. Continued

Author	Aim	RM frequency	Message function	Weight	Temperature	Blood pressure and heart rate	Orientation	Dietary intake	Activity	Other:
Lin et al[27]	Assess the utility of daily step count in predicting hospital admission and mortality rates.	Continuous monitoring and promotion of exercise training	x	x	x	✓<br>Heart rate	x	x	✓	x
Thomson et al[28]	Test the IVR system and determine if gathered information can predict complications.	Weekly IVR calls.	x	✓<br>Self-reported	x	x	✓<br>Self-reported	x	x	Self-reported:<br>Swelling<br>Jaundice<br>Weakness<br>Medication changes<br>Overall health
Ngu et al[29]	Investigate the acceptability and feasibility of a multimodal community intervention for ACLF.	Three times per week, home nursing.<br>Weekly medical review.<br>“Regular” dietitian, and pharmacy consultations, with adjunct interventions by physiotherapy, social work, addiction medicine, or neuropsychiatry.	*Not reported*	✓	x	x	x	x	x	Home nursing to assess liver-related symptoms<br>Medical review and urine (ETG) sample to assess alcohol use.
Bloom et al[30]	Evaluate the feasibility of a smartphone app in facilitating outpatient ascites management.	Every weekday: Weight monitoring	x	✓	x	x	x	x	x	x
Qian et al[31] Abstract	*Not reported*	*RM frequency not reported*	*Not reported*	✓	✓<br>Self-reported	✓	✓<br>Self-reported	✓<br>Self-reported salt intake	x	Self-reported:<br>Bowel movements<br>Swelling<br>Shortness of breath<br>Dizziness<br>Falls<br>Pain<br>Medication<br>Alcohol use

Table 3. Continued

Author	Aim	RM frequency	Message function	Weight	Temperature	Blood pressure and heart rate	Orientation	Dietary intake	Activity	Other:
Verma et al[32] Abstract	Assess the feasibility and utility of TeleMonitoring of Symptoms and Cognitive function/ TeMSCog for ESLD inpatients.	Daily questions about symptoms on a 0–10 scale, including pain, depression, and anxiety	*Not reported*	✓	✓	x	✓ EncephalApp	x	x	Liver-related symptoms/ complaints, *not specifically reported.* Self-reported: Breathlessness Pain Depression Anxiety Fatigue
Ganapathy et al[33]	Define the feasibility of a smartphone app and its impact on 30-d readmissions.	Daily monitoring: Weight and temperature. Weekly monitoring: Other parameters	✓	✓	✓ Self-reported	x	✓ EncephalApp and judged by caregiver	✓ Sodium intake	x	Self-reported: Bowel movements GI-bleeding Infections Medication adherence Judged by caregiver: Fall risk Time up and go test

ACLF, acute on chronic liver failure; CiRPM, Cirrhosis-specific Remote Monitoring Program; ESLD, end-stage liver disease; ETG, ethyl glucuronide; GI, gastrointestinal; IVR, interactive voice response; TeMSCog, TeleMonitoring of Symptoms and Cognitive function.

Table 4. Reported Outcomes

Author	Hospital (re)admissions	Patient contacts, medication changes, or interventions	Mortality	Liver transplantation or workup	RM adherence	RM evaluation by patients	Other reported outcomes
Shaw et al[23]	✓ Including avoidable readmissions[a]	✓	x	✓	✓	✓	LVPs
Kazankov et al[7]	✓	✓	✓	✓	✓	✓	Unplanned LVPs<br>Changes MELD-Na and CLIF-C AD scores
Penrice et al[24]	✓	x	✓	x	✓	✓	LVPs
Khungar et al[25] Abstract	✓ Including avoidable readmissions[a]	x	x	x	x	x	x
Ballesteros et al[26] Abstract	✓[a]	✓[a]	✓[a]	x	x	x	Planned LVPs<br>Decompensation rate
Lin et al[27]	✓	x	✓	x	x	x	Daily step count association with hospital admission and mortality, adjusted for MELD-Na and EL-FIT use
Thomson et al[28]	✓	x	✓	✓	✓	x	x
Ngu et al[29]	✓	x	✓	✓	✓	✓	Changes MELD-Na and Child-Pugh scores<br>Sarcopenia incidence<br>CLDQ and EuroQoL VAS scores (self-rated health questionnaires)<br>Costs
Bloom et al[30]	✓	✓	x	x	✓	✓	LVPs
Qian et al[31] Abstract	✓	x Mentioned, but no specific data reported	x	x	✓	x Mentioned, but no specific data reported	x

Table 4. Continued

Author	Hospital (re)admissions	Patient contacts, medication changes, or interventions	Mortality	Liver transplantation or workup	RM adherence	RM evaluation by patients	Other reported outcomes
Verma et al[32] Abstract	✓	✓	x	✓	x Mentioned, but no specific data reported	✓	x
Ganapathy et al[33]	✓	✓	x	✓	✓	✓	RM measurements: Changes in EncephalApp scores and up and go times

CLDQ, Chronic Liver Disease Questionnaire; CLIF-AD, Chronic Liver Failure Consortium Acute Decompensation; EL-FIT, Exercise and Liver FITness; LVP, large-volume paracentesis, MELD-Na, Model for End-stage Liver Disease-sodium.
[a]*P* value <.05 in favor of intervention group.

example, evaluate whether patients and health-care providers are willing and able to use the RM tool (Table 3). Two studies assessed both feasibility and RM effectiveness,[24,33] and 1 study did not report on the primary research aim.[31] Primary outcomes ranged from avoidable readmissions to incidental hospital or total admissions, and time to hospital admission (4/12), to feasibility and related subdomains (5/12). In 3 abstracts, primary outcomes were not specifically reported (Table 1). All studies addressed hospital (re)admissions after inclusion (Table 4). Half of the studies recorded patient contacts, medication changes, or other interventions and mortality, while adherence and satisfaction were outlined in 7 and 8 publications, respectively (Table 4). They all documented positive results regarding adherence and satisfaction, but the reporting variety limited a summary synthesis of these outcomes in this review.[7,23,24,28–33] Only Ngu et al presented specific cost outcomes of the intervention and assessed quality of life.[29]

Clinical Outcomes

All 5 studies with control groups demonstrated clinical benefits of RM. Although not powered for clinical outcomes, 3 reported statistically significant benefits regarding (preventable) hospital readmissions compared to the control group, ranging from 10 vs 19.8% after 30 days to 12 vs 28% (Table 4, Supplementary Table 5).[23,25,26] Ballesteros et al also presented a significant reduction in large-volume paracentesis and mortality.[26] Kazankov et al and Penrice et al reported similar positive trends in readmission rates, large-volume paracenteses, and mortality.[7,24] Thirty-day hospital readmission rates ranged from 15% to 42.5% in studies without a control group[29,33] and from 3.9% to 68% in studies with follow-up until 90 days (Supplementary Table 5).[27,28,30–32]

Remote Monitoring Development Quality Assessment

The Patient Buddy App, investigated by Shaw et al and Ganapathy et al, and the CirrhoCare platform of Kazankov et al presented reports on progress through all 4 phases of the V3+ quality framework (Figure 2, Supplementary Table 6, see Methods section for explanation of phases).[7,23,33] Half of the studies used an RM tool, of which verification, quantifying the accuracy of a measurement, was traceable.[7,23,26,27,30,33] Eight studies used an RM tool with reported usability, assessing platform ease of use and efficiency.[7,23,24,28–30,33] Analytical validation, monitoring whether the platform accurately captures the intended outcomes, was reported in RM applications of 7 studies.[7,23,24,27,30,32,33] Parts of clinical validation were performed in all studies, as they all reported on clinical outcomes of the RM platform in the target population.

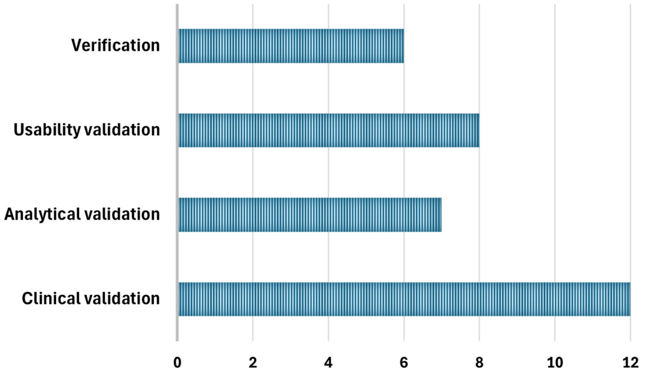

Figure 2. Number of studies reporting on remote monitoring development. Phases: verification (n = 6), usability validation (n = 8), analytical validation (n = 7), and clinical validation (n = 12).

Proposal for a Framework of Development and EEvaluation Trajectories of Remote Monitoring Studies in Liver Cirrhosis

The identified methodological strategies and gaps in the current evidence base synthesized a framework aimed to structure the development and evaluation of RM interventions in liver cirrhosis (Table 5). The framework comprises 4 sequential phases. First, early-phase feasibility studies should be conducted using harmonized methodologies to assess usability, adherence, and technical performance and to inform the selection of RM platforms for further evaluation. Second, prospective clinical evaluation should be undertaken in parallel with implementation efforts, enabling the generation of clinical evidence while facilitating integration into routine care. Third, confirmatory prospective trials should incorporate predefined core outcome sets, extended follow-up periods, multicenter designs, patient-reported outcome measures, patient-reported experience measures, and economic evaluations. Finally, external validation studies should assess generalizability across patient subgroups stratified by disease severity, comorbidity burden, and sociodemographic characteristics.

Discussion

Main Findings

This systematic review shows that RM in clinical care for patients with liver cirrhosis benefits the outpatient management of patients with liver cirrhosis. All studies with a control group, including 1 RCT, reported outcomes favoring RM, most commonly reflecting reductions in hospital readmissions or related healthcare utilization. The majority of included studies primarily assessed feasibility, consistently demonstrating high patient adherence and positive patient-reported experiences. Nonetheless, this review demonstrates the research gaps in RM interventions evaluated for disease outcomes in patients with liver cirrhosis. Most studies were small, single-center feasibility studies, with substantial variety in study design, intervention characteristics, and reported outcomes, limiting assessment of clinical endpoints. Evaluation using the V3+ framework revealed that reporting on verification, usability, and analytical validation was incomplete for several RM tools.

Signals of Clinical Potential and Feasibility

The included studies consistently demonstrated clinical effects in reducing hospital (re)admissions, as has been acknowledged in previous reviews, indicating a robust and coherent pattern across different study designs and settings.[5,11] High adherence and patient satisfaction emphasize the acceptability of digital tools in this population, including patients with lifestyle-related etiologies, supporting RM applicability in real-world contexts.[7,24] Importantly, the inclusion of patients with advanced cirrhosis is highly relevant, given the high readmission rates and potential health gains through RM.[34]

Heterogeneity as a Limiting Factor for Evidence Synthesis

In this study, we observed heterogeneity across different domains, characteristic of an emerging research field still in an exploratory and developmental phase. This includes variation in study objectives (feasibility vs effectiveness), intervention characteristics, outcome measures, and follow-up duration, complicating comparisons across studies. These observations are supported by statements by Gananandan et al, highlighting the need for standardization of digital tools across healthcare settings with generalizable components.[5] Evidence generation was further constrained by predominantly single-center designs, limited follow-up, and the frequent use of internal or retrospective control groups, limiting causal inference and increasing the risk of selection bias. Finally, the predominance of academic transplant populations and limited reporting on key feasibility determinants, like sociodemographic factors, raises concerns about the generalizability to broader hepatology settings, in compensated, outpatient groups, and low-resource contexts.[9] Certainly, these restrictions reflect the reality that developing, evaluating, and implementing innovations necessarily involves an iterative and practice-oriented approach.

Contextualization Within Existing Digital Health Literature

The clinical benefits of RM have been more extensively studied in patients with cardiac failure, as presented by meta-analyses that suggest a more advanced stage of RM development and methodological rigor. Despite this relatively robust evidence base, the evidence diversity has resulted in only a weak recommendation for non-invasive RM in the most recent European guidelines for acute and chronic cardiac failure.[35] Similarly, heterogeneity in study outcomes has been observed by Kumar et al in RM for patients with decompensated liver cirrhosis or

Table 5. Proposal for a Framework of Development and Evaluation Trajectories of RM Studies in Liver Cirrhosis

	Design	Recruitment site	Outcomes	Setting	Validation cohort	Follow-up duration
Most promising RM tools optimization	Feasibility (pilot) study	Inpatient	Primary:<br>• Dropout rate and time to dropout (usability and clinical validation)<br>• Compliance rate (usability validation)<br>• Patient and provider satisfaction through PROMs and PREMs (usability validation)<br>• Number of alerts (verification and analytical validation)<br>• Number of (alert-based) contacts and interventions (analytical and usability validation)<br>• Costs: healthcare consumption, RM use, QALY (clinical validation)<br>Secondary (clinical validation):<br>• Hospital readmission rate<br>• Mortality rate<br>• Decompensation events according to Baveno VII classification	Single or multicenter	Not applicable	≥6 mo
		Outpatient				
	Prospective cohort with control group, randomized or nonrandomized	Inpatient	Primary (clinical validation):<br>• Hospital readmission rate<br>• Mortality rate<br>• Decompensation events according to Baveno VII classification<br>Secondary:<br>• Dropout rate and time to dropout (usability and clinical validation)<br>• Compliance rate (usability validation)<br>• Patient and provider satisfaction through PROMs and PREMs (usability validation)<br>• Number of alerts (verification and analytical validation)<br>• Number of (alert-based) contacts and interventions (analytical and usability validation)<br>• Costs: healthcare consumption, RM use, QALY (clinical validation)	Multicenter: Secondary center, and academic transplant center	Prospective internal or external cohort matched for: age, sex, etiology, MELD-Na and Child-Pugh scores, treatment center, living situation, education, and digital health literacy	≥3 mo
		Outpatient				≥6 mo for decompensated patients at baseline<br>≥12 mo for compensated patients at baseline
	Prospective cohort comparing different RM characteristics, for example:<br>RM content<br>RM frequency<br>RM configurations, for example: additional vital parameters					

PREM, patient-reported experience measures; PROM, patient-reported outcome measures; QALY, quality-adjusted life year.

inflammatory bowel disease (IBD).[9] The extrapolability constraints identified in this review further align with findings in the IBD, chronic obstructive pulmonary disease (COPD), and acute care population, underscoring the pervasiveness of these limitations in the field of digital health across diseases.[8,36,37] This indicates that limited extrapolability likely reflects differences in RM development and scientific evaluation practices rather than disease-specific effects, necessitating a structured assessment of RM development and research.

Remote Monitoring Development Quality and Implications of the V3+ Framework Assessment

Accordingly, evaluating available RM projects along a systematic quality framework like the V3+ framework clarifies why cross-study comparability remains limited. By distinguishing between different stages of development, the assessment highlights that clinical validation alone is insufficient without reports on verification, usability testing, and analytical validation, aligning with the findings on intervention heterogeneity. As a result, most studies evaluating RM tools in cirrhosis primarily focus on technical feasibility rather than long-term outcomes or cost-utility, explaining the limited ability to identify the most clinically valuable RM configurations. Assessment through this framework underlines why current effect estimates remain preliminary.

Implications for Future Research

This review proposes a framework for the development and research trajectory of RM studies in liver cirrhosis, derived from the evidence gaps identified, to facilitate harmonization research and generation of comparable evidence. We recommend future research to build on the existing evidence base while explicitly distinguishing feasibility studies from evaluations of disease-related outcomes. Initial studies need to demonstrate reliable RM performance and consistent benefits, which are essential prerequisites for resource-intensive research and implementation strategies. Feasibility studies should prioritize the performance of monitoring of ascites and hepatic encephalopathy through body weight and orientation assessments, as these domains are currently most frequently evaluated (Table 3) and are likely to yield the largest clinical impact in follow-up studies, given their predominance as causes of hospital (re)admissions.[34,38] Following confirmation of feasibility and technical reliability, prospective evaluation studies should incorporate core outcomes, long-term follow-up, and multicenter designs. Notably, Muftah et al showed that telehealth in the HCV management achieves clinical outcomes comparable to standard care.[10] Accordingly, quality-of-care indicators like patient-reported outcome measures, patient-reported experience measures, and healthcare and societal costs should complement clinical endpoints to capture the total value of the intervention, as has been previously proposed in the IBD population.[8] Furthermore, increasing the inclusion of compensated patients may be advantageous for RM tools focusing on general well-being and prevention of a first decompensation event, given the adverse prognostic impact of decompensation.[39,40] To avoid dilution of observable clinical effects, future studies should consider stratifications or separate evaluations of compensated and decompensated patient populations. Ongoing large RCTs may further clarify the clinical benefits of RM, many of which use RM tools that have been presented in this review.[41–44] More importantly, collaboration and consensus among RM developers and hepatology experts are required to address the identified research gaps and optimize the design and impact of future research.

Strengths and Limitations

This review has several limitations. Studies focusing on self-management, without evaluating disease outcomes, were excluded, which may have led to the omission of promising pilot RM interventions. However, restricting inclusion to RM tools that have been evaluated for clinical outcomes ensured the analysis reflected their impact on clinical care. Study heterogeneity precluded quantitative analysis, including evaluation for publication bias and comparison of RM tools. However, this constitutes a bias inherent to the emerging field, as all starting RM platforms inevitably differ in certain technical or content aspects. Additionally, we highlight that the framework of development and evaluation trajectories of RM studies in liver cirrhosis is a proposal that has not been established within a consensus meeting but could serve as a start for consensus and collaboration.

Strengths include a comprehensive search strategy and a systematic overview of RM interventions across multiple modalities. Importantly, this review provides a pragmatic framework of methodological quality, highlights evidence gaps, and proposes directions to advance the research in the field.

Conclusion

RM for patients with liver cirrhosis promotes outpatient clinical care and improves hospital readmissions. However, evidence on RM for patients with liver cirrhosis remains fragmented and methodologically limited, with highly variable quality of development processes, precluding firm conclusions on clinical outcomes or comparisons of RM tools. Although feasibility studies show promise, they should naturally be distinguished from and followed by well-powered trials using standardized protocols, including secondary hepatology centers, core outcome sets, established follow-up durations, and matched (external) validation, as proposed in this review. We call for consensus and collaboration among RM and hepatology experts, which are essential to optimize RM development processes and future studies.

Meeting these requirements will provide a substantial boost to the research field and enable the successful implementation of RM in patients with liver cirrhosis.

Declaration of AI and AI-Assisted Technologies in the Writing Process

During the preparation of this work, the authors used ChatGPT in order to assist with language refinement and optimize readability in accordance with American English standards. The authors reviewed and edited the content as needed and take full responsibility for the content of the publication.

Supplementary Materials

Material associated with this article can be found, in the online version, at https://doi.org/10.1016/j.gastha.2026.100997.

References

1. Asch DA, Troxel AB, Goldberg LR, et al. Remote monitoring and behavioral economics in managing heart failure in patients discharged from the hospital: a randomized clinical trial. JAMA Intern Med 2022;182:643–649.
2. de Jong MJ, van der Meulen-de Jong AE, Romberg-Camps MJ, et al. Telemedicine for management of inflammatory bowel disease (myIBDcoach): a pragmatic, multicentre, randomised controlled trial. Lancet 2017; 390(10098):959–968.
3. Lee JA, Choi M, Lee SA, et al. Effective behavioral intervention strategies using mobile health applications for chronic disease management: a systematic review. BMC Med Inform Decis Mak 2018;18(1):12.
4. Noah B, Keller MS, Mosadeghi S, et al. Impact of remote patient monitoring on clinical outcomes: an updated meta-analysis of randomized controlled trials. NPJ Digit Med 2018;1:20172.
5. Gananandan K, Kazankov K, Tapper EB, et al. The new digital era in decompensated cirrhosis. Lancet Digit Health 2025;7:e54–e63.
6. Organization WH, Union IT. National eHealth strategy toolkit. Geneva: International Telecommunication Union World Health Organization, 2012.
7. Kazankov K, Novelli S, Chatterjee DA, et al. Evaluation of CirrhoCare R - a digital health solution for home management of individuals with cirrhosis. J Hepatol 2023; 78(1):123–132.
8. Gasparetto M, Narula P, Wong C, et al. Efficacy of digital health technologies in the management of inflammatory bowel disease: an umbrella review. Lancet Digit Health 2025;7(5):100843.
9. Kumar A, Gananandan K, Robinson G, et al. Limitations in real-world telemonitoring applicability in gastroenterology and hepatology: a systematic review. Frontline Gastroenterol 2026;17:19–29.
10. Muftah AA, Banala C, Raasikh T, et al. Telehealth interventions in patients with chronic liver diseases: a systematic review. Hepatology 2023;78(1):179–194.
11. Capuano P, Hileman B, Tigano S, et al. Telemedicine in patients affected by chronic liver disease: a scoping review of clinical outcomes and the devices evaluated. J Clin Med 2023;12(15):5128.
12. Hasan M, Bidargaddi N, Muller K, et al. Integrating smart phone applications in the management of cirrhotic patients: a scoping review. JGH Open 2023;7(12):826–831.
13. Lee S, Joo Y, Jang Y. eHealth self-management interventions for patients with liver cirrhosis: scoping review. J Med Internet Res 2025;27:e68650.
14. Page MJ, McKenzie JE, Bossuyt PM, et al. The PRISMA 2020 statement: an updated guideline for reporting systematic reviews. BMJ 2021;372:n71.
15. Rethlefsen ML, Kirtley S, Waffenschmidt S, et al. PRISMA-S: an extension to the PRISMA statement for reporting literature searches in systematic reviews. Syst Rev 2021;10(1):39.
16. Covidence systematic review software, Veritas Health Innovation. Melbourne, Australia; 2025.
17. Guyatt G, Oxman AD, Akl EA, et al. GRADE guidelines: 1. Introduction-GRADE evidence profiles and summary of findings tables. J Clin Epidemiol 2011;64(4):383–394.
18. Sterne JAC, Savovic J, Page MJ, et al. RoB 2: a revised tool for assessing risk of bias in randomised trials. BMJ 2019;366:l4898.
19. Wells G, Shea B, O'Connell D, et al. The Newcastle-Ottawa Scale (NOS) for assessing the quality of non-randomised studies in meta-analyses. https://www.ohri.ca/programs/clinical_epidemiology/oxford.asp. Accessed November 3, 2025.
20. Goldsack JC, Coravos A, Bakker JP, et al. Verification, analytical validation, and clinical validation (V3): the foundation of determining fit-for-purpose for Biometric Monitoring Technologies (BioMeTs). NPJ Digit Med 2020;3(1):55.
21. Bakker JP, Barge R, Centra J, et al. V3+ extends the V3 framework to ensure user-centricity and scalability of sensor-based digital health technologies. NPJ Digital Med 2025;8(1):51.
22. Applied Digital Health Ethics. Building fit-for-purpose sensor-based digital health technologies. Digital Medicine Society (DiMe) [Internet]. 2025. https://dimesociety.org/courses/building-fit-for-purpose-sensor-based-digital-health-technologies/. Accessed December 16, 2025.
23. Shaw J, Acharya C, Fagan A, et al. Health information technology interventions reduce avoidable readmissions in cirrhosis: the HEROIC randomized controlled trial. Hepatology 2026;83:1204–1214.
24. Penrice DD, Hara KS, Sordi-Chara B, et al. Design, implementation, and impact of a cirrhosis-specific remote patient monitoring program. Hepatol Commun 2024;8(8):e0498.
25. Khungar V, Serper M, Peyton D, et al. Use of an innovative telehealth platform to reduce readmissions and enable patient-centered care in cirrhotic patients. Hepatology 2017;66:94A–95A.
26. Ballesteros K, Kumaravel Kanagavelu AS, Crone J, et al. P126 improving patient outcomes in decompensated liver disease through remote monitoring: a real-world experience. Gut 2024;73(Suppl 3):A90.
27. Lin F-P, Bloomer PM, Grubbs RK, et al. Low daily step count is associated with a high risk of Hospital admission

and death in community-dwelling patients with cirrhosis. Clin Gastroenterol Hepatol 2022;20(8):1813–1820.e2.
28. Thomson M, Volk M, Kim HM, et al. An automated telephone monitoring system to identify patients with cirrhosis at risk of re-hospitalization. Dig Dis Sci 2015; 60(12):3563–3569.
29. Ngu NLY, Saxby E, Worland T, et al. A nonrandomized pilot study to investigate the acceptability and feasibility of LivR well: a multifaceted 28-day home-based liver optimization program for acute-on-chronic liver failure. Gastro Hep Adv 2025;4(2):100567.
30. Bloom P, Wang T, Marx M, et al. A smartphone app to manage cirrhotic ascites among outpatients: feasibility study. JMIR Med Inform 2020;8(9):e17770.
31. Qian LA, Higgins A, Zapatka S, et al. Development and feasibility of a disease management program for patients with cirrhosis at the Veterans Health Administration (VHA). Hepatology 2021;74(Suppl 1):375A.
32. Verma M, Kalman R, Walter JW, et al. Feasibility of telemonitoring of symptoms and cognitive function in end stage liver disease. Gastroenterology 2018; 154(6):S1221–S1222.
33. Ganapathy D, Acharya C, Lachar J, et al. The patient buddy app can potentially prevent hepatic encephalopathy-related readmissions. Liver Int 2017;37(12):1843–1851.
34. Bajaj JS, Reddy KR, Tandon P, et al. The 3-month readmission rate remains unacceptably high in a large North American cohort of patients with cirrhosis. Hepatology 2016;64(1):200–208.
35. Pizarro CS, Schots BBS, Schuuring MJ, et al. Non-invasive remote monitoring in heart failure: towards wearable devices and artificial intelligence solutions: short title: remote monitoring and wearable devices in heart failure. Curr Heart Fail Rep 2025;22(1):44.
36. Janjua S, Carter D, Threapleton CJ, et al. Telehealth interventions: remote monitoring and consultations for people with chronic obstructive pulmonary disease (COPD). Cochrane Database Syst Rev 2021;7(7):CD013196.
37. Sultani K, Smeulers M, de Vries R, et al. Transforming acute care: a scoping review on the effectiveness, safety and implementation challenges of hospital-at-home models. BMJ Open 2025;15(8):e098411.
38. Tapper EB, Halbert B, Mellinger J. Rates of and reasons for hospital readmissions in patients with cirrhosis: a multistate population-based cohort study. Clin Gastroenterol Hepatol 2016;14(8):1181–1188.e2.
39. D'Amico G, Garcia-Tsao G, Pagliaro L. Natural history and prognostic indicators of survival in cirrhosis: a systematic review of 118 studies. J Hepatol 2006; 44(1):217–231.
40. de Franchis R, Bosch J, Garcia-Tsao G, et al. Baveno VII - renewing consensus in portal hypertension. J Hepatol 2022;76(4):959–974.
41. Ngu NL, Saxby E, Worland T, et al. A home-based, multidisciplinary liver optimisation programme for the first 28 days after an admission for acute-on-chronic liver failure (LivR well): a study protocol for a randomised controlled trial. Trials 2022;23(1):744.
42. Greenham O, Gananandan K, Balaji A, et al. Prospective multicentre randomised controlled trial to assess the clinical effectiveness of the novel CirrhoCare digital therapeutic management system: a study protocol. BMJ Open 2025;15(7):e098725.
43. Use of a telehealth intervention to decrease readmissions in cirrhosis: a randomized controlled trial [Internet]. 2019. https://clinicaltrials.gov/study/NCT03969186. Accessed January 14, 2026.
44. Testing LiverWatch, a home-based remote-monitoring intervention for advanced liver disease [Internet]. 2023. https://clinicaltrials.gov/study/NCT06136221. Accessed January 14, 2026.

Received April 20, 2026. Accepted May 1, 2026.

Correspondence:
Address correspondence to: Britt van Ruijven, MD, Department of Gastroenterology and Hepatology, Maastricht University Medical Center, Postbus 5800, Maastricht 6202 AZ, the Netherlands. e-mail: britt.vanruijven@maastrichtuniversity.nl.

Acknowledgments:
The authors thank Remy van Alebeek from the Maastricht University Library Systematic Literature Review Support Team for his support in optimizing the search strategy.
Tom J.G. Gevers and Govert Veldhuijzen contributed equally to this study and are co-senior authors.

Authors' Contributions:
Govert Veldhuijzen, Tom J.G. Gevers, and Britt van Ruijven conceptualized and designed the study and interpreted the data. Britt van Ruijven developed the search strategy, performed the search, and drafted the manuscript. Tom J.G. Gevers and Britt van Ruijven screened the search results and performed the risk of bias assessment. Govert Veldhuijzen assisted in the consensus meetings to resolve disagreements. Govert Veldhuijzen, Joost P.H. Drenth, Marten A. Lantinga, Marieke J. Pierik, and Tom J.G. Gevers participated in the critical revision of the manuscript for important intellectual content.

Conflicts of Interest:
The authors disclose no conflicts.

Funding:
G. Veldhuijzen and Tom J.G. Gevers have received funding from the Dutch Digestive Health Fund, Gelre Wetenschapsfonds, and Gilead Sciences Inc. Marten A. Lantinga has received speaker fees from GORE (2024) and Takeda (2025) and a nonrestricted research grant from Vaillant fonds (2023). Joost P. H. Drenth is a consultant for CAMURUS, all proceeds go to the Amsterdam UMC. Marieke J. Pierik has received nonrestricted research grants from Horizon 2020, ZONMW (Dutch national research fund), the Dutch Digestive Health Fund, Takeda, Johnson & Johnson, AbbVie, and Galapagos, as well as nonfinancial support from Immunodiagnostics. Additionally, Marieke J. Pierik has received speaker's fees from BMS, Janssen Cilag, and Takeda, all outside the submitted work. The funders had no role in study design, data collection and analysis, decision to publish, or preparation of the manuscript.

Ethical Statement:
Ethical approval was not required as this was a systematic review, together with included studies, performed in accordance with the Declaration of Helsinki, for which no patients were recruited.

Data Transparency Statement:
The data used and/or analyzed during the current study are available from the corresponding author on reasonable request.

Reporting Guidelines:
PRISMA guideline for reporting systematic reviews.

Gastro Hep Advances, Volume 5

Supplemental information

Quality of Evidence in Remote Monitoring of Patients With Liver Cirrhosis: A Systematic Review

Britt van Ruijven, Marten A. Lantinga, Joost P.H. Drenth, Marieke J. Pierik, Tom J. G. Gevers, and Govert Veldhuijzen

Supplementary Materials

Title: Quality of evidence and research gaps in remote monitoring of liver cirrhosis patients: a systematic review.

Britt van Ruijven[1,2], Marten A. Lantinga[3], Joost P.H. Drenth[3], Marieke J. Pierik[1,2], Tom J.G. Gevers[1, 2, *], G. Veldhuijzen[4, *]

Table of contents

Pages 2-4	Table S1. PRISMA 2020 Checklist
Pages 5-17:	Table S2. Search strategies
Pages 18:	Table S3. Deduplication strategies
Page 19:	Table S4. Newcastle-Ottawa Scale Quality assessment scores of observational cohort studies
Page 20-21	Table S5. Clinical outcomes
Page 22:	Table S6. RM development quality assessment, according to V3+ framework
Page 23-24:	Material S1. Newcastle-Ottawa Scale Quality assessment scores of observational cohort studies
Page 25:	Figure S1. Cochrane Risk of Bias Assessment Tool of RCT’s (Rob2)
Page 26:	References

Table S1. PRISMA 2020 Checklist

Section and Topic	Item #	Checklist item	Location where item is reported
**TITLE**			
Title	1	Identify the report as a systematic review.	Title
**ABSTRACT**			
Abstract	2	See the PRISMA 2020 for Abstracts checklist.	Abstract
**INTRODUCTION**			
Rationale	3	Describe the rationale for the review in the context of existing knowledge.	Introduction
Objectives	4	Provide an explicit statement of the objective(s) or question(s) the review addresses.	Introduction and Materials and methods
**METHODS**			
Eligibility criteria	5	Specify the inclusion and exclusion criteria for the review and how studies were grouped for the syntheses.	Materials and methods
Information sources	6	Specify all databases, registers, websites, organisations, reference lists and other sources searched or consulted to identify studies. Specify the date when each source was last searched or consulted.	Materials and methods
Search strategy	7	Present the full search strategies for all databases, registers and websites, including any filters and limits used.	Materials and methods
Selection process	8	Specify the methods used to decide whether a study met the inclusion criteria of the review, including how many reviewers screened each record and each report retrieved, whether they worked independently, and if applicable, details of automation tools used in the process.	Materials and methods
Data collection process	9	Specify the methods used to collect data from reports, including how many reviewers collected data from each report, whether they worked independently, any processes for obtaining or confirming data from study investigators, and if applicable, details of automation tools used in the process.	Materials and methods
Data items	10a	List and define all outcomes for which data were sought. Specify whether all results that were compatible with each outcome domain in each study were sought (e.g. for all measures, time points, analyses), and if not, the methods used to decide which results to collect.	Materials and methods
	10b	List and define all other variables for which data were sought (e.g. participant and intervention characteristics, funding sources). Describe any assumptions made about any missing or unclear information.	Materials and

Section and Topic	Item #	Checklist item	Location where item is reported
			methods
Study risk of bias assessment	11	Specify the methods used to assess risk of bias in the included studies, including details of the tool(s) used, how many reviewers assessed each study and whether they worked independently, and if applicable, details of automation tools used in the process.	Materials and methods
Effect measures	12	Specify for each outcome the effect measure(s) (e.g. risk ratio, mean difference) used in the synthesis or presentation of results.	n.a.
Synthesis methods	13a	Describe the processes used to decide which studies were eligible for each synthesis (e.g. tabulating the study intervention characteristics and comparing against the planned groups for each synthesis (item #5)).	n.a.
	13b	Describe any methods required to prepare the data for presentation or synthesis, such as handling of missing summary statistics, or data conversions.	n.a.
	13c	Describe any methods used to tabulate or visually display results of individual studies and syntheses.	Materials and methods
	13d	Describe any methods used to synthesize results and provide a rationale for the choice(s). If meta-analysis was performed, describe the model(s), method(s) to identify the presence and extent of statistical heterogeneity, and software package(s) used.	n.a.
	13e	Describe any methods used to explore possible causes of heterogeneity among study results (e.g. subgroup analysis, meta-regression).	Results
	13f	Describe any sensitivity analyses conducted to assess robustness of the synthesized results.	n.a.
Reporting bias assessment	14	Describe any methods used to assess risk of bias due to missing results in a synthesis (arising from reporting biases).	n.a.
Certainty assessment	15	Describe any methods used to assess certainty (or confidence) in the body of evidence for an outcome.	n.a.
**RESULTS**			
Study selection	16a	Describe the results of the search and selection process, from the number of records identified in the search to the number of studies included in the review, ideally using a flow diagram.	Results
	16b	Cite studies that might appear to meet the inclusion criteria, but which were excluded, and explain why they were excluded.	n.a.
Study characteristics	17	Cite each included study and present its characteristics.	Results
Risk of bias in studies	18	Present assessments of risk of bias for each included study.	Results
Results of individual studies	19	For all outcomes, present, for each study: (a) summary statistics for each group (where appropriate) and (b) an effect estimate and its precision (e.g. confidence/credible interval), ideally using structured tables or plots.	n.a.
Results of syntheses	20a	For each synthesis, briefly summarise the characteristics and risk of bias among contributing studies.	Results
	20b	Present results of all statistical syntheses conducted. If meta-analysis was done, present for each the summary estimate and its precision (e.g.	Results

Section and Topic	Item #	Checklist item	Location where item is reported
		confidence/credible interval) and measures of statistical heterogeneity. If comparing groups, describe the direction of the effect.	
	20c	Present results of all investigations of possible causes of heterogeneity among study results.	Results
	20d	Present results of all sensitivity analyses conducted to assess the robustness of the synthesized results.	Results
Reporting biases	21	Present assessments of risk of bias due to missing results (arising from reporting biases) for each synthesis assessed.	Results
Certainty of evidence	22	Present assessments of certainty (or confidence) in the body of evidence for each outcome assessed.	n.a.
**DISCUSSION**			
Discussion	23a	Provide a general interpretation of the results in the context of other evidence.	Discussion
	23b	Discuss any limitations of the evidence included in the review.	Discussion
	23c	Discuss any limitations of the review processes used.	Discussion
	23d	Discuss implications of the results for practice, policy, and future research.	Discussion
**OTHER INFORMATION**			
Registration and protocol	24a	Provide registration information for the review, including register name and registration number, or state that the review was not registered.	Methods and materials
	24b	Indicate where the review protocol can be accessed, or state that a protocol was not prepared.	Methods and materials
	24c	Describe and explain any amendments to information provided at registration or in the protocol.	n.a.
Support	25	Describe sources of financial or non-financial support for the review, and the role of the funders or sponsors in the review.	Title page
Competing interests	26	Declare any competing interests of review authors.	Title page
Availability of data, code and other materials	27	Report which of the following are publicly available and where they can be found: template data collection forms; data extracted from included studies; data used for all analyses; analytic code; any other materials used in the review.	Title page

From: Page MJ, McKenzie JE, Bossuyt PM, Boutron I, Hoffmann TC, Mulrow CD, et al. The PRISMA 2020 statement: an updated guideline for reporting systematic reviews. BMJ 2021;372:n71. doi: 10.1136/bmj.n71. This work is licensed under CC BY 4.0. To view a copy of this license, visit https://creativecommons.org/licenses/by/4.0/

Table S2. Search strategies

Concept	Search string 29-11-2024 MEDLINE via Ovid	Results
Remote Monitoring	Exp "Computers, Handheld"/ or exp "Digital Health"/ or exp "Mobile applications"/ or exp "Monitoring, ambulatory"/ or exp "Point-of-Care Systems"/ or exp "Rapid Diagnostic Tests"/ or exp "Rapid On-site Evaluation"/ or exp "Telemedicine"/ or "Telemetry"/ or "Videoconferencing"/ or (App or Apps or Desktop or (Digital ADJ3 (care* or consult* or diagnos* or health* or medicine or monitor* or rehab* or therap* or visit*)) or Ehealth* or E-health* or “E-counseling” or “E-Therap*” or (Electronic* ADJ3 (app* or health* or medicine*)) or Emedicine or “E-medicine” or EncephalApp or "Distan* Counsel*" or ((Handheld OR "Hand Held") ADJ3 (computer* or device*)) or “Health app*” or (Home ADJ3 (medicine or monitor*)) or iPad or Laptop or (Medical ADJ3 (app*)) or Mhealth or “M-health*” or (Mobile ADJ3 (app* or computer or device* or health* or medicine* or monitor*)) or "Outpatient Monitor*" or "Patient* Monitor*" or ("Point-of-care" ADJ3 (test* or diagnos*)) or "Rapid Diagnos*" or "Rapid on-site diagnos*" or "Rapid on-site evaluation" or (Remote ADJ3 (care* or consul* or diagnos* or health* or manag* or medicine* or monitor* or rehab*)) or Smartphone* or “smart-phone*” or Smartwatch or Tablet or Telecare* or Teleconsul* or Telediagnos* or Telehealth* or Telemedicine* or Telemonitor* or Telemetr* or “Tele-metr*” or Telerehab* or Telerefer* or Teletherap* or Televisit* or (Tele ADJ3 (care* or consult* or diagnos* or health* or manag* or medicine* or monitor* or rehab* or refer* or therap* or visit*)) or Thealth* or “T-health*” or Videoconsult* or “Video Consult*” or Videoconf* or “Video-conf*” or (Virtual ADJ3 (care* or clinic* or consult* or health* or medicine* or monitor* or rehab* or therap* or visit*)) or Watch).ti,ab,kf.	460495
Cirrhosis	Exp "Hepatic Insufficiency"/ or exp "Hepatopulmonary syndrome"/ or exp "Hepatorenal syndrome"/ or exp "Hypertension, portal"/ or exp "Liver cirrhosis"/ or exp Ascites/ or (Ascites or Cirrh* or ((Chronic*) ADJ3 (liver OR hepatic*)) or CLD or Encephalopath* or (("end stage" or "late stage") ADJ3 (liver* or hepatic*)) or ESLD or (esophag* ADJ3 (varix* or varic*)) or (oesophag* ADJ3 (varix* or varic*)) or (gastric* ADJ3 (varix* or varic*)) or ((liver or hepatic*) ADJ3 (fibrosis or fail* or insufficien* or dysfunction*)) or "hepatopulmonary syndrome*" or "hepato-pulmonary syndrome*" or "hepatorenal syndrome*" or "hepato-renal syndrome*" or "portal hypertension" or (varic* ADJ3 (hemorrhag* or bleed*)) or (varix* ADJ3 (hemorrhag* or bleed*))).ti,ab,kf.	374560
(Remote) Monitoring AND Cirrhosis	1. AND 2.	2440
**Concept**	**Search string 14-07-2025 MEDLINE via Ovid**	**Results**
Remote Monitoring	Identical	490254

Cirrhosis	Identical	386509
(Remote) Monitoring AND Cirrhosis	1. AND 2.	2630
**Concept**	**Search string 12-01-2026 MEDLINE via Ovid**	**Results**
Remote Monitoring	Identical	516107
Cirrhosis	Identical	396961
(Remote) Monitoring AND Cirrhosis	1. AND 2.	2823
**Concept**	**Search string 29-11-2024 Embase via Ovid**	**Results**
Remote Monitoring	exp personal digital assistant/ or exp "Digital Health"/ or exp "Mobile application"/ or exp "ambulatory monitoring"/ or exp "Point-of-Care System"/ or exp "Rapid Test"/ or exp "Rapid On-site Evaluation"/ or exp "Telemedicine"/ or "Telemetry"/ or "Videoconferencing"/ or (App or Apps or Desktop or (Digital ADJ3 (care* or consult* or diagnos* or health* or medicine or monitor* or rehab* or therap* or visit*)) or Ehealth* or E-health* or E-counseling or E-Therap* or (Electronic* ADJ3 (app* or health* or medicine*)) or Emedicine or “E-medicine” or EncephalApp or "Distan* Counsel*" or ((Handheld OR "Hand Held") ADJ3 (computer* or device*)) or “Health app*” or (Home ADJ3 (medicine or monitor*)) or iPad or Laptop or (Medical ADJ3 (app*)) or Mhealth or “M-health*” or (Mobile ADJ3 (app* or computer or device* or health* or medicine* or monitor*)) or "Outpatient Monitor*" or "Patient* Monitor*" or ("Point-of-care" ADJ3 (test* or diagnos*)) or "Rapid Diagnos*" or "Rapid on-site diagnos*" or "Rapid on-site evaluation" or (Remote ADJ3 (care* or consul* or diagnos* or health* or manag* or medicine* or monitor* or rehab*)) or Smartphone* or “smart-phone*” or Smartwatch or Tablet or Telecare* or Teleconsul* or Telediagnos* or Telehealth* or Telemedicine* or Telemonitor* or Telemetr* or “Tele-metr*” or Telerehab* or Telerefer* or Teletherap* or Televisit* or (Tele ADJ3 (care* or consult* or diagnos* or health* or manag* or medicine* or monitor* or rehab* or refer* or therap* or visit*)) or Thealth* or “T-health*” or Videoconsult* or “Video Consult*” or Videoconf* or “Video-conf*” or (Virtual ADJ3 (care* or clinic* or consult* or health* or medicine* or monitor* or rehab* or therap* or visit*)) or Watch).ti,ab,kf.	615390
Cirrhosis	exp "Liver failure"/ or exp "Hepatopulmonary syndrome"/ or exp "Hepatorenal syndrome"/ or exp "Portal hypertension"/ or exp "Liver cirrhosis"/ or exp Ascites/ or (Ascites or Cirrh* or (Chronic* adj3 (liver or hepatic*)) or CLD or Encephalopath* or (("end stage" or "late stage") adj3 (liver* or hepatic*)) or ESLD or (esophag* adj3 (varix* or varic*)) or (oesophag* adj3 (varix* or varic*)) or (gastric* adj3 (varix* or varic*)) or ((liver or hepatic*) adj3 (fibrosis or fail* or insufficien* or dysfunction*)) or "hepatopulmonary	588901

	syndrome*" or "hepato-pulmonary syndrome*" or "hepatorenal syndrome*" or "hepato-renal syndrome*" or "portal hypertension" or (varic* adj3 (hemorrhag* or bleed*)) or (varix* adj3 (hemorrhag* or bleed*))).ti,ab,kf.	
(Remote) Monitoring AND Cirrhosis	1. AND 2.	5944
**Concept**	**Search string 14-07-2025 Embase via Ovid**	**Results**
Remote Monitoring	Identical	689398
Cirrhosis	Identical	618577
(Remote) Monitoring AND Cirrhosis	1. AND 2.	6901
**Concept**	**Search string 13-01-2026 Embase via Ovid**	**Results**
Remote Monitoring	Identical	734899
Cirrhosis	Identical	645992
(Remote) Monitoring AND Cirrhosis	1. AND 2.	7479
**Concept**	**Search string 29-11-2024 Web of Science**	**Results**
Remote Monitoring	TS=(“personal digital assistant” or "Digital Health" or "Mobile application" or "ambulatory monitoring" or "Point-of-Care System" or "Rapid Test" or "Rapid On-site Evaluation" or "Telemedicine" or "Telemetry" or "Videoconferencing" or (App or Apps or Desktop or (Digital NEAR/2 (care* or consult* or diagnos* or health* or medicine or monitor* or rehab* or therap* or visit*)) or Ehealth* or “E-health*” or “E-counseling” or “E-Therap*” or (Electronic* NEAR/2 (app* or health* or medicine*)) or Emedicine or “E-medicine” or EncephalApp or "Distan* Counsel*" or ((Handheld OR "Hand Held") NEAR/2 (computer* or device*)) or “Health app*” or (Home NEAR/2 (medicine or monitor*)) or iPad or Laptop or (Medical NEAR/2 (app*)) or Mhealth or “M-health*” or (Mobile NEAR/2 (app* or computer or device* or health* or medicine* or monitor*)) or "Outpatient Monitor*" or "Patient* Monitor*" or ("Point-of-care" NEAR/2 (test* or diagnos*)) or "Rapid Diagnos*" or "Rapid on-site diagnos*" or "Rapid on-site evaluation" or (Remote NEAR/2 (care* or consul* or diagnos* or health* or manag* or medicine* or monitor* or rehab*)) or Smartphone* or “smart-phone*” or Smartwatch or Tablet or Telecare* or Teleconsul* or Telediagnos* or Telehealth* or Telemedicine* or Telemonitor* or Telemetr* or “Tele-metr*” or Telerehab* or Telerefer* or Teletherap* or Televisit* or (Tele NEAR/2 (care* or consult* or diagnos* or health* or manag* or medicine* or monitor* or rehab* or refer* or therap* or visit*)) or Thealth* or “T-health*” or Videoconsult* or “Video Consult*” or Videoconf* or “Video-conf*”	851415

	or (Virtual NEAR/2 (care* or clinic* or consult* or health* or medicine* or monitor* or rehab* or therap* or visit*)) or Watch))	
Cirrhosis	TS=("Liver failure" or "Hepatopulmonary syndrome" or "Hepatorenal syndrome" or "Portal hypertension" or "Liver cirrhosis" or Ascites or (Ascites or Cirrh* or (Chronic* NEAR/2 (liver or hepatic*)) or CLD or Encephalopath* or (("end stage" or "late stage") NEAR/2 (liver* or hepatic*)) or ESLD or (esophag* NEAR/2 (varix* or varic*)) or (oesophag* NEAR/2 (varix* or varic*)) or (gastric* NEAR/2 (varix* or varic*)) or ((liver or hepatic*) NEAR/2 (fibrosis or fail* or insufficien* or dysfunction*)) or "hepatopulmonary syndrome*" or "hepato-pulmonary syndrome*" or "hepatorenal syndrome*" or "hepato-renal syndrome*" or "portal hypertension" or (varic* NEAR/2 (hemorrhag* or bleed*)) or (varix* NEAR/2 (hemorrhag* or bleed*))))	348725
(Remote) Monitoring AND Cirrhosis	1. AND 2.	2761
**Concept**	**Search string 14-07-2025 Web of Science**	**Results**
Remote Monitoring	Identical	902493
Cirrhosis	Identical	362681
(Remote) Monitoring AND Cirrhosis	1. AND 2.	2966
**Concept**	**Search string 13-01-2026 Web of Science**	**Results**
Remote Monitoring	Identical	943636
Cirrhosis	Identical	373250
(Remote) Monitoring AND Cirrhosis	1. AND 2.	3144
**Concept**	**Search string 29-11-2024 Cochrane Library**	**Results**

Remote Monitoring	(personal NEXT digital NEXT assistant or Digital NEXT Health or Mobile NEXT application or ambulatory NEXT monitoring or Point NEXT of NEXT Care NEXT System or Rapid NEXT Test or Rapid NEXT On NEXT site NEXT Evaluation or Telemedicine or Telemetry or Videoconferencing or (App or Apps or Desktop or (Digital NEAR/3 (care* or consult* or diagnos* or health* or medicine or monitor* or rehab* or therap* or visit*)) or Ehealth* or E NEXT health* or E NEXT counseling or E NEXT Therap* or (Electronic* NEAR/3 (app* or health* or medicine*)) or Emedicine or E NEXT medicine or EncephalApp or Distan* NEXT Counsel* or ((Handheld OR Hand NEXT Held) NEAR/3 (computer* or device*)) or Health NEXT app* or (Home NEAR/3 (medicine or monitor*)) or iPad or Laptop or (Medical NEAR/3 (app*)) or Mhealth or M NEXT health* or (Mobile NEAR/3 (app* or computer or device* or health* or medicine* or monitor*)) or Outpatient NEXT Monitor* or Patient* NEXT Monitor* or (Point NEXT of NEXT care NEAR/3 (test* or diagnos*)) or Rapid NEXT Diagnos* or Rapid NEXT on NEXT site NEXT diagnos* or Rapid NEXT on NEXT site NEXT evaluation or (Remote NEAR/3 (care* or consul* or diagnos* or health* or manag* or medicine* or monitor* or rehab*)) or Smartphone* or smart NEXT phone* or Smartwatch or Tablet or Telecare* or Teleconsul* or Telediagnos* or Telehealth* or Telemedicine* or Telemonitor* or Telemetr* or Tele NEXT metr* or Telerehab* or Telerefer* or Teletherap* or Televisit* or (Tele NEAR/3 (care* or consult* or diagnos* or health* or manag* or medicine* or monitor* or rehab* or refer* or therap* or visit*)) or Thealth* or T NEXT health* or Videoconsult* or Video NEXT Consult* or Videoconf* or Vide NEXT conf* or (Virtual NEAR/3 (care* or clinic* or consult* or health* or medicine* or monitor* or rehab* or therap* or visit*)) or Watch)):ti,ab,kw	102397
Cirrhosis	(Liver NEXT failure or Hepatopulmonary NEXT syndrome or Hepatorenal NEXT syndrome or Portal NEXT hypertension or Liver NEXT cirrhosis or Ascites or (Ascites or Cirrh* or (Chronic* NEAR/3 (liver or hepatic*)) or CLD or Encephalopath* or ((end NEXT stage or late NEXT stage) NEAR/3 (liver* or hepatic*)) or ESLD or (esophag* NEAR/3 (varix* or varic*)) or (oesophag* NEAR/3 (varix* or varic*)) or (gastric* NEAR/3 (varix* or varic*)) or ((liver or hepatic*) NEAR/3 (fibrosis or fail* or insufficien* or dysfunction*)) or hepatopulmonary NEXT syndrome* or hepato-pulmonary NEXT syndrome* or hepatorenal NEXT syndrome* or hepato-renal NEXT syndrome* or portal NEXT hypertension or (varic* NEAR/3 (hemorrhag* or bleed*)) or (varix* NEAR/3 (hemorrhag* or bleed*)))):ti,ab,kw	27729
(Remote) Monitoring AND Cirrhosis	1. AND 2.	1262
**Concept**	**Search string 14-07-2025 Cochrane Library**	**Results**
Remote Monitoring	Identical	137492

Cirrhosis	Identical	28218
(Remote) Monitoring AND Cirrhosis	1. AND 2.	1686
**Concept**	**Search string 14-01-2025 Cochrane Library**	**Results**
Remote Monitoring	Identical	144869
Cirrhosis	Identical	29478
(Remote) Monitoring AND Cirrhosis	1. AND 2.	1778
**Concept**	**Search string 29-11-2024 CINAHL via EBSCO**	**Results**
Remote Monitoring	MH("Computers, Hand-held+" or "Digital Health+" or "Mobile applications+" or "Monitoring, Physiologic+" " or "Clinical Information Systems+" or "Rapid Diagnostic Tests+" or "Rapid On-site Evaluation+" or "Telemedicine+" or "Telemetry" or "Videoconferencing") or TI(App or Apps or Desktop or (Digital N2 (care* or consult* or diagnos* or health* or medicine or monitor* or rehab* or therap* or visit*)) or Ehealth* or "E-health*" or "E-counseling" or "E-Therap*" or (Electronic* N2 (app* or health* or medicine*)) or Emedicine or "E-medicine" or EncephalApp or "Distan* Counsel*" or ((Handheld OR "Hand Held") N2 (computer* or device*)) or "Health app*" or (Home N2 (medicine or monitor*)) or iPad or Laptop or (Medical N2 (app*)) or Mhealth or "M-health*" or (Mobile N2 (app* or computer or device* or health* or medicine* or monitor*)) or "Outpatient Monitor*" or "Patient* Monitor*" or ("Point-of-care" N2 (test* or diagnos*)) or "Rapid Diagnos*" or "Rapid on-site diagnos*" or "Rapid on-site evaluation" or (Remote N2 (care* or consul* or diagnos* or health* or manag* or medicine* or monitor* or rehab*)) or Smartphone* or "smart-phone*" or Smartwatch or Tablet or Telecare* or Teleconsul* or Telediagnos* or Telehealth* or Telemedicine* or Telemonitor* or Telemetr* or "Tele-metr*" or Telerehab* or Telerefer* or Teletherap* or Televisit* or (Tele N2 (care* or consult* or diagnos* or health* or manag* or medicine* or monitor* or rehab* or refer* or therap* or visit*)) or Thealth* or "T-health*" or Videoconsult* or "Video Consult*" or Videoconf* or "Video-conf*" or (Virtual N2 (care* or clinic* or consult* or health* or medicine* or monitor* or rehab* or therap* or visit*)) or Watch) or AB(App or Apps or Desktop or (Digital N2 (care* or consult* or diagnos* or health* or medicine or monitor* or rehab* or therap* or visit*)) or Ehealth* or "E-health*" or "E-counseling" or "E-Therap*" or (Electronic* N2 (app* or health* or medicine*)) or Emedicine or "E-medicine" or EncephalApp or "Distan* Counsel*" or ((Handheld OR "Hand Held") N2 (computer* or device*)) or "Health app*" or (Home N2 (medicine or monitor*)) or iPad or Laptop or (Medical N2 (app*)) or	50652

	Mhealth or “M-health*” or (Mobile N2 (app* or computer or device* or health* or medicine* or monitor*)) or "Outpatient Monitor*" or "Patient* Monitor*" or ("Point-of-care" N2 (test* or diagnos*)) or "Rapid Diagnos*" or "Rapid on-site diagnos*" or "Rapid on-site evaluation" or (Remote N2 (care* or consul* or diagnos* or health* or manag* or medicine* or monitor* or rehab*)) or Smartphone* or “smart-phone*” or Smartwatch or Tablet or Telecare* or Teleconsul* or Telediagnos* or Telehealth* or Telemedicine* or Telemonitor* or Telemetr* or “Tele-metr*” or Telerehab* or Telerefer* or Teletherap* or Televisit* or (Tele N2 (care* or consult* or diagnos* or health* or manag* or medicine* or monitor* or rehab* or refer* or therap* or visit*)) or Thealth* or “T-health*” or Videoconsult* or “Video Consult*” or Videoconf* or “Video-conf*” or (Virtual N2 (care* or clinic* or consult* or health* or medicine* or monitor* or rehab* or therap* or visit*)) or Watch)	
Cirrhosis	(MH ("Liver Diseases+" OR "Hepatopulmonary syndrome+" or "Hepatorenal syndrome+" or "Hypertension, portal+" or "Liver cirrhosis+" or Ascites+) or TI(Ascites or Cirrh* or (Chronic* N2 (liver or hepatic*)) or CLD or Encephalopath* or (("end stage" or "late stage") N2 (liver* or hepatic*)) or ESLD or (esophag* N2 (varix* or varic*)) or (oesophag* N2 (varix* or varic*)) or (gastric* N2 (varix* or varic*)) or ((liver or hepatic*) N2 (fibrosis or fail* or insufficien* or dysfunction*)) or "hepatopulmonary syndrome*" or "hepato-pulmonary syndrome*" or "hepatorenal syndrome*" or "hepato-renal syndrome*" or "portal hypertension" or (varic* N2 (hemorrhag* or bleed*)) or (varix* N2 (hemorrhag* or bleed*))) OR AB (Ascites or Cirrh* or (Chronic* N2 (liver or hepatic*)) or CLD or Encephalopath* or (("end stage" or "late stage") N2 (liver* or hepatic*)) or ESLD or (esophag* N2 (varix* or varic*)) or (oesophag* N2 (varix* or varic*)) or (gastric* N2 (varix* or varic*)) or ((liver or hepatic*) N2 (fibrosis or fail* or insufficien* or dysfunction*)) or "hepatopulmonary syndrome*" or "hepato-pulmonary syndrome*" or "hepatorenal syndrome*" or "hepato-renal syndrome*" or "portal hypertension" or (varic* N2 (hemorrhag* or bleed*)) or (varix* N2 (hemorrhag* or bleed*))))	108026
(Remote) Monitoring AND Cirrhosis	1. AND 2.	239
**Concept**	**Search string 14-07-2025 CINAHL via EBSCO**	**Results**
Remote Monitoring	Identical	55277
Cirrhosis	Identical	109447
(Remote) Monitoring AND Cirrhosis	1. AND 2.	274
**Concept**	**Search string 13-01-2026 CINAHL via EBSCO**	**Results**
Remote Monitoring	Identical	61181

Cirrhosis	Identical	111871
(Remote) Monitoring AND Cirrhosis	1. AND 2.	309
**Concept**	**Search string 29-11-2024 Epistemonikos**	**Results**
Remote Monitoring	title:(App or Apps or Desktop or (Digital AND (care* or consult* or diagnos* or health* or medicine or monitor* or rehab* or therap* or visit*)) or Ehealth* or “E-health*” or “E-counseling” or “E-Therap*” or (Electronic* AND (app* or health* or medicine*)) or Emedicine or “E-medicine” or EncephalApp or "Distan* Counsel*" or ((Handheld OR "Hand Held") AND (computer* or device*)) or “Health app*” or (Home AND (medicine or monitor*)) or iPad or Laptop or (Medical AND (app*)) or Mhealth or “M-health*” or (Mobile AND (app* or computer or device* or health* or medicine* or monitor*)) or "Outpatient Monitor*" or "Patient* Monitor*" or ("Point-of-care" AND (test* or diagnos*)) or "Rapid Diagnos*" or "Rapid on-site diagnos*" or "Rapid on-site evaluation" or (Remote AND (care* or consul* or diagnos* or health* or manag* or medicine* or monitor* or rehab*)) or Smartphone* or “smart-phone*” or Smartwatch or Tablet or Telecare* or Teleconsul* or Telediagnos* or Telehealth* or Telemedicine* or Telemonitor* or Telemetr* or “Tele-metr*” or Telerehab* or Telerefer* or Teletherap* or Televisit* or (Tele AND (care* or consult* or diagnos* or health* or manag* or medicine* or monitor* or rehab* or refer* or therap* or visit*)) or Thealth* or “T-health*” or Videoconsult* or “Video Consult*” or Videoconf* or “Video-conf*” or (Virtual AND (care* or clinic* or consult* or health* or medicine* or monitor* or rehab* or therap* or visit*)) or Watch) or abstract:(App or Apps or Desktop or (Digital AND (care* or consult* or diagnos* or health* or medicine or monitor* or rehab* or therap* or visit*)) or Ehealth* or “E-health*” or “E-counseling” or “E-Therap*” or (Electronic* AND (app* or health* or medicine*)) or Emedicine or “E-medicine” or EncephalApp or "Distan* Counsel*" or ((Handheld OR "Hand Held") AND (computer* or device*)) or “Health app*” or (Home AND (medicine or monitor*)) or iPad or Laptop or (Medical AND (app*)) or Mhealth or “M-health*” or (Mobile AND (app* or computer or device* or health* or medicine* or monitor*)) or "Outpatient Monitor*" or "Patient* Monitor*" or ("Point-of-care" AND (test* or diagnos*)) or "Rapid Diagnos*" or "Rapid on-site diagnos*" or "Rapid on-site evaluation" or (Remote AND (care* or consul* or diagnos* or health* or manag* or medicine* or monitor* or rehab*)) or Smartphone* or “smart-phone*” or Smartwatch or Tablet or Telecare* or Teleconsul* or Telediagnos* or Telehealth* or Telemedicine* or Telemonitor* or Telemetr* or “Tele-metr*” or Telerehab* or Telerefer* or Teletherap* or Televisit* or (Tele AND (care* or consult* or diagnos* or health* or manag* or medicine* or monitor* or rehab* or refer* or therap* or visit*)) or Thealth* or “T-health*” or Videoconsult* or “Video Consult*” or Videoconf* or	318819

	"Video-conf*" or (Virtual AND (care* or clinic* or consult* or health* or medicine* or monitor* or rehab* or therap* or visit*)) or Watch)	
Cirrhosis	title:(Ascites or Cirrh* or (Chronic* AND (liver or hepatic*)) or CLD or Encephalopath* or (("end stage" or "late stage") AND (liver* or hepatic*)) or ESLD or (esophag* AND (varix* or varic*)) or (oesophag* AND (varix* or varic*)) or (gastric* AND (varix* or varic*)) or ((liver or hepatic*) AND (fibrosis or fail* or insufficien* or dysfunction*)) or "hepatopulmonary syndrome*" or "hepato-pulmonary syndrome*" or "hepatorenal syndrome*" or "hepato-renal syndrome*" or "portal hypertension" or (varic* AND (hemorrhag* or bleed*)) or (varix* AND (hemorrhag* or bleed*))) OR abstract:(Ascites or Cirrh* or (Chronic* AND (liver or hepatic*)) or CLD or Encephalopath* or (("end stage" or "late stage") AND (liver* or hepatic*)) or ESLD or (esophag* AND (varix* or varic*)) or (oesophag* AND (varix* or varic*)) or (gastric* AND (varix* or varic*)) or ((liver or hepatic*) AND (fibrosis or fail* or insufficien* or dysfunction*)) or "hepatopulmonary syndrome*" or "hepato-pulmonary syndrome*" or "hepatorenal syndrome*" or "hepato-renal syndrome*" or "portal hypertension" or (varic* AND (hemorrhag* or bleed*)) or (varix* AND (hemorrhag* or bleed*)))	111096
(Remote) Monitoring AND Cirrhosis	1. AND 2.	4217
**Concept**	**Search string 16-07-2025 Epistemonikos**	**Results**
Remote Monitoring	Identical	399366
Cirrhosis	Identical	132451
(Remote) Monitoring AND Cirrhosis	1. AND 2.	5839
**Concept**	**Search string 14-01-2026 Epistemonikos**	**Results**
Remote Monitoring	Identical	425826
Cirrhosis	Identical	137242
(Remote) Monitoring AND Cirrhosis	1. AND 2.	6112
**Concept**	**Search string 29-11-2024 PROSPERO**	**Results**

Remote Monitoring	(personal digital assistant OR Digital Health OR Mobile application OR ambulatory monitoring OR Point of Care System OR Rapid Test OR Rapid On site Evaluation OR Telemedicine OR Telemetry OR Videoconferencing OR App OR Apps OR Desktop OR (Digital NEAR2 (care* OR consult* OR diagnos* OR health* OR medicine OR monitOR* OR rehab* OR therap* OR visit*)) OR Ehealth* OR E health* OR E counseling OR E Therap* OR (Electronic* NEAR2 (app* OR health* OR medicine*)) OR Emedicine OR E medicine OR EncephalApp OR Distan* Counsel* OR ((Handheld OR Hand Held) NEAR2 (computer* OR device*)) OR Health app* OR (Home NEAR2 (medicine OR monitOR*)) OR iPad OR Laptop OR (Medical NEAR2 (app*)) OR Mhealth OR M health* OR (Mobile NEAR2 (app* OR computer OR device* OR health* OR medicine* OR monitOR*)) OR Outpatient MonitOR* OR Patient* MonitOR* OR (Point of care NEAR2 (test* OR diagnos*)) OR Rapid Diagnos* OR Rapid on site diagnos* OR Rapid on site evaluation OR (Remote NEAR2 (care* OR consul* OR diagnos* OR health* OR manag* OR medicine* OR monitOR* OR rehab*))) OR<br>(Smartphone* OR smart phone* OR Smartwatch OR Tablet OR Telecare* OR Teleconsul* OR Telediagnos* OR Telehealth* OR Telemedicine* OR TelemonitOR* OR Telemetr* OR Tele metr* OR Telerehab* OR Telerefer* OR Teletherap* OR Televisit* OR (Tele NEAR2 (care* OR consult* OR diagnos* OR health* OR manag* OR medicine* OR monitOR* OR rehab* OR refer* OR therap* OR visit*)) OR Thealth* OR T health* OR Videoconsult* OR Video Consult* OR Videoconf* OR Video conf* OR (Virtual NEAR2 (care* OR clinic* OR consult* OR health* OR medicine* OR monitOR* OR rehab* OR therap* OR visit*)) OR Watch)<br><br>1 OR 2	(15850)<br>(9445)<br><br>1 OR 2 →<br><br>20694
Cirrhosis	(Liver failure OR Hepatopulmonary syndrome OR Hepatorenal syndrome OR Portal hypertension OR Liver cirrhosis OR Ascites OR (Ascites OR Cirrh* OR (Chronic* NEAR2 (liver OR hepatic*)) OR CLD OR Encephalopath* OR ((end stage OR late stage) NEAR2 (liver* OR hepatic*)) OR ESLD OR (esophag* NEAR2 (varix* OR varic*)) OR (oesophag* NEAR2 (varix* OR varic*)) OR (gastric* NEAR2 (varix* OR varic*)) OR ((liver OR hepatic*) NEAR2 (fibrosis OR fail* OR insufficien* OR dysfunction*)) OR hepatopulmonary syndrome* OR hepato pulmonary syndrome* OR hepatorenal syndrome* OR hepato renal syndrome* OR portal hypertension OR (varic* NEAR2 (hemorrhag* OR bleed*)) OR (varix* NEAR2 (hemorrhag* OR bleed*))))	5858
(Remote) Monitoring AND Cirrhosis	((1 OR 2) AND 3)	329
**Concept**	**Search string 14-07-2025 PROSPERO**	**Results**
Remote Monitoring	(personal digital assistant OR Digital Health OR Mobile application OR ambulatory monitoring OR Point of Care System OR Rapid	(14962)<br>(11180)

	Test OR Rapid On site Evaluation OR Telemedicine OR Telemetry OR Videoconferencing OR App OR Apps OR Desktop OR (Digital NEAR2 (care* OR consult* OR diagnos* OR health* OR medicine OR monitOR* OR rehab* OR therap* OR visit*)) OR Ehealth* OR E health* OR E counseling OR E Therap* OR (Electronic* NEAR2 (app* OR health* OR medicine*)) OR Emedicine OR E medicine OR EncephalApp OR Distan* Counsel* OR **((Handheld OR Held) NEAR2 (computer* OR device*))** OR Health app* OR (Home NEAR2 (medicine OR monitOR*)) OR iPad OR Laptop OR (Medical NEAR2 (app*)) OR Mhealth OR M health* OR (Mobile NEAR2 (app* OR computer OR device* OR health* OR medicine* OR monitOR*)) OR Outpatient MonitOR* OR Patient* MonitOR* OR (Point of care NEAR2 (test* OR diagnos*)) OR Rapid Diagnos* OR Rapid on site diagnos* OR Rapid on site evaluation OR (Remote NEAR2 (care* OR consul* OR diagnos* OR health* OR manag* OR medicine* OR monitOR* OR rehab*))) OR<br>(Smartphone* OR smart phone* OR Smartwatch OR Tablet OR Telecare* OR Teleconsul* OR Telediagnos* OR Telehealth* OR Telemedicine* OR TelemonitOR* OR Telemetr* OR Tele metr* OR Telerehab* OR Telerefer* OR Teletherap* OR Televisit* OR (Tele NEAR2 (care* OR consult* OR diagnos* OR health* OR manag* OR medicine* OR monitOR* OR rehab* OR refer* OR therap* OR visit*)) OR Thealth* OR T health* OR Videoconsult* OR Video Consult* OR Videoconf* OR Video conf* OR (Virtual NEAR2 (care* OR clinic* OR consult* OR health* OR medicine* OR monitOR* OR rehab* OR therap* OR visit*)) OR Watch)<br><br>1 OR 2	1 OR 2 →<br><br>20095
Cirrhosis	(Liver failure OR Hepatopulmonary syndrome OR Hepatorenal syndrome OR Portal hypertension OR Liver cirrhosis OR Ascites OR (Ascites OR Cirrh* OR (Chronic* NEAR2 (liver OR hepatic*)) OR CLD OR Encephalopath* OR **((end OR late) NEAR3** (liver* OR hepatic*)) OR ESLD OR (esophag* NEAR2 (varix* OR varic*)) OR (oesophag* NEAR2 (varix* OR varic*)) OR (gastric* NEAR2 (varix* OR varic*)) OR ((liver OR hepatic*) NEAR2 (fibrosis OR fail* OR insufficien* OR dysfunction*)) OR hepatopulmonary syndrome* OR hepato pulmonary syndrome* OR hepatorenal syndrome* OR hepato renal syndrome* OR portal hypertension OR (varic* NEAR2 (hemorrhag* OR bleed*)) OR (varix* NEAR2 (hemorrhag* OR bleed*))))	6399
(Remote) Monitoring AND Cirrhosis	((1 OR 2) AND 3)	210
**Concept**	**Search string 13-01-2026 PROSPERO**	**Results**
Remote Monitoring	Identical to 14-07-2025<br><br>1 OR 2	(18128)<br>(13300)<br><br>1 OR 2 →

		24021
Cirrhosis	Identical to 14-07-2025	7473
(Remote) Monitoring AND Cirrhosis	((1 OR 2) AND 3)	239
**Concept**	**Search string 29-11-2024 ClinicalTrials.gov**	**Results**
Remote Monitoring<br><br>Intervention	(“personal digital assistant” OR "Digital Health" OR "Mobile application" OR "ambulatory monitoring" OR "Point-of-Care System" OR "Rapid Test" OR "Rapid On-site Evaluation" OR "Telemedicine" OR "Telemetry" OR "Videoconferencing" OR (App OR Apps OR Desktop OR (Digital AND (care* OR consult* OR diagnos* OR health* OR medicine OR monitor* OR rehab* OR therap* OR visit*)) OR Ehealth* OR E-health* OR E-counseling OR E-Therap* OR (Electronic* AND (app* OR health* OR medicine*)) OR Emedicine OR E-medicine OR EncephalApp OR "Distan* Counsel*" OR ((Handheld OR "Hand Held") AND (computer* OR device*)) OR “Health app*” OR (Home AND (medicine OR monitor*)) OR iPad OR Laptop OR (Medical AND (app*)) OR Mhealth OR M-health* OR (Mobile AND (app* OR computer OR device* OR health* OR medicine* OR monitor*)) OR "Outpatient Monitor*" OR "Patient* Monitor*" OR ("Point-of-care" AND (test* OR diagnos*)) OR "Rapid Diagnos*" OR "Rapid on-site diagnos*" OR "Rapid on-site evaluation" OR (Remote AND (care* OR consul* OR diagnos* OR health* OR manag* OR medicine* OR monitor* OR rehab*)) OR Smartphone* OR smart-phone* OR Smartwatch OR Tablet OR Telecare* OR Teleconsul* OR Telediagnos* OR Telehealth* OR Telemedicine* OR Telemonitor* OR Telemetr* OR Tele-metr* OR Telerehab* OR Telerefer* OR Teletherap* OR Televisit* OR (Tele AND (care* OR consult* OR diagnos* OR health* OR manag* OR medicine* OR monitor* OR rehab* OR refer* OR therap* OR visit*)) OR Thealth* OR T-health* OR Videoconsult* OR “Video Consult*” OR Videoconf* OR Video-conf* OR (Virtual AND (care* OR clinic* OR consult* OR health* OR medicine* OR monitor* OR rehab* OR therap* OR visit*)) OR Watch))	64035
Cirrhosis<br><br>Condition	("Liver failure" OR "Hepatopulmonary syndrome" OR "Hepatorenal syndrome" OR "Portal hypertension" OR "Liver cirrhosis" OR Ascites OR (Ascites OR Cirrh* OR (Chronic* AND (liver OR hepatic*)) OR CLD OR Encephalopath* OR (("end stage" OR "late stage") AND (liver* OR hepatic*)) OR ESLD OR (esophag* AND (varix* OR varic*)) OR (oesophag* AND (varix* OR varic*)) OR (gastric* AND (varix* OR varic*)) OR ((liver OR hepatic*) AND (fibrosis OR fail* OR insufficien* OR dysfunction*)) OR "hepatopulmonary syndrome*" OR "hepato-pulmonary syndrome*" OR "hepatorenal syndrome*" OR "hepato-renal syndrome*" OR "portal hypertension" OR (varic* AND (hemorrhag* OR bleed*)) OR (varix* AND (hemorrhag* OR bleed*))))	6463

(Remote) Monitoring AND Cirrhosis	1. AND 2.	1173
**Concept**	**Search string 14-07-2025 ClinicalTrials.gov**	**Results**
Remote Monitoring<br><br>Intervention	Identical	67875
Cirrhosis<br><br>Condition	Identical	6753
(Remote) Monitoring AND Cirrhosis	1. AND 2.	1219
**Concept**	**Search string 13-01-2026 ClinicalTrials.gov**	**Results**
Remote Monitoring<br><br>Intervention	Identical	70473
Cirrhosis<br><br>Condition	Identical	6867
(Remote) Monitoring AND Cirrhosis	1. AND 2.	1182

Table S3. Deduplication strategies

<table>
<tr><td rowspan="4">1. Deduplication via Endnote with manual revision of marked duplicates.</td><td>Step 1 duplicates removed, selection on:
Author, year, title, secondary title (journal), DOI, reference type</td></tr>
<tr><td>Step 2 duplicates removed, selection on:
Year, title, reference type</td></tr>
<tr><td>Step 3 duplicates removed, selection on:
Title</td></tr>
<tr><td>Step 4 duplicates removed, selection on:
Year, author</td></tr>
<tr><td colspan="2">2. Deduplication via DedupEndnote with manual revision of marked duplicates.</td></tr>
<tr><td colspan="2">3. Deduplication via Covidence, manual throughout the screening process.</td></tr>
</table>

Table S4. Newcastle-Ottawa Scale Quality assessment scores of observational cohort studies

Design	Year	Author	Selection Q1	Selection Q2	Selection Q3	Selection Q4	Comparability Q5	Outcome Q6	Outcome Q7	Outcome Q8	Total score (0 – 9)	Overall quality
**Prospective cohort with control group**	2. 2023	Kazankov *et al.* (1)	1	1	1	1	1	1	0	1	7	Good
	3. 2024	Penrice *et al.* (2)	1	1	1	1	2	1	0	0	7	Good
	4. 2017	Khungar *et al.* (3) Abstract	1	1	1	1	2	1	0	0	7	Fair
	5. 2024	Ballesteros *et al.* (4) Abstract	1	1	1	1	1	1	0	0	6	Good
**Prospective cohort without control group**	6. 2022	Lin *et al.* (5)	1	0	1	1	0	1	1	1	6	Fair
	7. 2015	Thomson *et al.* (6)	1	0	1	1	0	1	1	1	6	Fair
	8. 2025	Ngu *et al.* (7)	1	0	1	1	0	1	0	1	5	Fair
	9. 2020	Bloom *et al.* (8)	1	0	1	1	0	1	1	0	5	Fair
	10. 2021	Qian *et al.* (9) Abstract	1	0	1	1	0	1	0	0	4	Fair
	11. 2018	Verma *et al.* (10) Abstract	1	0	1	1	0	1	0	0	4	Fair
	12. 2017	Ganapathy *et al.* (11)	1	0	1	1	0	1	0	1	5	Fair

Table S5. Clinical outcomes

Author	Clinical outcomes	Results (controls vs. intervention group)	Significance
1. Shaw, et al. (12)	N avoidable readmissions SOC vs. HIT (%)	19.8% vs. 10%	**p = 0.042**
	OR avoidable readmissions SOC vs HIT	2.14 (95% CI 1.01–4.54)	
	N all cause admissions SOC vs. HIT (%)	48% vs. 30%	**p = 0.005**
	Time to readmission SOC vs. HIT (days)	7.9 vs. 4.7	**p = 0.003**
	N urgent clinic visits arranged after initial engagement with the app	35 patients	
2. Kazankov, et al. (1)	N readmissions (%)	8 (40) vs. 5 (25)	
	N patients with readmissions ≥14 days (%)	4 (20) vs. 0	
	Total number of readmissions	13 vs. 8	
	Time to first readmission (days)	4 (20) vs. 0	
	ITU length of stay (days)	16 vs. 5	
	N unplanned LVP (%)	6 (30) vs. 1 (5)	
	N liver transplantation (%)	0 vs. 1 (5)	
	N deceased (%)	2 (10) vs. 1 (5)	
3. Penrice, et al. (2)	% 30-day readmissions	28 vs. 20 (OR: 0.46 [0.18, 1.97])	p = 0.29
	% 90-day readmissions	47 vs. 34 (OR: 0.59 [0.18, 1.93])	p = 0.38
	% liver-related 90-day readmissions	86 vs. 79	p = 0.63
	% HE and fluid imbalance readmissions	57 vs. 36	p = 0.27
	Median time to first readmission	18 vs. 35 days	p = 0.30
	Mean total number of hospitalized days in 90 days following index admission	4.9 vs. 3.4	p = 0.067
	% ≥2 or more readmissions	36.5 vs. 12	
	90-day mortality	7.7 vs. 2.4	
	1-year mortality	28 vs. 22 (HR 0.62 [0.20, 1.90])	
4. Khungar, et al. (3) Abstract	% 30-day readmissions	21 vs. 15.8	
	30-day potentially preventable readmissions	Not reported, not statistically significant	
	90-day potentially preventable readmissions due to HE and volume overload	33.8 vs 0	**p = 0.02**
5. Ballesteros, et al. (4) Abstract	% Emergency admissions	28 vs. 12	**p<0.001**
	% 30-day decompensation rate	87 vs. 58	**p<0.05**
	% 90-day decompensation rate	72 vs. 47	**p<0.05**
	% 90-day recompensation rate	6 vs. 26	**p<0.05**
	% LVP	15 vs. 65	**p<0.001**
	% 90-day liver-related deaths	10 vs. 5	**p<0.05**
6. Lin, et al. (5)	N readmissions (%)	64 (55.2)	
	N deceased (%)	17 (14.7)	
7. Thomson, et al. (6)	N readmissions (%)	49 (62)	
	Time to first hospital admission (days), censoring death or transplantation	534	

	Hospitalization rate (admissions/follow-up)	1/5.8 months	
	N deceased (%)	20 (25)	
8. Ngu, *et al.* (7)	N 30-day readmissions (%)	9 (15)	
	N 90-day readmissions (%)	16 (27)	
	N 30-day deceased (%)	2 (3)	
	N 90-day deceased (%)	2 (3)	
9. Bloom, et al. (8)	N readmissions (%)	17 (68)	
	N LVP	13	
10. Qian, et al. (9) Abstract	N readmissions (%)	3 (6.25)	
	Clinic visits/patient pre vs. post enrollment	2.4 vs. 1.9	
	Hospitalizations/patient pre vs. post enrollment	0.5 vs. 0.3	
11. Verma, et al. (10) Abstract	N scheduled visits due to system flags (%)	13 (2.5)	
	N emergency visits due to system flags (%)	2 (0.4)	
	N planned hospital admissions due to system flags (%)	20 (3.9)	
12. Ganapathy, et al. (11)	N 30-day readmissions (%)	17 (42.5)	

HIT: Health Information Technology, ITU: Intensive Therapy Unit, LVP: Large Volume Paracentesis, SOC: Standard Of Care

Table S6. RM development quality assessment, according to V3+ framework

Year		Author	Verification	Usability validation	Analytical validation	Clinical validation
			Answers the verification question: Are the sensor data accurate, precise, uniform, and consistent?	Answers the usability validation question: Is the tool easy, efficient, and satisfying to use?	Answers the analytical validation question: Does the algorithm capture the outcome it claims to measure?	Answers the clinical validation question: Are the data informative to answer the specific clinical question in the intended context of use?*
1.	2025	Shaw, et al. (12)	1	1	1	1
2.	2023	Kazankov, et al. (1)	1	1	1	1
3.	2024	Penrice, et al. (2)	NR	1	1	1
4.	2017	Khungar, et al. (3) Abstract	NR	NR	NR	1
5.	2024	Ballesteros, et al. (4) Abstract	1	NR	NR	1
6.	2022	Lin, et al. (5)	1	NR	1	1
7.	2015	Thomson, et al. (6)	NR	1	NR	1
8.	2025	Ngu, *et al.* (7)	NR	1	NR	1
9.	2020	Bloom, et al. (8)	1	1	1	1
10.	2021	Qian, et al. (9) Abstract	NR	NR	NR	1
11.	2018	Verma, et al. (10) Abstract	NR	1	1	1
12.	2017	Ganapathy, et al. (11)	1	1	1	1
**Total**			6	8	7	12

*Measurement of at least one clinical outcome was an inclusion criterium of this systematic review.

Material S1. Newcastle-Ottawa Scale Quality assessment scores of observational cohort studies (13)

NEWCASTLE - OTTAWA QUALITY ASSESSMENT SCALE
COHORT STUDIES

Note: A study can be awarded a maximum of one star for each numbered item within the Selection and Outcome categories. A maximum of two stars can be given for Comparability

Selection

1) Representativeness of the exposed cohort
   a) truly representative of the average **liver cirrhosis patient (compensated or decompensated)** (describe) in the community ✵
   b) somewhat representative of the average **liver cirrhosis patient (compensated or decompensated)** in the community ✵
   c) selected group of users eg nurses, volunteers
   d) no description of the derivation of the cohort

2) Selection of the non exposed cohort
   a) drawn from the same community as the exposed cohort ✵
   b) drawn from a different source
   c) no description of the derivation of the non exposed cohort

3) Ascertainment of exposure
   a) secure record (eg surgical records) ✵
   b) structured interview ✵
   c) written self report
   d) no description

4) Demonstration that outcome of interest was not present at start of study
   a) yes ✵
   b) no

Comparability

1) Comparability of cohorts on the basis of the design or analysis
   a) study controls for (select the most important factor): ✵
   Clinically compensated or decompensated state, according to Baveno VII classification.
   b) study controls for any additional factor ✵ (This criteria could be modified to indicate specific control for a second important factor.)
   MELD-score, Child-Pugh score, age, sex, liver cirrhosis etiology, socioeconomic status surrogates such as education, income, zip code

Outcome

1) Assessment of outcome
   a) independent blind assessment ✵
   b) record linkage ✵
   c) self report
   d) no description

2) Was follow-up long enough for outcomes to occur
   a) yes (select an adequate follow up period for outcome of interest) ✵
   Adequate follow-up period per outcome of interest for the specific study:
   - **Hospital readmission: 3 months**

- **Feasibility: 6 months**
- **Clinical outcome decompensated outpatients: 6 months**
- **Clinical outcomes compensated outpatients: 12 months**

b) no

3) Adequacy of follow up of cohorts

a) complete follow up - all subjects accounted for ✸

b) subjects lost to follow up unlikely to introduce bias - small number lost - > **≥75%** (select an adequate %) follow up, or description provided of those lost) ✸

c) follow up rate **<75%** (select an adequate %) and no description of those lost

d) no statement

Figure S1. Cochrane Risk of Bias Assessment Tool of RCT’s (Rob2) (14)

Year	Author	D1	D2	D3	D4	D5	Overall
1. 2025	Shaw *et al. (12)*	+	+	+	+	+	+

+	Low risk
!	Some concerns
-	High risk
D1	Randomisation process
D2	Deviations from the intended interventions
D3	Missing outcome data
D4	Measurement of the outcome
D5	Selection of the reported result

References

1. Kazankov K, Novelli S, Chatterjee DA, Phillips A, Balaji A, Raja M, et al. Evaluation of CirrhoCare R - a digital health solution for home management of individuals with cirrhosis. Journal of hepatology. 2023;78(1):123-32.
2. Penrice DD, Hara KS, Sordi-Chara B, Kezer C, Schmidt K, Kassmeyer B, et al. Design, implementation, and impact of a cirrhosis-specific remote patient monitoring program. Hepatology communications. 2024;8(8).
3. Khungar V, Serper M, Peyton D, Mehta S, Norris A, Huffenberger A, et al. Use of an Innovative Telehealth Platform to Reduce Readmissions and Enable Patient-Centered Care in Cirrhotic Patients. HEPATOLOGY. 2017;66:94A-5A.
4. Ballesteros K, Kumaravel Kanagavelu AS, Crone J, Rahman R, Schlitzer A, Sharma V. P126 Improving patient outcomes in decompensated liver disease through remote monitoring: a real-world experience. Gut. 2024;73(Suppl 3):A90.
5. Lin F-P, Bloomer PM, Grubbs RK, Rockette-Wagner B, Tevar AD, Dunn MA, et al. Low Daily Step Count Is Associated With a High Risk of Hospital Admission and Death in Community-Dwelling Patients With Cirrhosis. Clinical gastroenterology and hepatology : the official clinical practice journal of the American Gastroenterological Association. 2022;20(8):1813-20.e2.
6. Thomson M, Volk M, Kim HM, Piette JD. An Automated Telephone Monitoring System to Identify Patients with Cirrhosis at Risk of Re-hospitalization. Digestive diseases and sciences. 2015;60(12):3563-9.
7. Ngu NLY, Saxby E, Worland T, Anderson P, Stothers L, Hunter J, et al. A Nonrandomized Pilot Study to Investigate the Acceptability and Feasibility of LivR Well: A Multifaceted 28-Day Home-Based Liver Optimization Program for Acute-on-Chronic Liver Failure. Gastro Hep Advances. 2025;4(2):100567.
8. Bloom P, Wang T, Marx M, Tagerman M, Green B, Arvind A, et al. A Smartphone App to Manage Cirrhotic Ascites Among Outpatients: Feasibility Study. JMIR Med Inform. 2020;8(9):e17770.
9. Qian LA, Higgins A, Zapatka S, Taddei TH, Garcia-Tsao G, Jakab SS. Development and feasibility of a disease management program for patients with cirrhosis at the veterans health administration (vha). Hepatology. 2021;74(SUPPL 1):375A.
10. Verma M, Kalman R, Walter JW, Gallagher M, Navarro V. FEASIBILITY OF TELEMONITORING OF SYMPTOMS AND COGNITIVE FUNCTION IN END STAGE LIVER DISEASE. GASTROENTEROLOGY. 2018;154(6):S1221-S2.
11. Ganapathy D, Acharya C, Lachar J, Patidar K, Sterling RK, White MB, et al. The patient buddy app can potentially prevent hepatic encephalopathy-related readmissions. Liver international : official journal of the International Association for the Study of the Liver. 2017;37(12):1843-51.
12. Shaw J, Acharya C, Fagan A, Olofson A, Irwin K, Kolhekar S, et al. Health information technology interventions reduce avoidable readmissions in cirrhosis: The HEROIC randomized controlled trial. Hepatology. 2025.
13. Wells G, Shea B, O'Connell D, Peterson J, Welch V, Losos M, et al. The Newcastle-Ottawa Scale (NOS) for assessing the quality of nonrandomised studies in meta-analyses [Available from: https://www.ohri.ca/programs/clinical_epidemiology/oxford.asp.
14. Sterne JAC, Savovic J, Page MJ, Elbers RG, Blencowe NS, Boutron I, et al. RoB 2: a revised tool for assessing risk of bias in randomised trials. BMJ. 2019;366:l4898.
